# The ABAP1 interacting protein 10 (AIP10) exerts a dual role in the cell cycle and primary metabolism pathways in *Arabidopsis thaliana*

**DOI:** 10.1111/tpj.70399

**Published:** 2025-08-08

**Authors:** Patrícia Montessoro, Joaquin Felipe Roca Paixão, Carinne N. M. Costa, Laura Ducatti, Letícia Perdigão Grangeiro, Vivian Ruivo, Adriana Flores Fusaro, Helkin F. Ballesteros, Vanessa Iurif, Luiz Mors Cabral, Jelmir Craveiro de Andrade, Leticia Tessaro, Wallace de Paula Bernado, Fernanda Silva Coelho, Bruna Gino de Araújo‐Lopes, Janice de Almeida Engler, Jérémie Bazin, Eliemar Campostrini, Carlos Adam Conte‐Junior, Adriana Silva Hemerly

**Affiliations:** ^1^ Laboratório de Biologia Molecular de Plantas, Instituto de Bioquímica Médica Universidade Federal do Rio de Janeiro Rio de Janeiro Rio de Janeiro Brazil; ^2^ Departamento de Biologia Celular e Molecular, Instituto de Biologia Universidade Federal Fluminense Niterói Brazil; ^3^ Laboratory of Advanced Analysis in Biochemistry and Molecular Biology (LAABBM), Department of Biochemistry Universidade Federal do Rio de Janeiro Rio de Janeiro Rio de Janeiro Brazil; ^4^ Setor Fisiologia Vegetal, Centro de Ciências e Tecnologias Agropecuárias (CCTA) Univ. Estadual Norte Fluminense (UENF) Campos dos Goytacazes Rio de Janeiro Brazil; ^5^ Departamento de Pesquisa e Desenvolvimento Empresa HapiSeeds Rio de Janeiro Rio de Janeiro Brazil; ^6^ INRAE Université Cote d'Azur, CNRS, ISA Sophia Antipolis 06903 France; ^7^ Institute of Plant Sciences Paris‐Saclay (IPS2), CNRS, INRAE Université Paris Saclay – Evry, Université de Paris, Gif sur Yvette France

**Keywords:** ABAP1, AIP10, cell cycle, biomass, plant development, photosynthesis, primary metabolism, SnRK1

## Abstract

Plants have developed a sophisticated regulatory network that coordinates gene expression in meristematic zones in response to environmental conditions. Here, we identified a protein in Arabidopsis (*Arabidopsis thaliana*) that interacts with Armadillo BTB Arabidopsis protein 1 (ABAP1), a negative regulator of the cell cycle in plants. We characterized the ABAP1 interacting protein (named AIP10) investigating its role in modulating plant development. T‐DNA insertion lines with silenced expression of *AIP10* were evaluated phenotypically (morphology, fresh and dry weight), via transcriptomics analyses (RNA‐Seq and RT‐qPCR), physiologically (biochemically, Fluorcam and Li‐COR) and metabolically (ATR‐FTIR). We showed that AIP10 integrates cell division rates with transcriptional and primary metabolism reprogramming through its protein interactions with ABAP1 and KIN10, a subunit of SnRK1 (sucrose non‐fermenting‐1‐related protein kinase 1). *ABAP1* levels and activity were reduced in the absence of *AIP10*, licensing cell cycle progression for longer periods, which culminated in increased rates of cell division that boosted vegetative and reproductive growth. *AIP10* knockout triggered a major transcriptional reprogramming of plant primary metabolism, possibly through SnRK1 modulation. *aip10* mutants showed increased photosynthetic efficiency, as well as boosted carbon fixation, leading to increased biomass, seed productivity, and higher contents of proteins, lipids (triglycerides), and carbohydrates. Finally, we propose that the modulation of *AIP10* expression is part of a mechanism that coordinates higher rates of cell division with better photosynthetic performance and carbon fixation to metabolically meet the plant energy demand, allowing the generation of plants with increased biomass and productivity.

## Significance Statement

Plants regulate cell division to adapt growth to environmental changes. In *Arabidopsis thaliana*, AIP10 acts as a hub linking the cell cycle to primary metabolism. Its silencing increases cell division rates, enhances carbon fixation, and boosts metabolite accumulation, driving improved development and productivity.

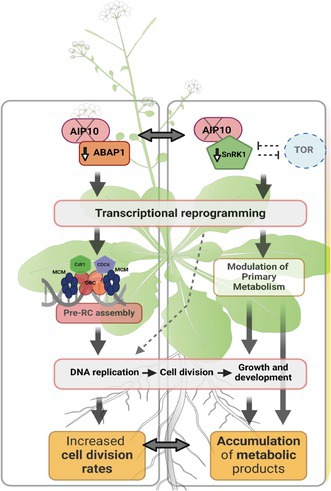

## INTRODUCTION

Plants have evolved distinct mechanisms to cope with environmental signals such as light, abiotic stress, or pathogen attacks, which influence their architecture and plasticity (Pierik et al., 2021). Developmental plasticity strategies consist in altering morphological and physiological aspects to promote growth, survival, and reproduction when confronted with adverse effects (Nawaz et al., 2023). The morphological response is mediated by the adjustment of cell division rates at self‐perpetuating meristems (Qi & Zhang, 2020). Cell division and differentiation at both root and shoot apical meristems are coordinated by hormone pathways, receptor kinase–peptide networks, and transcription factor signaling (Qi & Zhang, 2020).

A typical plant mitotic cell division is accomplished in a cell cycle consisting of four different phases, G1 (post‐mitotic interphase), S (DNA synthesis phase), G2 (premitotic interphase), and M (mitosis/cytokinesis). The progression between the different phases is coordinated by key regulators, such as cyclin‐dependent kinases (CDKs) complexed to cyclins (CYC), and checkpoint regulators, to ensure the cell cycle is ready to proceed to a new phase (Qi & Zhang, 2020). An important control that integrates the cell cycle with the environment is at the G1/S transition, when cells are licensed to initiate DNA replication, in a step regulated by the pre‐replication complex (pre‐RC) (Del Pozo et al., 2006). The pre‐RC interacts with DNA and/or chromatin marks through the origin recognition complex (ORC1‐to‐ORC6), that is assembled as a scaffold for sequential association of Cell Division Cycle 6 (CDC6), Chromatin Licensing and DNA Replication Factor 1 (CDT1), and Mini Chromosome Maintenance (MCM complex: MCM2–7), licensing DNA for replication (Brasil et al., 2017). Positive and negative proliferation signals, that often depend on environmental stimuli, control the response of the pre‐RC machinery (Bailis & Forsburg, 2004).

In plants, we have previously characterized Armadillo BTB Arabidopsis protein 1 (ABAP1) as a plant‐specific protein that binds directly to ORC1a/b and CDT1a/b in *Arabidopsis thaliana* (Masuda et al., 2008). ABAP1 negatively regulates the assembly of the pre‐RC, which reduces DNA replication licensing and cell division rates. Its mechanism of action also involves association with ABAP1 interacting proteins (named AIP) to reduce the expression of specific genes and negatively modulates cell proliferation rates during development (Brasil et al., 2015; Cabral et al., 2021; Masuda et al., 2008). Among the identified proteins interacting with ABAP1, some had unknown functions, such as AIP10. Previous studies evidenced putative orthologs of *AIP10* in *Physcomitrium patens* (*PpSKI1*, *PpSKI2*) and *Oryza sativa* (*OsHDR1*) that interact with SNF1/AMPK‐related protein kinase 1 (SnRK1) homologs, *Pp*Snf1a and *Os*K4 kinases, respectively, to regulate growth and photoperiodic flowering (Sun et al., 2016; Thelander et al., 2007). SnRK1 is a master regulator of energy homeostasis during sugar starvation, also controlling developmental plasticity and cellular responses to increase resilience under different stress conditions (Baena‐González & Lunn, 2020). In Arabidopsis, the catalytic subunits of SnRK1 are encoded by three genes: *SnRK1α1* (KIN10), *SnRK1α2* (KIN11), and *SnRK1α3* (KIN12) (Soto‐Burgos & Bassham, 2017).

Here, we describe an ABAP1 interactor that integrates modulation of the cell cycle and plant primary metabolism, adjusting plant plasticity to better respond to environmental signaling. We investigated the role of AIP10 in plant development by characterizing *A. thaliana* mutants with modified *AIP10* expression. Plants with silenced expression of *AIP10* (*aip10‐1*) showed higher rates of cell proliferation in the meristems, for longer periods, leading to a vegetative and reproductive increase. AIP10 together with ABAP1 negatively modulates cell division, influencing the expression of ABAP1 target genes and cell cycle progression. RNA‐Seq transcriptomics revealed that *AIP10* knockout leads to a major reprogramming of plant primary metabolism, possibly through modulation of the SnRK1 pathway. Furthermore, *AIP10* knockout increased CO_2_ assimilation, as well as carbon fixation, resulting in a higher content of proteins, lipids (triglycerides), and carbohydrates. Finally, we propose that the modulation of *AIP10* expression is part of a mechanism that coordinates higher rates of cell division with better photosynthetic performance and carbon fixation, to metabolically meet its energy demand, allowing the generation of plants with an increase in biomass and gain in productivity.

## RESULTS

### AIP10 interacts with ABAP1, an inhibitor of cell division in plants, and with SnRK1, a master regulator of plant responses to environmental stimuli

**Figure 1:**
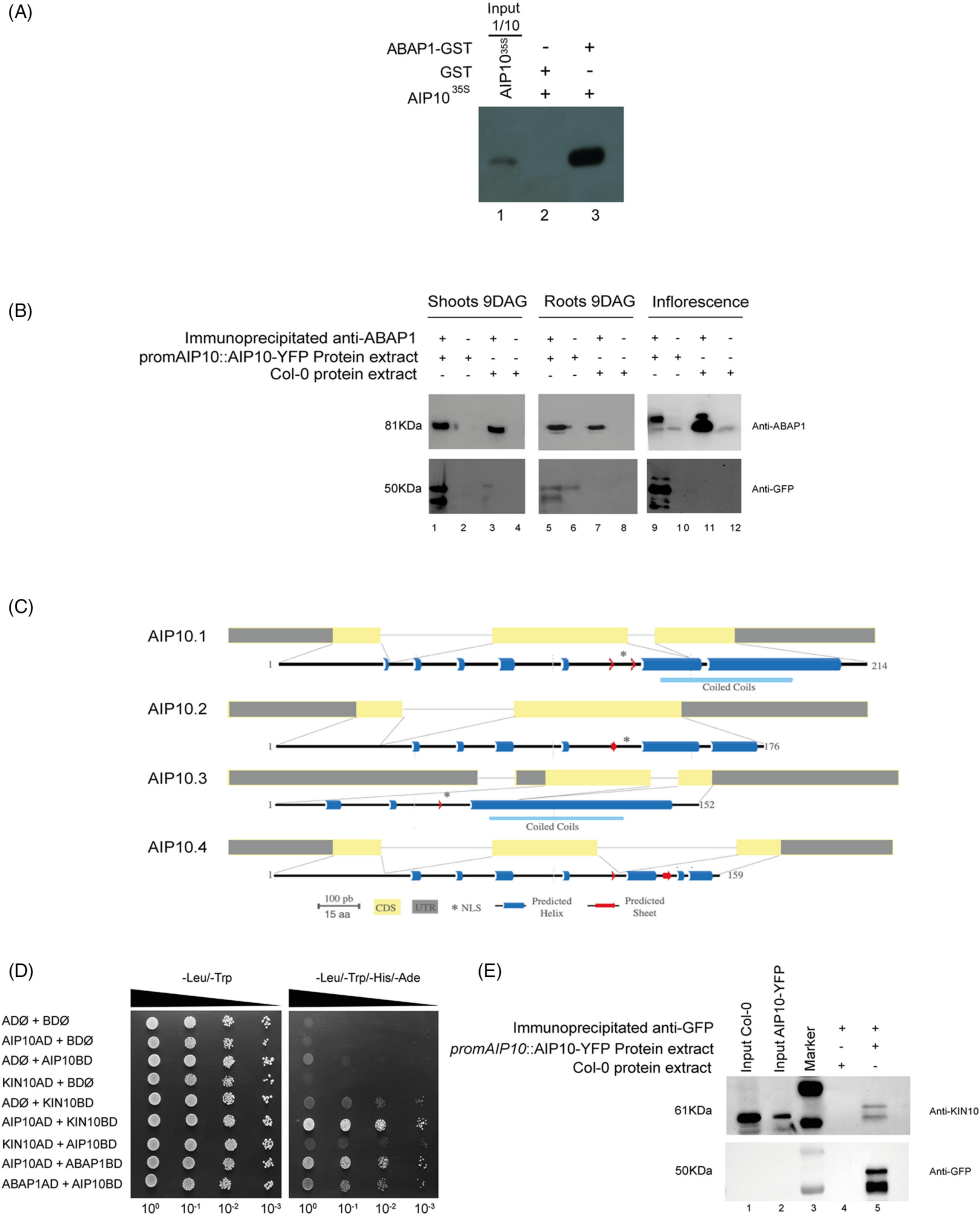
AIP10 interacts with ABAP1 *in vitro* and *in vivo*. (A) ABAP1–AIP10 interaction through *in vitro* GST‐pulldown assay of bacterially expressed recombinant ABAP1‐GST fusion tested against *in vitro*‐translated AIP10^S35^. ABAP1 and AIP10 interaction is shown in lane 3. (B) Co‐immunoprecipitation (co‐IP) of ABAP1 and protein extracts of plants expressing AIP10‐YFP. Reactive bands corresponding to ABAP1 and AIP10‐YFP were detected in shoot (lane 1), root (lane 5), and inflorescence (lane 9), by Western blot using anti‐ABAP1 and anti‐GFP antibodies, respectively. The GFP antibody also recognizes the YFP protein. Numbers 1–12 refer to lanes in the SDS‐PAGE. (C) Schematic representation of AIP10 splice variants, showing the exons as gray (UTR) and yellow (CDS) boxes. Predicted nuclear localization signal (NLS) is indicated as *, and predicted helix or sheet are indicated in blue or red, respectively. (D) Yeast two‐hybrid assay for protein interaction analysis between AIP10 and KIN10 fused to the AD and BD domains. Empty vectors pDEST22 (AD) and pDEST32 (BD) were used as negative controls, while AIP10 + ABAP1, fused to AD/BD, were used as a positive control. −Leucine/−Tryptophan media selected co‐transformation with both BD/AD constructs; −leucine/−tryptophan/−histidine/−adenine selected for strong protein interaction. AIP10AD showed strong interaction with KIN10BD. AD, activation domain; BD, binding domain; CDS, coding sequence; GFP, green fluorescent protein; GST, glutathione S‐transferase; UTR, untranslated region; YFP, yellow fluorescent protein. (e) Co‐immunoprecipitation (co‐IP) of KIN10 and protein extracts from plants expressing AIP10‐YFP. Reactive bands corresponding to KIN10 and AIP10‐YFP were detected in the shoot (lane 5) by Western blot using anti‐KIN10 and anti‐GFP antibodies, respectively. The GFP antibody also recognizes the YFP protein. Numbers 1–5 refer to the lanes on the SDS‐PAGE.

The ABAP1 interacting protein 10 (At1g80940) was part of a set of proteins found during a yeast two‐hybrid (Y2H) screening against an *A. thaliana* cDNA library (Brasil et al., 2015; Masuda et al., 2008), using ABAP1 as a bait. A series of assays were performed to confirm *in vitro* and *in vivo* interaction between AIP10 and ABAP1 (Figure 1; Figure S1). A direct Y2H assay using either AIP10 or ABAP1 as baits (BD) showed interaction between the two proteins (Figure S2). The same Y2H assay showed that AIP10 did not interact with all preRC members tested (ORC1‐6, CDC6 and CDT1, except MCM2–7), nor with ARIA, an ABAP1 homolog. Interactions between ABAP1 and AIP10 were confirmed both *semi‐in vivo* and *in vitro*, through GST (gluthatione *S*‐transferase) pulldown experiments. In *semi‐in vivo* using GST::AIP10 and ABAP1‐GFP protein extract, ABAP1 bound specifically to AIP10‐GST (lane 5), but not to GST alone (lane 1), as shown in Figure S1a. Complementarily, *in vitro* pulldown assays using GST::ABAP1 and *in vitro* translated AIP10^S35^ confirmed the interaction with AIP10 binding to ABAP1‐GST (lane 3), but not to GST alone (lane 2). We next performed an anti‐ABAP1 co‐immunoprecipitation assay with shoot, root, and inflorescence (Figure 1B) protein extracts of plants expressing *promAIP10::AIP10‐YFP*. These plants carry a construct *promAIP10*::AIP10‐YFP containing the full‐length genomic *AIP10* gene that gives rise to a fluorescently tagged AIP10‐YFP C‐terminal fusion protein (Tian et al., 2004). A protein blot with antibodies against YFP exhibited a major 50 kDa band at lanes 1 and 5, indicating the *in vivo* interaction between AIP10 and ABAP1 in different plant organs (Figure 1B). Other observed positive bands may represent isoforms of the AIP10 protein, or phosphorylated forms of the protein (see below, Figure 1B).

The *AIP10* gene generates four isoforms through alternative splicing (Figure 1C), in which all isoforms showed high expression levels in young leaves and reproductive organs (Figure S3). AIP10.1 has 214 amino acids (24 kDa), and AIP10.2, AIP10.3, and AIP10.4 are splice variants with 176 aa (19 kDa), 152 aa (17 kDa), and 159 aa (17 kDa), respectively. AIP10.1, AIP10.2, and AIP10.3 possess a nuclear localization sequence (NLS) and only AIP10.1 and AIP10.3 possess a predicted coiled coil domain on the C‐terminal part of the protein (Figure 1C).

AIP10 is a plant‐specific gene distributed all over the plant kingdom that has at least one copy in all the 136 terrestrial species analyzed (Figure S4a). ABAP1, besides being distributed all over the plant kingdom, has representatives also in algae, with 511 homologs present in 139 species (Figure S5). The AIP10 protein family is classified in the InterPro database as Heading Date Repressor 1 (HDR1) which regulates flowering time in rice in a photoperiod‐dependent way (Sun et al., 2016). Two homologs have already been described as *PpSKI1/2* (Snf1‐related kinase interacting protein 1/2) in *Physcomitrium patens* along with *OsHDR1* in *Oryza sativa* (Sun et al., 2016; Thelander et al., 2007). The alignment revealed conserved regions mainly at the C‐terminal part of the protein, including an NLS present in all the sequences (red frame), and all sequences presented putative phosphorylation sites (PPS) (Figure S4b).

A conserved sequence VDVVE**S**MRRI (green frame) adjacent to the NLS was identified, which corresponds to a canonical KIN10 phosphorylation site (MLVFI)X(RKH)XX(**S/T**)XXX(LFIMV) as described by Halford and Hardie (1998) (Figure S3b). In accordance, *Pp*SKI1/2 and *Os*HDR1 interact with *Pp*Snf1a and *Os*K4 kinases, respectively (Sun et al., 2016; Thelander et al., 2007). To test if the *A. thaliana* AIP10 interacts with SnRK1, yeast two‐hybrid assays against two catalytic subunits of the kinase, KIN10 and KIN11, were performed (Figure 1D; Figure S2). The data showed a direct interaction between AIP10 and KIN10 (Figure 1D) that was not seen for KIN11 (Figure S1).

To further investigate the association between AIP10 and KIN10, a *semi‐in vivo* pull‐down assay was performed usin*g* full‐length GST::AIP10 and protein extracts of Arabidopsis 9‐day‐old plantlets. As shown in Figure S1(b), AIP10 and KIN10 interaction was confirmed in *semi‐in vivo* GST (glutathione S‐transferase) pulldown experiments, as KIN10 bound to AIP10‐GST (lane 2) but not to GST alone (lane 1). We next performed a coimmunoprecipitation assay using anti‐KIN10 antibody with protein extracts from shoots of 9‐day‐old plants expressing *promAIP10*::AIP10‐YFP (Figure 1E). Western blot with anti‐KIN10 antibody revealed a band of approximately 61 kDa (lanes 1, 2, and 5), whereas anti‐YFP antibody detected a major band of ~50 kDa in lane 5, indicating the *in vivo* interaction between AIP10 and KIN10. The additional band observed with anti‐KIN10 antibody might represent a phosphorylated isoform of the protein, whereas the other positive bands detected with antibodies against YFP may represent isoforms of the AIP10 protein, or phosphorylated forms of the protein, as observed in Figure 1(E).

### AIP10 is predominantly expressed in the nuclei of cells from young organs

**Figure 2:**
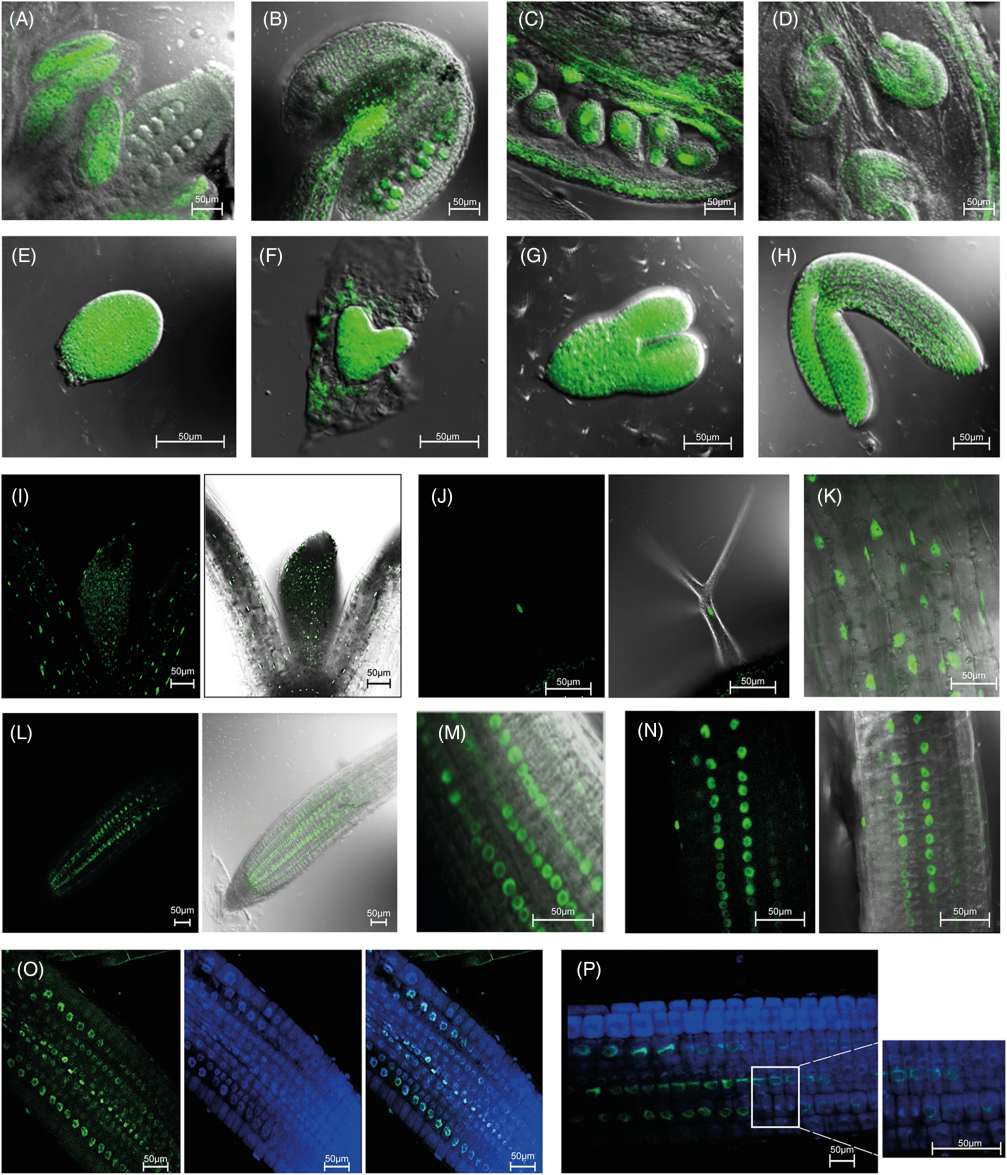
Tissue expression pattern and subcellular localization of fluorescent fusion protein AIP10‐YFP, in Arabidopsis expressing *promAIP10::AIP10‐YFP*. YFP signal was visualized by epifluorescence confocal microscopy. (A–H, K, M) Merged images of epifluorescence confocal microscopy and bright‐field differential interference contrast; (A, B) anthers; (C, D) ovaries; (E) globular embryo; (F) cordiform embryo, (G) torpedo embryo and (H) mature embryo. (I, J, L, N) Epifluorescence confocal image in left panel, merged with bright‐field differential interference contrast image in right panel; YFP signal in the nuclei of (I) SAM; (J) trichome; (K) hypocotyl, (L) RAM at 10 DAG; (M) nucleus and perinuclear region in root at 5 DAG; (N) nucleus and perinuclear region in root at 10 DAG. (O, P) epifluorescence confocal microscopy images of root cells stained with 4,6‐diamidino‐2‐phenylindole (DAPI) to visualize the nucleus; (O) YFP signal from RAM at 5 DAG; (P) magnification of RAM at 5 DAG showing that the protein is not present in dividing cells. Scale bars: 50 μm. RAM, root apical meristem; SAM, shoot apical meristem; YFP, yellow fluorescent protein.

*A. thaliana* plants expressing *promAIP10::*AIP10‐YFP were used to determine the expression of the AIP10 protein in plant tissues during development, as well as its subcellular localization, under the control of its own promoter. Confocal analysis showed that AIP10 is present in different organs and tissues, consistent with the expression data in the eFP Browser at the TAIR platform. During reproduction, AIP10 was detected in reproductive organs such as anthers (Figure 2A,B) and ovaries (Figure 2C,D), being present in male and female gametes. AIP10 has a constitutive pattern of expression at different stages of embryogenesis, as shown in globular (Figure 2E), cordiform (Figure 2F), torpedo (Figure 2G) and mature embryo (Figure 2H). It is present in the nuclei of cells in the cotyledon, in the first pair of leaves (Figure 2I), in the developing trichomes (Figure 2J) and in the nuclei of hypocotyl cells (Figure 2K). In roots, constitutive nuclear and perinuclear fluorescence was detected (Figure 2L,N). Fluorescence was not observed in the root cap but was present in the root apical meristem (RAM) nuclei (Figure 2L). AIP10 fluorescence intensity changes at the periphery of nuclei throughout the root (Figure 2M,N). To investigate the localization of AIP10 in dividing cells, nuclei were stained with DAPI (Figure 2O,P). The protein is located at the periphery of the nuclei in the different phases of the cell cycle (Figure 2O), being absent in mitotic cells (Figure 2P). Altogether, the data indicate that AIP10 is a protein present in all plant organs and meristems.

### Modulation of AIP10 levels influences plant vegetative growth, biomass, and productivity

To investigate the role of AIP10 in plant development, *A. thaliana* plants with altered AIP10 transcript levels were characterized. Two T‐DNA insertion lines, Salk_022332 and Salk_094618, with the AIP10 gene knockout (*aip10‐1*) or knockdown (*aip10‐2*) (Figure S6a,b), respectively, were used for phenotype comparisons.

**Figure 3:**
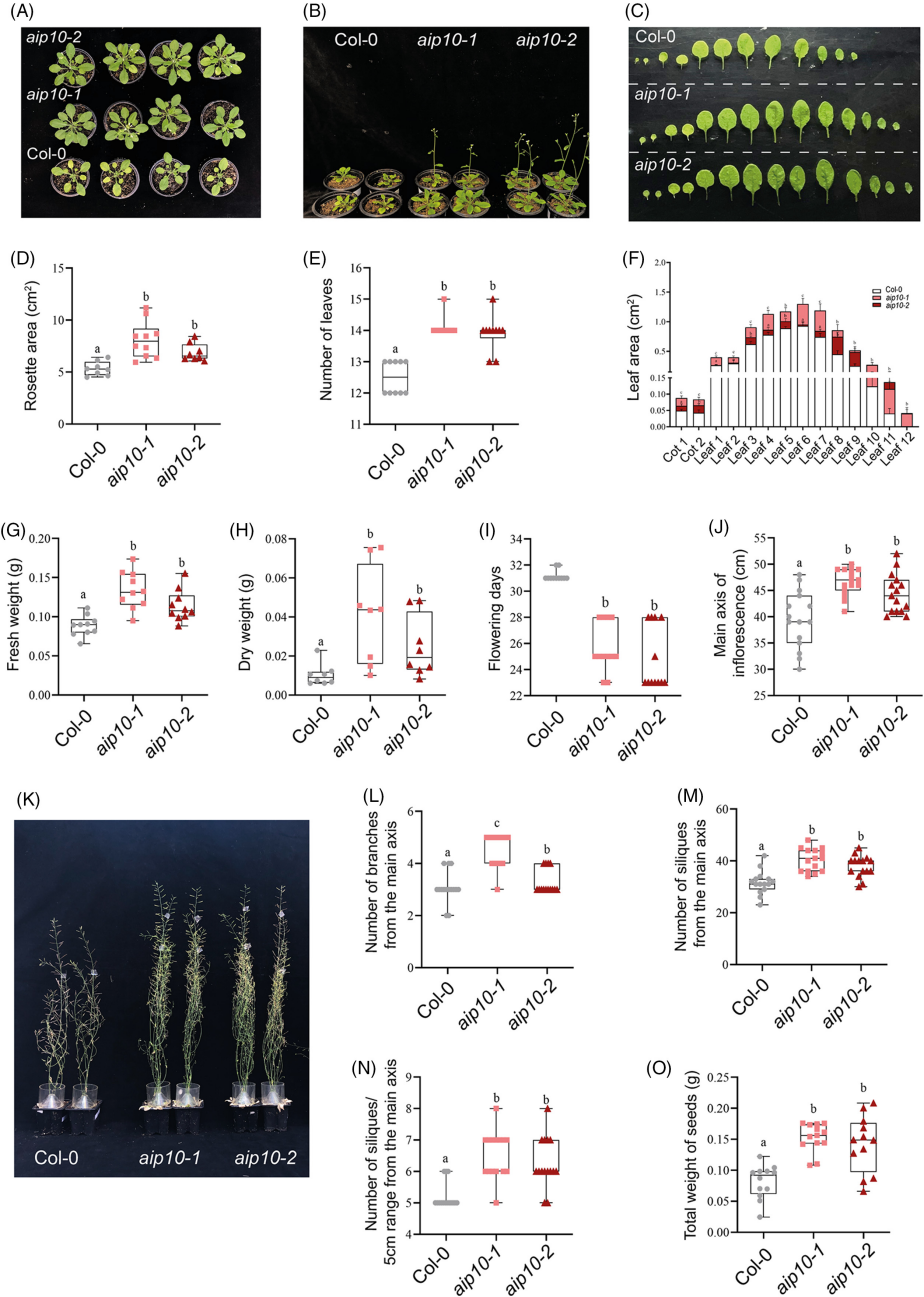
Phenotypic analyses of AIP10 knock‐out (*aip10‐1*) and knock‐down (*aip10‐2*) Arabidopsis plants compared to wild‐type Col‐0. Experiments carried out, in a photoperiod of 16 h/8 h at 21°C, cultivated directly in the soil. Representative images of (A) wild‐type Col‐0, *aip10‐1* and *aip10‐2* plants at 30 DAG and (B) 35 DAG. (C) Analysis of the leaf series of plants at 20 DAG to assess leaf area. (D) Rosette area measurement using ImageJ program. (E) Number of leaves at 32 DAG. (F) Measurement of leaf area at 22 DAG using ImageJ software. (G) Whole plant fresh weight and (H) Whole plant dry weight at 32 DAG. (I) Days to flowering. (J) Height of the main inflorescence axis at 57 DAG. (K) Representative images of Col‐0, *aip10‐1* and *aip10‐2* plants at 57 DAG during the reproductive stage. (L) Number of branches on the main inflorescence axis at 40 DAG. (M) Total number of siliques in the main inflorescence axis at 57 DAG. (N) Measurement of the number of siliques at a fixed interval of 5 cm on the main inflorescence axis at 57 DAG. (O) Total seed weight per individual. The box plot graphs show the distribution of data of 10 individuals for biomass and 15 individuals for reproductive analyzes. The box represents the interquartile range (IQR), with the inner line running down the median. The whiskers extend to the maximum and minimum values, including all points within that range. The difference between groups in all analysis was confirmed by one‐way anova (*P* < 0.05), and different letters indicate statistically different means according to Tukey's test at 5% probability. DAG, days after germination.

At the vegetative state, *aip10‐1 and aip10‐2* plants showed a larger rosette area compared to Col‐0 (Figure 3A,B,D; Figure S7), formed by larger leaves, in significantly higher numbers (Figure 3E). This was clearly demonstrated in leaf series analysis (Figure 3C,F). Increased growth in the *aip10* mutants led to a significant increase in fresh and dry weight (Figure 3G,H). Plants with reduced levels of *AIP10* showed an earlier transition from the vegetative to the reproductive stage when compared to Col‐0 plants (Figure 3B,I; Figure S7) under long day (LD) conditions (16 h light/8 h dark). This phenotype was more exacerbated in *aip10‐2* plants (Figure 3B; Figure S7), which corroborates observations in the *hdr1* mutant (AIP10 homolog in rice) that flowered 30 days before Wt under natural and long‐day conditions (Sun et al., 2016).

At the reproductive stage, *aip10* mutants showed a higher main inflorescence axis at the silique ripening stage (57 DAG), with a 16% and 12% significant increase compared to Col‐0, respectively (Figure 3J,K; Figure S7). *aip10* mutant plants grew and generated the reproductive organs with at least 20% more ramifications and siliques of the main inflorescence axis than Col‐0 (Figure 3L,N) and produced at least 30% more seeds compared to control plants (Figure 3O). Furthermore, *aip10* plants showed a delay in senescence, suggesting that meristems remained active for longer periods, contributing to the improved plant growth and production of plant organs.

Altogether, the gene expression and mutant phenotype analyses suggested that AIP10 acts throughout development, as it is present in all organs and meristems, and knockout of its expression leads to growth promotion. Next, we investigated the molecular and biochemical mechanisms by which downregulation of AIP10 promotes plant development.

### AIP10 is a negative modulator of cell divisions that influences the expression of ABAP1 target genes

**Figure 4:**
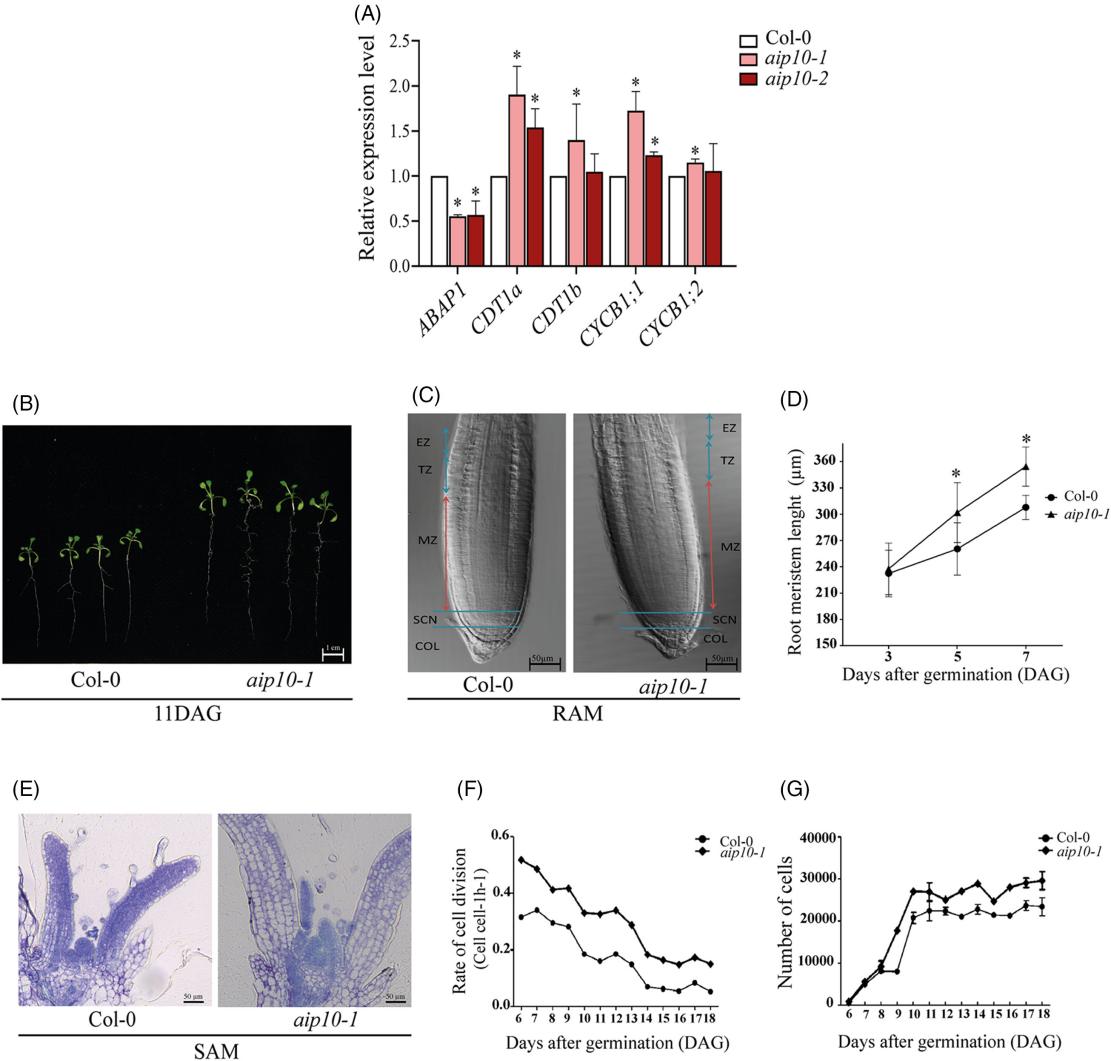
Cell division analyzes in *aip10‐1*, *aip10‐2* and wild‐type Col‐0 plants. (A) Expression analysis of *ABAP1*, *CyclinB1;1*, *CyclinB1;2*, and the ABAP1 target genes, *CDT1A* and *CDT1B*, in Col‐0, *aip10‐1*, and *aip10‐2* plants. Relative mRNA levels were evaluated in both genotypes in shoot of 11DAG seedlings grown *in vitro* in MS medium with 1% sucrose in a photoperiod of 12 h/12 h to 21°C. Each biological replicate (*n* = 3) consisted of a set of 10 plants. Data were normalized with the expression of *UBI14* and *GAPDH* as reference genes. Bars indicate standard deviation of biological replicates. Statistical analysis was performed using the Student's *t*‐test (*P* ≤ 0.05). Asterisks (*) indicate significant differences between samples relative to Col‐0. (B) Seedlings at 11 DAG, grown *in vitro* in 1% sucrose MS medium. (C) Bright‐field differential interference contrast of root apical meristem of *aip10‐1* and Col‐0 plants, 5 DAG (*n* = 5). COL, columella; EZ, elongation zone; MZ, meristematic zone; SCN, stem cell niche; TZ, transition zone. The arrows show the extension of meristems, 20× magnification. (D) Mean RAM length of plants at 3, 5, and 7 DAG (*n* = 10) grown *in vitro* in MS medium with 1% sucrose in a photoperiod of 12 h/12 h to 21°C. (E) Longitudinal section of the SAM of Col‐0 and *aip10‐1* plants at 10 DAG. Bars: 0.12 mm. All images are representative of the replicates analyzed. (F, G) Kinematic analysis of growth of the first leaf pair of Arabidopsis Col‐0 and *aip10‐1* plants, from 6 to 18 DAG (*n* = 5); (F) rate of cell division and (G) number of cells. The line graph corresponds to the monitoring of individuals from 6 to 18 days. Error bars represent averages ± SD of five plants.

As AIP10 interacts with ABAP1, a regulator of cell division in plants, and *aip10* mutants showed increased growth, the transcriptional regulation of two direct targets of ABAP1 repression at the G1/S transition, *CDT1a* and *CDT1b* genes, was investigated by RT‐qPCR. Remarkably, in *aip10‐1* plants, *ABAP1* expression was 50% lower in 11 DAG plants. Accordingly, *CDT1a* and *CDT1b* were increased by 90% and 20%, respectively, at 11 DAG (Figure 4A,B; Figure S8). These data suggest that AIP10 could act in a complex with ABAP1, repressing gene expression. Also, the AIP10‐ABAP1 interaction could operate in a feedback mechanism in which AIP10 could transcriptionally modulate *ABAP1* mRNA levels and thus its targets. In addition, *CYCB1;1 and CYCB1;2*, marker genes for cell division, showed around 70% and 15% higher expression, respectively, in the knockout plants at 11 DAG (Figure 4A; Figure S8), endorsing the higher rates of cell division observed in *aip10‐1*. In *aip10‐2*, the expression profile in 11 DAG leaves was similar to that observed in *aip10‐1* plants, except for *CDT1b* and *CYCB1;2*, which did not show significant higher expression in the mutant (Figure 4A; Figure S8). As *aip10‐1* growth phenotypes were more pronounced compared to *aip10‐2*, we can speculate there might be a minimum threshold level of reduction in *AIP10* expression to activate all the mechanisms by which AIP10 knockout improves plant growth. In roots, the expression of marker genes exhibited the same pattern observed in shoots in Col‐0 and *aip10‐1* (Figure S9a).

Next, we evaluated whether AIP10 could act together with ABAP1, modulating meristematic activities through assays using *aip10‐1* plants. Anatomical analysis showed increased length of the main root and a greater number of lateral roots (Figure 4B; Figure S9b,c), and longer Root Meristems (RAM) (Figure 4C,D) in *aip10‐1* plants compared to Col‐0. In the shoot apical meristem (SAM), the first and second pairs of leaves emerged earlier in *aip10‐1* plants compared to Col‐0 (Figure 4E). These data were corroborated by a kinematic assay of leaf development showing that higher rates of cell division were maintained for a longer period in *aip10‐1* (Figure 4F), leading to larger leaves (Figure S9d) with higher cell numbers (Figure 4G), while cell sizes were the same in *aip10‐1* and Col‐0 (Figure S9e). The data indicate that the improved early development of *aip10‐1* plants resulted from the boost in cell division.

Histochemical analysis illustrated an increased staining in SAM and RAM of *pCDT1a::GUS* x *aip10‐1* plants, compared to *pCDT1a::GUS* x Col‐0 (control), suggesting a more active *CDT1* promoter (Figure S10a,b). Also, DNA ploidy levels during vegetative development were assessed by flow cytometry. In both leaf and root, no significant differences in ploidy were observed between Col‐0 and *aip10‐1* (Figure S11a–c), which demonstrates that there was no difference in the endoreduplication process in the mutant.

### *AIP10* knockout triggers a major transcriptional reprogramming in pathways with impact on plant primary metabolism and on the response to the environment

To further investigate genetic pathways that could be involved in AIP10's role in development, transcriptomes were generated with 11 and 35 DAG samples of roots and shoots of Col‐0 and *aip10‐1* plants that presented the most pronounced growth phenotypes. The selected time points reflected two important stages to investigate the increase in size and productivity of *aip10‐1* plants. Differentially expressed genes (DEGs) identified in selected pathways were further investigated by RT‐qPCR in both mutants.

**Figure 5:**
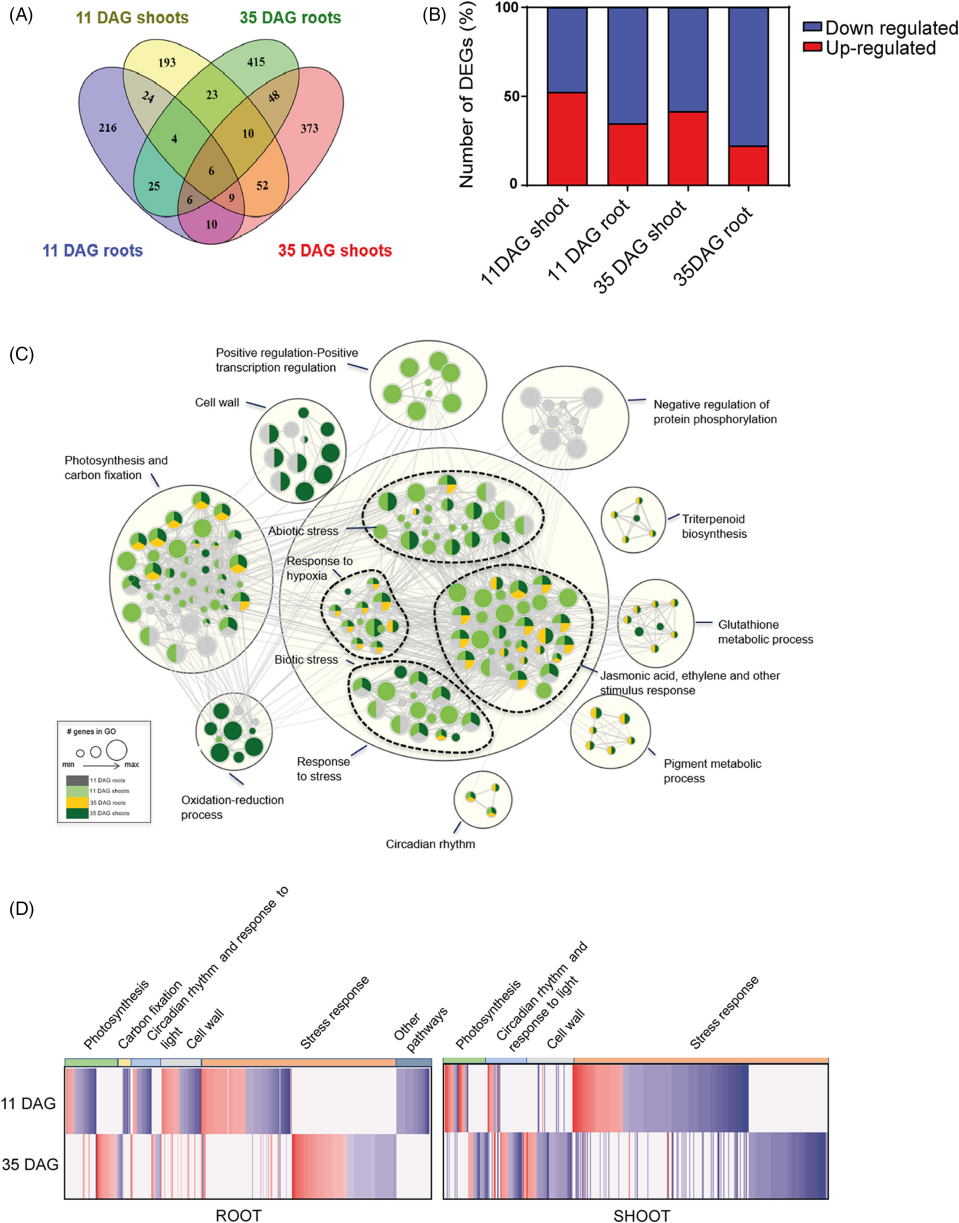
Gene expression reprogramming in the absence of *AIP10*. (A) Venn diagram showing the total number of differentially expressed genes (DEGs) for each transcriptome comparison, considering *aip10‐1* and Col‐0 in shoot and root tissues at 11 and 35 DAG from the website https://bioinformatics.psb.ugent.be/webtools/Venn/. (B) Percentage of the number of DEGs upregulated and downregulated in each comparison: *aip10‐1* and Col‐0 in shoot and root tissues at 11 DAG and 35 DAG. Upregulated DEGs are shown in red and downregulated are shown in blue. (C) Enrichment map (cytoscape enrichment map) displaying enriched pathways for each timepoint (11 DAG roots, gray; 11 DAG shoots, pale green; 35 DAG roots, yellow; 35 DAG shoots, dark green), regrouped in clusters. Nodes represent gene enrichment sets. Node size corresponds to the number of genes. Edges join gene sets that share common genes. (D) Heatmap with main clusters for comparisons between *aip10‐1* and Col‐0 at 11 DAG and 35 DAG, in roots (left panel) and shoots (right panel). Colors represent log_2_‐fold change (violet: downregulated and red: upregulated).

Illumina sequencing of eight libraries at 11 DAG yielded a total of 264.3 million reads, and sequencing of 12 libraries at 35 DAG yielded approximately 1,779 million reads. Differential expression between *aip10‐1* and Col‐0 was compared, identifying 300 DEGs for 11 DAG roots (157 up/143 down), 323 DEGs for 11 DAG shoots (112 up/211 down), 537 DEGs for 35 DAG roots (223 up/314 down), and 514 DEGs for 35 DAG shoots (114 up/400 down) (Figure 5A,B).

Gene‐ranked pathway enrichment analysis with g:Profiler was performed to identify enriched functional categories of the differentially expressed genes (Figure 5C). The Cytoscape map exhibits nodes that correspond to enrichment terms colored depending on their presence on each *aip10‐1* transcriptome timepoint and tissue (11/35 DAG roots/shoots), while edges correspond to shared genes between pathways. Nodes were regrouped into larger clusters to identify major categories of enriched pathways. Photosynthesis and carbon fixation were the second major cluster, followed by cell wall, circadian rhythm, and light response. Some clusters were only associated with one point in time, such as the induction of transcription factors in 11 DAG root and the repression of protein phosphorylation in 11 DAG shoot. Furthermore, enriched oxidation–reduction process pathways were mainly present in 35 DAG shoot, and triterpenoid biosynthesis, glutathione metabolic process, and pigment metabolic process were DEGs only in 35 DAG (Figure 5C).

Heat maps for the most important clusters were generated and notable patterns were identified (Figure 5D). Remarkably, the sets of genes linked to clusters, such as photosynthesis, carbon fixation, circadian rhythm, response to light, cell wall, and stress response, were distinct between 11 and 35 DAG, both in roots and shoots, suggesting that *aip10‐1* plants differentially remodeled their transcriptional program between the two time points. Although the absence of *AIP10* may modulate the transcriptional program, developmental differences caused by the mutation, which accelerates development relative to Col‐0, may also contribute to some of the observed transcriptome variations.

### Transcriptional reprogramming in *AIP10* silenced plants might involve the SnRK1 regulatory pathway

**Figure 6:**
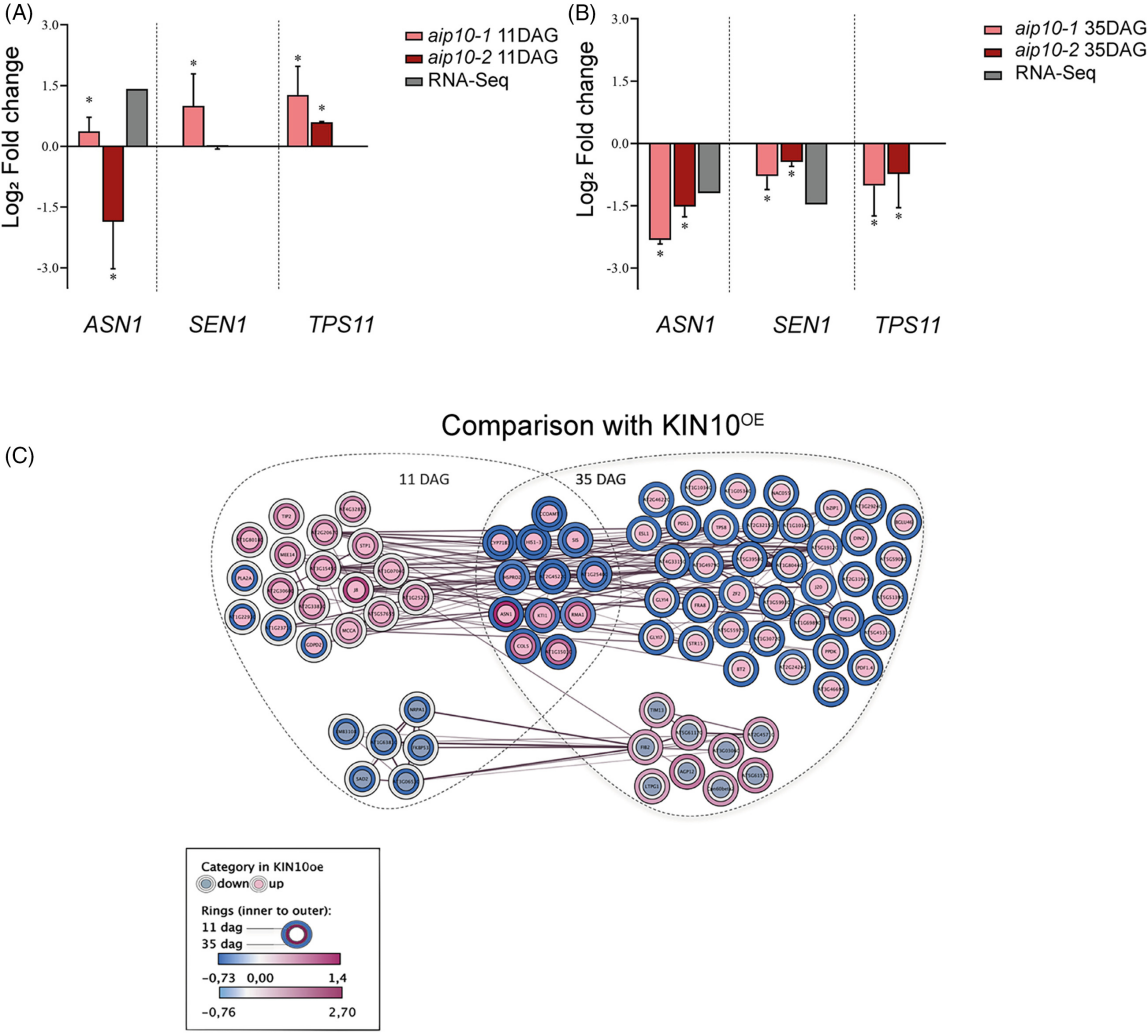
Expression profile analysis of KIN10 target genes in *aip10‐1* transcriptomes to indirectly assess SNRK1 activity. (A, B) Validation by RT‐qPCR of selected SnRK1 (KIN10oe) marker genes differentially expressed (DEGs) in *aip10‐1* shoot transcriptomes. Relative mRNA levels of *ASN1*, *SEN1*, and *TPS11* genes in Col‐0, *aip10‐1*, and *aip10‐2* plants were evaluated in both genotypes, in (A) shoot of 11 DAG seedlings grown *in vitro* in MS medium with 1% sucrose in a photoperiod of 12 h/12 h to 21°C and (B) shoot of 35 DAG plants grown directly in the soil in a photoperiod of 16 h/8 h to 21°C. Expression levels were normalized by the mRNA levels of the *UBI14* and *GAPDH* genes. Bars represent means ± SD (standard deviation) and * significantly different from Col‐0 with *P* ≤ 0.05 with Student's *t*‐test. Each biological replicate (*n* = 3) consisted of a set of 10 plants at 11 DAG and 5 plants at 35 DAG. The graph bars indicate each biological replicate in log_2_ fold change data, where value <0 are repressed and >0 are induced. (C) Comparative expression profile analysis of genes (circles) modulated in both KIN10oe/sugar deprivation and *aip10‐1* transcriptomes at 11 DAG and/or 35 DAG. Color of inner circles in the map (Cytoscape Omics Visualizer) indicates the expression profile of DEGs in KIN10oe (green: downregulated, magenta: upregulated). An expression heat map for DEGs in *aip10‐1* is represented by colors in rings around the inner circle, indicating the log_2_ fold change (inner ring: 11 DAG and outer ring: 35 DAG). Edges join genes related to a common network or pathway. DAG, days after germination.

The functional categories of DEGs in *aip10‐1* transcriptomes showed an enrichment for clusters related to plant metabolism. As AIP10 can interact with KIN10 (Figure 1D,E), a subunit of SnRK1, we investigated the involvement of this kinase in the transcriptional reprogramming profiles observed in *aip10 mutants*. First, we analyzed the gene expression profile in both mutants of known marker genes of SnRK1: *ASN1* (*asparagine synthetase* 1), *SEN1* (*senescence‐associated 1*), and *TPS11* (*trehalose phosphate synthase 11*). Based on the downregulated profile of SnRK1 target genes at 35 DAG, at the transition to the reproductive phase when compared to Col‐0 (Figure 6B), the data suggest that SnRK1 was less active in both mutants at this timepoint. On the other hand, the upregulated profile of the target genes at 11 DAG, at earlier stages of development, suggests that SnRK1 was more active in *aip10‐1* plants; however, the expression profile was not so clear in *aip10‐2* plants (Figure 6A).

We further investigated SnRK1 associated transcriptional responses in *aip10‐1* plants, comparing the transcriptome profile of genes regulated by KIN10 overexpression (KIN10oe) and sugar starvation (Baena‐González et al., 2007) with the profiles in 11 DAG and 35 DAG *aip10‐1* transcriptomes. SnRK1 activity is increased during sugar starvation due to low levels of trehalose‐6‐phosphate (T6P), which is used as a signal to indicate a homeostatic sugar imbalance in plants (Baena‐González & Lunn, 2020). Figure 6(C) presents a Map (Cytoscape Omics Visualizer) showing common DEGs between *aip10‐1* and KIN10oe transcriptomes. Nineteen genes that were upregulated, and six genes that were downregulated by KIN10oe/sugar starvation, presented a similar differential expression pattern in *aip10‐1* at 11DAG, suggesting increased SnRK1 activity at this developmental stage. Contrastingly, at 35 DAG, all 51 genes that were upregulated and nine genes that were downregulated by KIN10oe/sugar starvation showed an opposite expression pattern in *aip10‐1* plants, suggesting that SnRK1 was less active.

SnRK1 and TOR have been shown to exert their roles through large transcriptional reprogramming which affects modulation of nutrient‐driven processes mostly in opposite ways (Baena‐González et al., 2007; Dong et al., 2015; Forzani et al., 2019). Therefore, other comparisons were performed between the expression profiles in transcriptomes that were generated: (i) in conditions when SnRK1 is active (KINoe/sugar starvation); (ii) *lst8* mutant, which compromises a subunit essential for the stability and full activity of TOR kinase, represents a condition of partial inhibition of the TOR pathway in plants (Forzani et al., 2019); (iii) during inhibition of TOR (iTOR) activity in 11 DAG seedlings treated for 24 h with 2 μm AZD8055, a potent inhibitor of plant growth (Dong et al., 2015); (iv) when AIP10 is silenced, at two developmental stages (Figure S12).

A large number of genes identified as SnRK1 targets (Baena‐González et al., 2007) were also regulated under mild TOR inhibition conditions (*lst8*, Forzani et al., 2019) (Figure S12), and some of these genes were also regulated in *aip10‐1*. However, at 35 DAG, the gene expression patterns in *aip10‐1* contrasted with those observed in KIN10oe and *lst8*, which are associated with growth inhibition. In *aip10‐1*, the distinct transcriptional profile of these two models suggests that the mutation promotes a favoring of plant development, since it presents the opposite profile.

Comparative analyses between DEGs from the *aip10‐1* transcriptomes and iTOR, which refers to a stronger inhibition of TOR activity, showed that, among the set of genes upregulated by iTOR, many genes were common to *aip10‐1* (Figure S13). Most DEGs were downregulated in our 35 DAG mutant, suggesting that TOR is activated at this time. Additionally, when comparing DEGs from the *aip10‐1* transcriptomes, mild TOR inhibition in the *lst8* mutant (Forzani et al., 2019), and strong TOR inhibition with AZD8055 (iTOR) (Figure S14) revealed that the transcriptional response observed when comparing *aip10‐1* with *lst8* or iTOR was similar, regardless of the intensity of TOR inhibition. The expression profile of DEGs in *aip10‐1* was opposite to those observed under mild and strong TOR inhibition conditions, suggesting that when TOR is inhibited – either mildly or strongly – the gene expression profile diverges from the pattern observed in *aip10‐1*.

From the transcriptional data we can speculate that SnRK1 was possibly more active in early stages of development in parallel with TOR in *aip10‐1* at 11 DAG, while TOR appeared to predominate in the transition to the reproductive stage in the mutant when compared to the control. However, further functional experiments would be necessary to confirm the involvement of the TOR pathway in *aip10‐1* plants.

### *AIP10* knockout increases the photosynthesis and CO_2_ assimilation efficiencies

Next, we further investigated the primary metabolism reprogramming modulated by AIP10 knockout by combining the phenotyping and transcriptome data with biochemical and metabolic analyses.

**Figure 7:**
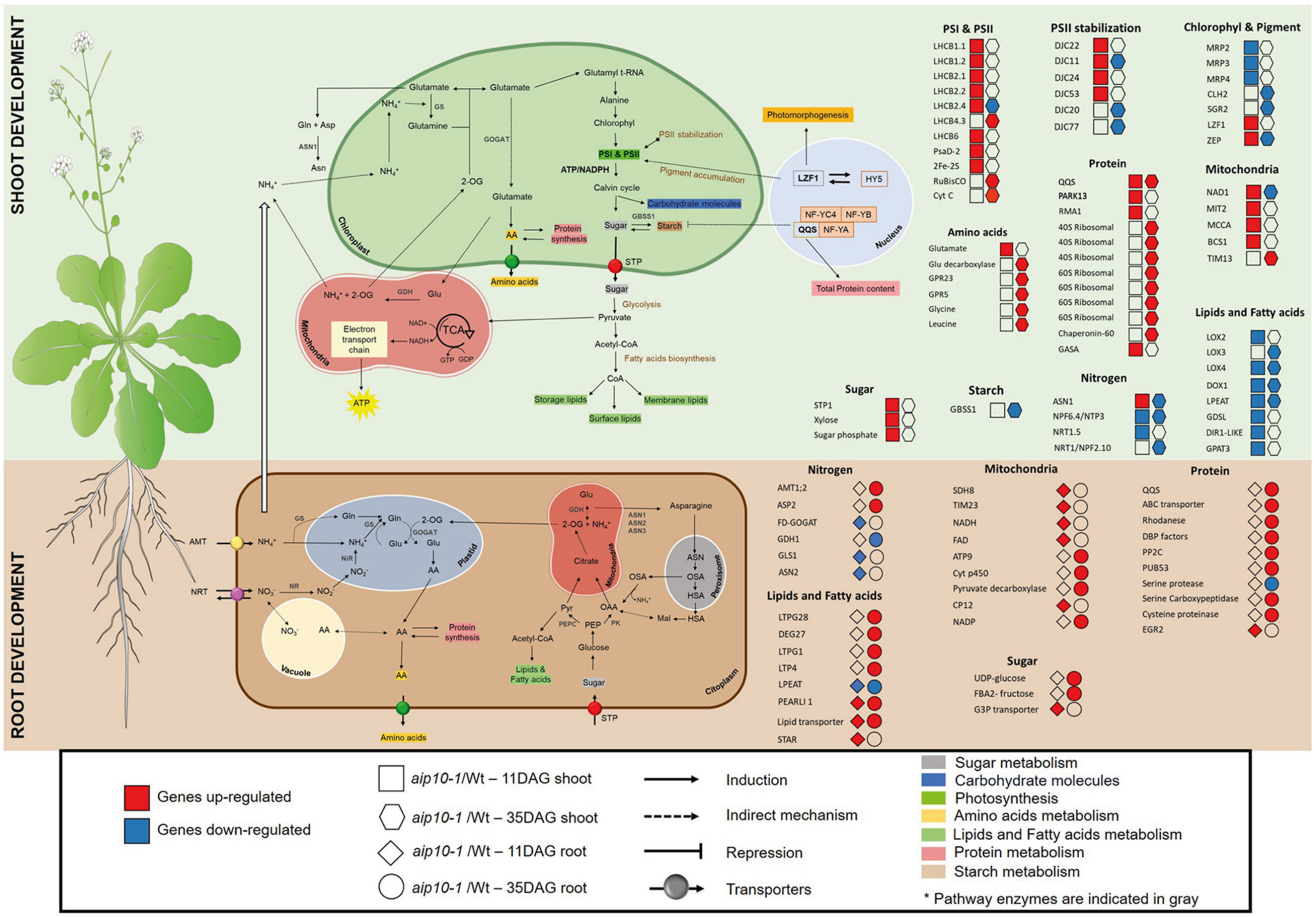
Main genes involved in energy metabolism differentially expressed in shoots and roots of *aip10‐1* plants. The schematic representation depicts the expression pattern of genes involved in CO_2_ uptake and carbon metabolism, photosynthetic pigment synthesis and degradation, as well as genes related to nitrogen, protein, amino acids, starch and sucrose metabolism in both leaves and roots of *aip10‐1* plants. The green background refers to the DEGs of the shoot and the brown refers to the DEGs of the root. DEGs in different transcriptomes are represented as follows: squares for 11 DAG shoot, hexagons for 35 DAG shoot, diamond for 11 DAG root and the circle for 35 DAG root. Red color refers to upregulated genes and blue color refers to downregulated genes. The icons present in the figure were highlighted in the footer of the figure. The main products of each route were highlighted with different colors as indicated in the figure. Gray: sugar metabolism; blue: carbohydrate molecules; fluorescent green: photosynthesis; yellow: amino acid metabolism; light green: lipid and fatty acid metabolism; pink: proteins; brown: starch. DAG, days after germination; DEGs, differentially expressed genes.

**Figure 8:**
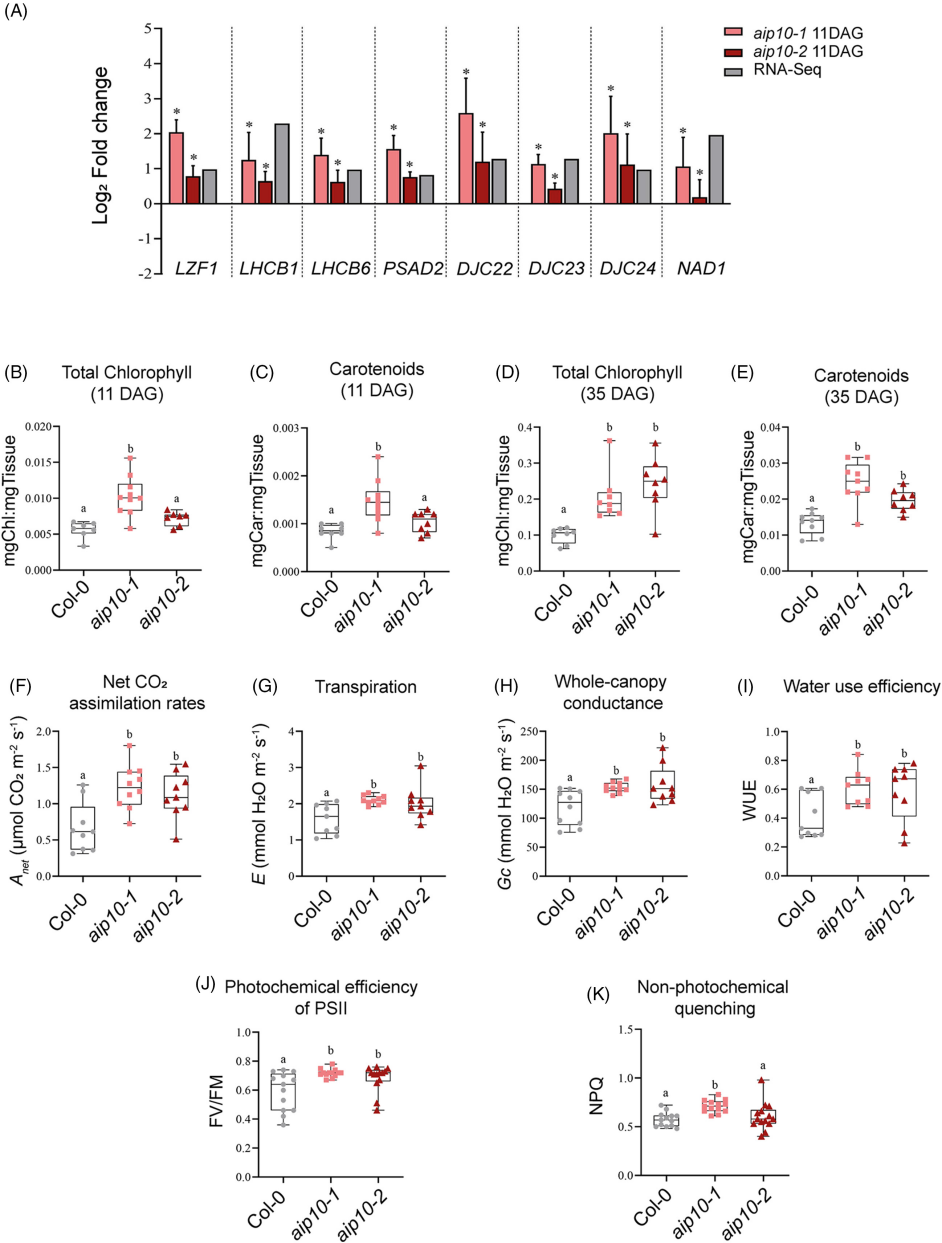
Analysis of photosynthetic genes, chlorophyll content and photosynthesis parameters in *aip10‐1* and *aip10‐2* plants. (A) Relative mRNA levels of genes related to photosynthesis (*LZF1*, *LHCB1*, *LHCB6*, *PSAD2*, *DJC22*, *DJC23*, *DJC24*, and *NAD1*) in shoot of 11 DAG seedlings of Col‐0, *aip10‐1*, and *aip10‐2* plants grown *in vitro* in MS medium with 1% sucrose in a photoperiod of 12 h/12 h to 21°C. Expression levels were normalized by the mRNA levels of the *UBI14* and *GAPDH* genes. Bars represent means ± SD (standard deviation) and * significantly different from Col‐0 with *P* ≤ 0.05 with Student's *t*‐test. Each biological replicate (*n* = 3) consisted of a set of 10 plants at 11 DAG. RT‐qPCR and RNA‐Seq data are presented. The graph bars indicate each biological replicate in log_2_ fold change data, where value <0 are repressed and >0 are induced. (B–K) Evaluation of photosynthetic physiological parameters. (B, D) Total chlorophyll (*a*/*b*) and (C, E) carotenoid content of Col‐0, *aip10‐1* and *aip10‐2* plants grown directly in the soil in a photoperiod of 16 h/8 h to 21°C at (B, C) 11 DAG and (D, E) 35DAG (*n* = 10). (F) Net CO_2_ assimilation rate; (G) transpiration rate; (H) whole‐canopy conductance. (I) water use efficiency (WUE); measurements were performed with the LICOR‐6400 XT Instrument at 22°C in ambient CO_2_ at 400 μmol photons m^−2^ sec^−1^ (*n* = 10). (J, K) Chlorophyll fluorescence parameters of Col‐0, *aip10‐1* and *aip10‐2* plants at 11 DAG, including: (J) photochemical efficiency of PSII under dark adaptation (Fv/Fm), (K) non‐photochemical quenching (NPQ); measurements were performed with the Fluorcam 800 MF, Photon Systems Instruments (*n* = 15). The box plot graphs show the distribution of data. The box represents the interquartile range (IQR), with the inner line running down the median. The whiskers extend to the maximum and minimum values, including all points within that range. The difference between groups in all analysis was confirmed by One‐Way anova (*P* < 0.05), and different letters indicate statistically different means according to Tukey's test at 5% probability.

Photosynthesis belongs to the second largest cluster of pathways enriched in *aip10‐1* plants (Figure 5C). Most DEGs related to photosynthesis in 11 DAG shoot of *aip10‐1* were induced (Figure 7), such as genes from Photosystems I and II (PSI and PSII), including seven *light‐harvesting chlorophyll a/b binding* (*LHCB*) and *PsaD2*. These LHCB proteins serve as the Photosystem II antenna complex (Jansson, 1999) and PsaD2 is a component of the PSI reaction center (Ihnatowicz et al., 2004). Three genes from the chaperone domain *DnaJ* (DJ) superfamily, involved in the stabilization of PSII (Chiu et al., 2013), and *LZF1*, described as a transcriptional regulator that influences chloroplast biogenesis and function (Chang et al., 2008), were also induced. Furthermore, *NDA1*, a type II NAD(P)H dehydrogenase, was also induced and may have a role in stabilization and redox regulation in the mitochondrial respiratory chain (Wallström et al., 2014). These genes were analyzed through RT‐qPCR in the mutants, validating their expression profile in plants with total and partial loss of *AIP10* function (Figure 8A).

Differently from what was observed at 11 DAG, *aip10‐1* plants at 35 DAG showed mainly repressed photosynthesis‐related DEGs. This difference is possibly due to the formation of the photosynthetic apparatus, which still occurs at 11 DAG, a period in which most genes are being modulated. Together, these data suggest that silencing *AIP10* appears to improve the formation of the photosynthetic apparatus in the early stages of development through reprogramming gene expression.

Consistent with RNA‐seq data, *AIP10* knockout affected photosynthetic traits, showing a higher content of chlorophyll and carotenoids at 11 and 35 DAG in both mutants (Figure 8B–E) when compared to Col‐0. Under greenhouse conditions, *aip10‐1* and *aip10‐2* at 25 DAG showed rates of up to 83% higher CO_2_ uptake (*Anet*) (Figure 8F) and a higher transpiration rate (*E*) (Figure 8G) which remained relatively lower than the gain obtained from photosynthesis. Added to this, *aip10* mutants showed higher stomatal conductance (*g*_c_) (Figure 8H) and water use efficiency (WUE) (Figure 8I), indicating that these plants capture more CO_2_ and lose fewer water molecules at each stomatal opening. The measurement of photosystem II efficiency (Fv/Fm) showed that *aip10* mutants had greater photosystem II activity (Figure 8J) in addition to greater energy dissipation in the form of heat (Figure 8K).

### *AIP10* knockout modulates the flow of carbon fixation into biomass by improving contents of protein, lipids (triglycerides) and carbohydrates

**Figure 9:**
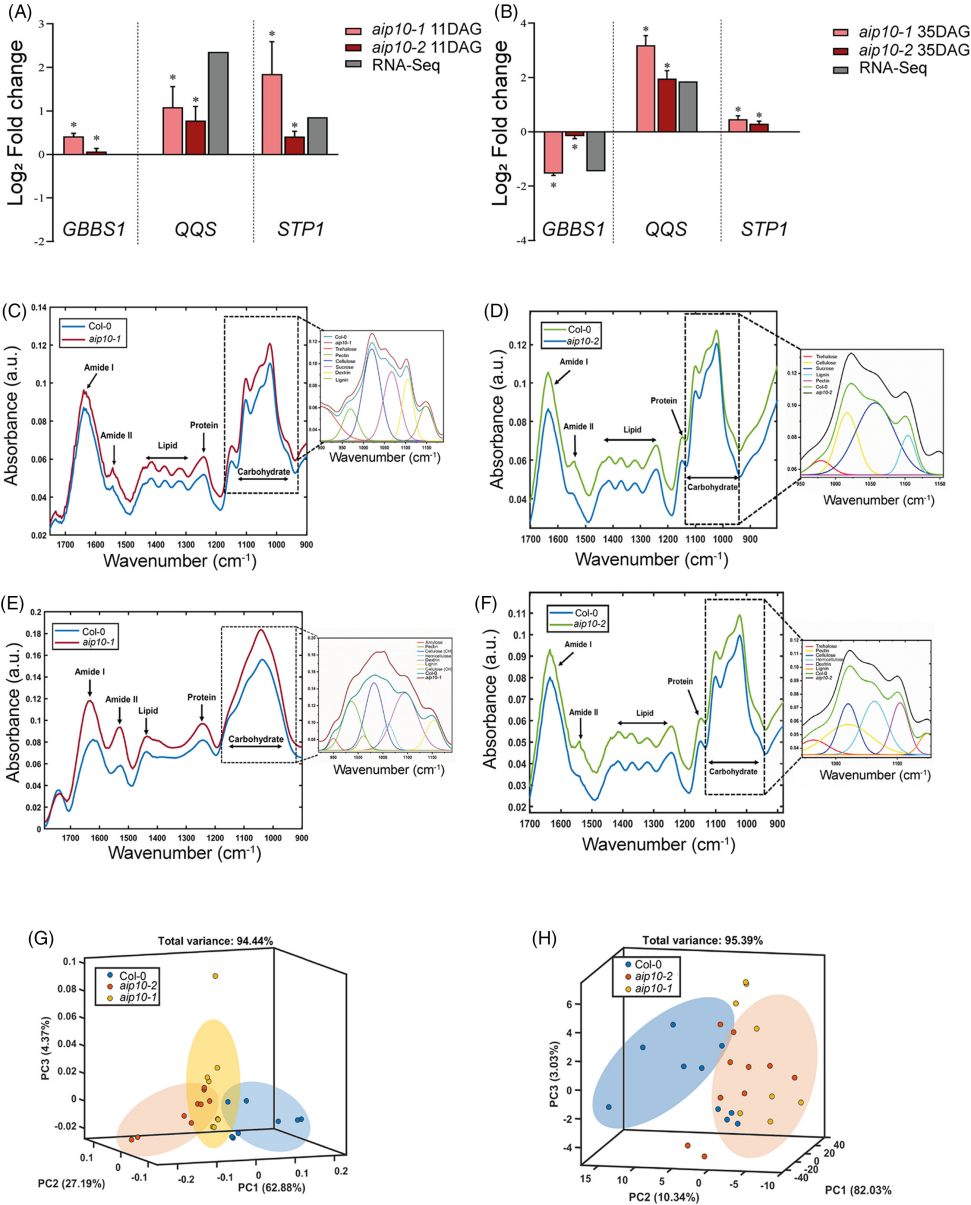
Metabolic profiles of *aip10‐1* and *aip10‐2* plants. (A, B) Relative mRNA levels of genes involved in carbon metabolism, differentially expressed in the *aip10‐1* transcriptome (*GBSS1*, *QQS*, and *STP1* genes), in Col‐0, *aip10‐1*, and *aip10‐2* plants. Expression levels were evaluated in both genotypes in (A) shoot of 11 DAG seedlings grown *in vitro* in MS medium with 1% sucrose in a 12 h/12 h photoperiod at 21°C and (B) shoot of 35 DAG plants cultivated directly in soil in a photoperiod of 16 h/8 h 21°C. Expression levels were normalized by the mRNA levels of the *UBI14* and *GAPDH* genes. Bars represent means ± SD (standard deviation) and * significantly different from Col‐0 with *P* ≤ 0.05 with Student's *t*‐test. Each biological replicate (*n* = 3) consisted of a set of 10 plants at 11 DAG and 5 plants at 35 DAG. RT‐qPCR and RNAseq data are presented. The graph bars indicate each biological replicate in the log_2_ fold change data, where value <0 are repressed and >0 are induced. (C–F) Metabolic profiling of 35 DAG leaves and mature seeds from Col‐0, *aip10‐1* and *aip10‐2* plants grown directly in soil in a photoperiod of 16 h/8 h 21°C, by using a Shimadzu IRPrestige‐21 attenuated total reflectance infrared spectrometer (ATR‐FTIR). Data show average spectra obtained by the ATR‐FTIR technique in the region of 900–1700 cm^−1^ which correspond to the regions of most sensitive absorption of the main components. (C) 35 DAG leaves of Col‐0 and *aip10‐1* plants. (D) 35 DAG leaves of Col‐0 and *aip10‐2* plants. The experiment was carried out on two different leaves from four individuals of each genotype. The assigned values are normalized by the area of the diamond crystal plate. (E) Mature seeds obtained from Col‐0 and *aip10‐1* plants. (F) Mature seeds obtained from Col‐0 and *aip10‐2* plants. The experiment was carried out in three different macerated pools of 100 seeds each. The assigned values are normalized by the area of the diamond crystal plate. (G, H) Score plot for the three principal components applied to the seed ATR‐FTIR dataset of Col, *aip10‐1*, and *aip10‐2* plants respectively.

Carbon fixation belonged to the second major cluster of enriched pathways in *aip10‐1* plants (Figure 5C). We observed an enrichment of pathways such as sugar, protein, starch, nitrogen, and lipids (Figure 7). Among the DEGs induced in *aip10‐1* in11 DAG shoot was *STP1* (*sugar transporter protein 1*), which encodes an H+/monosaccharide cotransporter. At 35 DAG, ribosomal genes were induced, indicating continuous protein production throughout development. Some lipid‐related genes were mainly induced in 11 DAG root and repressed in 11 DAG and 35DAG shoot. Furthermore, *QQS* (*Qua‐Quine starch*), an *A. thaliana* orphan gene that acts as a regulator of starch biosynthesis, was upregulated in *aip10‐1* at both timepoints. Its overexpression in soybean triggered increased protein content and decreased starch levels (Li & Wurtele, 2015). Besides, there was repression of the *GBSS1* gene (g*ranule‐bound starch synthase 1*) involved in starch accumulation, encoding a protein from the UDP‐Glycosyltransferase superfamily that is responsible for the biosynthesis of amylose in plants. These data suggest that starch was redirected to the synthesis of other metabolites in *aip10‐1*. The gene expression profiles were validated through RT‐qPCR (Figure 9A,B) in the mutants. Consistent with the transcriptional profiles, biochemical analyses showed that both *aip10‐1* and *aip10‐2* mutants accumulated more soluble sugars and less starch in mature leaves than wild‐type plants (Figure S15). In siliques, *aip10‐1* maintained high sugar levels, while *aip10‐2* showed reduced sugar accumulation, suggesting differences in sugar allocation to sink tissues. These results reinforce that *AIP10* regulates sugar mobilization and storage to support enhanced cell division and growth.

To further explore the metabolic alterations in *aip10* mutants beyond sugar and starch content, we performed a broader biochemical analysis using ATR‐FTIR spectroscopy. This technique detects specific functional groups based on characteristic bond vibrations, enabling a global overview of metabolite composition (Talari et al., 2017) (Table S4). Within the acquisition range (500–4000 cm^−1^), a region called spectral bio‐fingerprint was established between 900 and 1800 cm^−1^ (Figures S16a,b and S17d), responsible for providing a large amount of information about the chemical groups present in biomolecules both in leaves and seeds (Santos et al., 2020). The most distinct band regions demonstrated that leaves of *aip10* mutants at 20 DAG (Figure S17a,b) and mature leaves at 35 DAG (Figure 9C,D) showed approximately 19% higher abundance of proteins compared to Col‐0 plants. The mutants presented higher content of lipids and carbohydrates at 20 DAG (Figure S17a,b) and 35 DAG (Figure 9C,D). The increased metabolic content in the leaves of the *aip10* mutants might be contributing to their greater biomass. Seeds of mutants also presented higher metabolic contents (Figure 9E,F), showing a gain of approximately 40% in proteins, 14% in lipids, and 18% in carbohydrates (triglycerides). The mutants presented higher levels of reserve polysaccharides. The PC model demonstrated that the mutants cluster together, showing their metabolic similarity in both tissues that differ from Col‐0 (Figure 9G,H; Figure S17c).

## DISCUSSION

Plant development is shaped by a series of environmental factors, driving adaptive forms for their survival. In this work, we investigated a protein belonging to the ABAP1 regulatory pathway, called AIP10, which integrates the modulation of the plant's cell cycle and primary metabolism. Plants with total and partial loss of AIP10 function activated cell divisions, leading to organs with increased cell numbers and biomass that are possibly supported by the transcriptional reprogramming that improved primary metabolism, photosynthesis, and carbon fixation.

### AIP10 is a member of the ABAP1 regulatory network that interacts with ABAP1 and KIN10, a subunit of SnRK1 kinase

Plant growth is the result of cell division rates and also the balance between cell proliferation, expansion, and differentiation (Jones et al., 2017). ABAP1 and the proteins with which it interacts (AIPs) constitute a plant‐specific pathway for modulating plant cell cycle progression at the G1/S transition, an important phase modulated by environmental signals (Masuda et al., 2008). ABAP1 contains eight predicted repeats of the Armadillo β‐catenin (ARM) domain in its N‐terminal portion, critical for protein–protein interactions (Zhou et al., 2015), and a BTB/POZ (Broad complex/Tram‐Track/Bric‐a‐brac/Poxyvirus and zinc finger) in its C‐terminus. Due to the diverse repeats of the ARM domain, ABAP1 can associate with different proteins, allowing multiple interactions to occur simultaneously to form different complexes (Masuda et al., 2008).

The characterization of different ABAP1 interactors provided evidence that ABAP1 performs different functions within the cell cycle. When interacting with TCP24, ABAP1 is capable of binding to the CDT1a/b promoters, repressing the expression of both genes (Masuda et al., 2008). The ABAP1 interactor (AIP1), which contains an Agenet/Tudor domain, also interacts with unmodified histones and with LHP1 (LIKE HETEROCHROMATIN PROTEIN 1), being involved in chromatin compaction remodeling and gene silencing that regulate flowering through FLT repression (Brasil et al., 2015). ABAP1 also modulates the differentiation of male and female gametophytes by forming a complex with AtTCP16 in male gametogenesis and with ADAP in female gametogenesis, which repress the transcription of its target genes, CDT1b and EDA24, respectively (Cabral et al., 2021).

Here we characterized an ABAP1 interactor, AIP10, and provided data on its dual role in cell divisions and in transcriptional reprogramming, through its protein interactions with ABAP1 and KIN10. Our data showed several pieces of evidence supporting a joint role of AIP10 and ABAP1 as negative modulators of proliferative cell divisions in all plant meristems. All four AIP10 isoforms were expressed throughout development, indicating a potential role in DNA replication and proliferative cell divisions in various plant meristems. AIP10 directly interacted with ABAP1, and they were found in a complex *in vivo*. AIP10, similarly to ABAP1, was exclusively localized in the nuclei, and both proteins were present in meristems in cycling cells, being absent in mitotic cells (Masuda et al., 2008). AIP10's role in cell division could occur through direct interaction with ABAP1, but also through a feedback mechanism of transcriptional control in which AIP10 could somehow transcriptionally modulate ABAP1 mRNA levels, since AIP10 silenced plants showed reduced ABAP1 mRNA levels. Accordingly, the mRNA levels of two direct targets of ABAP1 repression at the G1/S transition, *CDT1a* and *CDT1b* genes, were upregulated in AIP10 silenced plants. Finally, a joint role of AIP10 with ABAP1 could enable a negative modulation of the pre‐RC, repressing DNA replication and proliferative cell division. This hypothesis was supported by the kinematics data showing that AIP10 knockout increased rates of cell division and cell numbers in leaves; increased lateral root numbers in roots; and raised expression of cell division marker genes, both in shoots and roots.

We also showed that AIP10 physically interacted with KIN10, but not with KIN11, the catalytic subunits of SnRK1, a metabolic regulator involved in stress responses (Peixoto & Baena‐González, 2022). Homologs of AIP10 in bryophyte *P. patens* (SKI1 and SKI2) and rice (HDR1) also showed interaction with proteins homologous to the catalytic subunit of yeast Snf1 kinase and plant SnRK1 (Sun et al., 2016; Thelander et al., 2007). The AIP10.1, AIP10.2, and AIP10.3 isoforms of AIP10 have in the C‐terminal portion a nuclear localization site and a putative phosphorylation site initially identified in the two proteins that interact with SnRK1 homologs. In this same region, the AIP10.1 and AIP10.3 isoforms have a coiled‐coil conformation that has previously been implicated in interactions with SnRK2s in *Medicago truncatula* and is present in SnRK1a‐interacting negative regulators (SKINs) in rice (Lin et al., 2014; Nolan et al., 2006).

Furthermore, it was reported that AIP10 can interact with Val2 (VIVIPAROUS‐1/ABSCISIC ACID INSENSITIVE 3‐LIKE 2) (Altmann et al., 2020), a transcriptional repressor that recruits the Polycomb repressive complex 2 (PRC2) (Yuan et al., 2021) to catalyze H3 Lys27 trimethylation (H3K27me3) in some of its targets, regulating the expression of development and flowering genes (Gan et al., 2015). These data strengthen that AIP10 has a role in transcriptional modulation, whether at the genetic or epigenetic level. Remarkably, VAL2, as well as AIP1, were shown to interact with LHP1, to regulate flowering (Brasil et al., 2015; Yuan et al., 2021), suggesting a possible connection of these proteins and the ABAP1 network. Also, an epigenetic role of SnRK1 was shown in rice, as the kinase stimulates JMJ705, an H3K27me3 demethylase, to control energy homeostasis (Wang et al., 2021). Altogether, the data support the hypothesis that AIP10 acts in a transcriptional reprogramming hub, in specific developmental contexts.

### AIP10 is involved in integrating cell cycle, transcriptional reprogramming, and primary metabolism, modulating plant development

The absence of AIP10 promoted vegetative growth through increased cell divisions. In addition to the expansion of meristems, the greater biomass of knockout plants was a consequence of the production of more organs at an accelerated pace and for a longer period of time throughout the plant's life cycle. The cell cycle is an energetically consuming process dependent on the availability of energy metabolites. AIP10 interacts with SnRK1, a central metabolic regulator (Baena‐González et al., 2007; Baena‐González & Lunn, 2020) that acts in a complex network that includes TOR, a master regulator of development (Margalha et al., 2019).

Several genes modulated by the absence of AIP10 converged with the gene expression profiles regulated by SnRK1 and TOR pathways that share partially antagonistic regulatory activities (Margalha et al., 2019). Therefore, we can speculate that the transcriptional reprogramming observed in AIP10 silenced plants could be mediated, at least in part, by SnRK1 and TOR activities. The transcriptional profile data suggested that in the absence of AIP10, TOR was predominantly more active throughout development, thus favoring plant growth, in line with the *aip10‐1* phenotypes observed. Accordingly, SnRK1 seemed to be less active in *aip10‐1* and *aip10‐2* mature plants. However, in young *aip10‐1* seedlings, there was evidence of increased SnRK1 activity due to the induction of marker genes, which was not as explicit in *aip10‐2*. An increase in SnRK1 levels and activity at the beginning of germination has already been demonstrated (Lu et al., 2007). Even under favorable conditions, fluctuations in sugar levels during seedling establishment activate SnRK1 (Henninger et al., 2022) due to the need for greater energy supply and sugar homeostasis (Peixoto & Baena‐González, 2022). Thus, SnRK1 could modify developmental programs according to metabolic state, to adjust plant growth to a specific environment (Margalha et al., 2019). SnRK1 activates photosynthetic genes and adjusts growth in response to carbon (Zhang et al., 2009). Therefore, the expression profile of genes related to photosynthetic efficiency in AIP10 silenced plants could be mediated by SnRK1 activities. In parallel, the TOR signaling pathway can activate meristems and cell division by controlling E2Fa regulators, while its inhibition by SnRK1 hinders cell cycle progression (Sablowski, 2016). Thus, we can also speculate that activated TOR, in the absence of AIP10, could be contributing to the higher cell division rates in *aip10‐1*.

Plant cell division is highly dependent on energy status, with sugars being central products of photosynthesis and also crucial for metabolic signaling (Xiong & Sheen, 2015). Sugar availability activates the TOR pathway, promoting growth and biosynthesis (Dobrenel et al., 2013), while sugar scarcity triggers SnRK1, favoring stress responses (Margalha et al., 2019). Sugar metabolism and the cell cycle are adjusted by feedbacks: cell division reduces sugars and stimulates photosynthesis, while sugar accumulation can inhibit photosynthetic activity (Wingler, 2018). However, the data observed in the *aip10‐1* mutant challenge this logic, as, even with more active cell division, there is an increase in sugar levels, accompanied by lower starch accumulation. This behavior can be explained by the coordinated action of SnRK1 and TOR, sensors of cellular energy status. TOR has been associated with the promotion of vegetative and reproductive growth through the regulation of metabolism and sugar transport between source and sink tissues (Dobrenel et al., 2016; Xiong et al., 2013), while SnRK1 acts in a complementary manner under energy‐limited conditions (Baena‐González et al., 2007).

Thus, we can speculate that, in the *aip10‐1* mutant, a fine‐tuning regulation occurs, in which SnRK1 is more active in the vegetative stage, favoring the mobilization of reserves and sustaining photosynthesis, while TOR assumes greater predominance in the transition to the reproductive phase, promoting cell division and continuous growth, even in the presence of high levels of sugars. Considering the continuous growth of new organs in *aip10‐1*, both in the vegetative and reproductive phases, we hypothesize that this mutant sustains a constant demand for carbon, requiring a fine‐tuned regulation between SnRK1 and TOR to maintain active cell division and photosynthesis, even in the presence of high levels of sugars.

### Implications of the emergence and conservation of AIP10 in the plantae kingdom and its high potential for biotechnological use in plant adaptation to the environment

**Figure 10:**
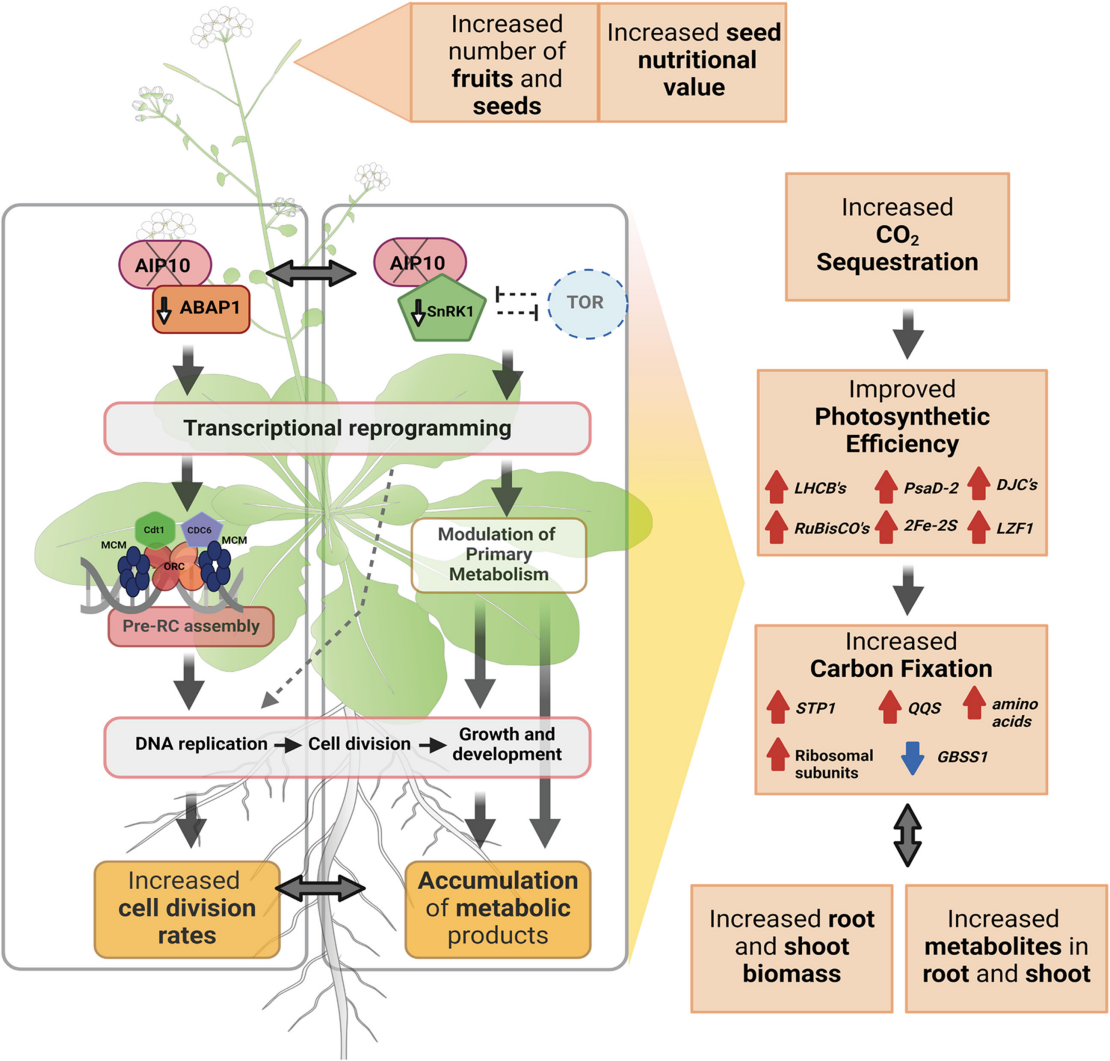
A hypothetical model of mechanisms triggered by the absence of *AIP10*. AIP10 might act by modulating plant development through interaction with ABAP1 and SnRK1 (KIN10), two key modulators of the cell cycle and primary metabolism, respectively (see text for details). In the absence of AIP10, ABAP1 transcriptional levels decrease, preventing inhibition of its targets, *CDT1a/b*, which allows the formation of the pre‐RC, licensing DNA to replicate, and cells to progress through the cycle. In parallel, the absence of AIP10 triggers a transcriptional reprogramming of targets of SnRK1, and possibly of TOR pathways, modulating cell division and plant metabolism. These processes lead to an increase in photosynthetic efficiency and results in greater carbon fixation enhanced by an increase in the number of cells, and in the increased accumulation of metabolites. A combination of all these effects contribute not only to real increase in root and shoot biomass and in seed productivity, but also to improved nutritional value of plants, and increased energy source to sustain higher cell division rates. Created with BioRender.com.

Our data showed that knockout of a single gene was able to stimulate growth through cell division and transcriptional reprogramming, leading to improved CO_2_ assimilation, increased biomass, and higher metabolic content. A proposed model of the mechanisms that might be acting in the absence of AIP10 is presented in Figure 10. AIP10 might act in a central hub that links the cell cycle with the plant primary metabolism, modulating plant development by association with ABAP1 and SnRK1 (through KIN10) and, possibly, other proteins. AIP10 knockout leads to transcription reprogramming in different plant pathways. In the absence of AIP10, ABAP1 levels and activity are reduced, releasing transcription repression of pre‐RC genes, which allows pre‐RC assembly that licenses DNA replication and cell cycle progression, culminating in increased rates of cell division. In parallel, the absence of AIP10 modulates SnRK1 activities, triggering a transcriptional reprogramming of primary plant metabolism that modulates two major pathways: (i) promotes cell divisions by providing energy, and possibly by stimulating DNA replication mediated by TOR activation; (ii) leads to an increase in photosynthetic efficiency. A coupled regulation of increased cell division rates and carbon sequestration allows a more efficient carbon fixation into root and shoot biomass. Also, the absence of AIP10 improves metabolite accumulation, serving as a large energy reserve during the vegetative phase that is metabolized in the reproductive phase, contributing to the increase in fruit and seed production with higher nutritional values.

Remarkably, plants lacking AIP10 show better improvements than plants with partial reduction (Table S5). This raises the intriguing question of why the absence of AIP10 in *A. thaliana* mutants resulted in promotion of growth and metabolism. The *AIP10* gene is found exclusively in the Plantae kingdom, and homologs were identified in all species with sequenced genomes available. Previous studies have assigned AIP10 to a set of plant‐specific genes that have only one copy in *A. thaliana* and *O. sativa*, likely originated in the chloroplast genome and being transferred horizontally to undergo stronger selection pressure, with a high probability of a critical function that promotes its conservation (Armisén et al., 2008). We can hypothesize that AIP10 is one piece in a larger gene network that might operate with gene expression homeostasis between its members, to fine‐tune the modulation of plant responses and adaptation to the environment.

Many genes and regulatory pathways evolved in the transition of plants to the terrestrial environment, allowing adaptations to water availability (Fürst‐Jansen et al., 2022), such as the emergence of stomata, optimizing the absorption of CO_2_ and loss of water, and the evolution of the cuticle acting as an extracellular hydrophobic barrier. The *aip10‐1* mutant showed several features that suggest greater adaptation to environmental fluctuations as a result of the conquest of the terrestrial environment, such as increased CO_2_ assimilation and decreased transpiration, enabling better use of water.

Our data demonstrated that modulation of a central hub that integrates the variations of cell division rates with the plant primary metabolism was an efficient strategy to improve carbon sequestration and fixation into root and shoot biomass, leading to a real increase in plant productivity and nutritional value.

## MATERIALS AND METHODS

### Plant material and growth conditions

Columbia (Col‐0), *aip10‐1* (SALK_022332), *aip10‐2* (SALK_094618), and *pAIP10*::AIP10‐YFP were obtained from the Arabidopsis Biological Resource Center (ABRC) collection. The *pAIP10::*AIP10‐YFP line (Tian et al., 2004) harbors a genomic construct of the complete *AIP10* gene (including a 3 Kb promoter/5′ UTR region and 1 Kb 3′ UTR) encoding an AIP10 protein with a C‐terminal‐YFP fusion.

Lines were confirmed by PCR genotyping using specific primers (Table S1). The *A. thaliana* seeds of *pCDT1a::*GUS were kindly provided by Dr. Crisanto Gutierrez.

Seeds were sterilized according to the protocol described by Cabral et al. (2021) and sown on glass plates containing MS medium (Murashige & Skoog, 1962) supplemented with 1% sucrose. For root analyses, plants were grown vertically on MS medium with 1.2% agar. For all other experiments, plants were grown horizontally on MS medium with 0.8% agar. The seeds were vernalized for 3 days in the dark at 4°C. After the vernalization period, the plants were maintained in a growth chamber with a 12 h/12 h photoperiod at 22°C and an intensity of 100 μmol photons m^−2^ sec^−1^. After 15 days, *in vitro* plants were transferred to a mixture of soil:vermiculite (3:1) and watered with NPK 20‐20‐20 fertilizer with a final concentration of 0.06 g/L and cultivated at 22°C with 100 μmol photons m^−2^ sec^−1^ light intensity, in a 16 h light/8 h dark cycle for seed formation. Both the mutant and Col‐0 (control) plants were cultivated in the same condition and under the same fertilizer treatment. The rosettes were weighed immediately after collection to evaluate fresh biomass. The dried biomass was obtained by drying the material in an oven at 30°C for 7 days.

### *In vitro* and *in vivo* protein interaction assay

The construct pDEST15.ABAP1 (Masuda et al., 2008) and pDEST15.AIP10 were used for the expression of ABAP1‐GST and AIP10‐GST respectively, in *E. coli* BL21 cells, as described by (Chekanova et al., 2000) with some modifications. GST pull‐down analyzes were performed following a protocol described by (Tarun & Sachs, 1996). For Western blot, input and eluted fractions were boiled in 5× SDS loading buffer with 1% b‐mercaptoethanol for 5 min at 95°C, loaded onto a Mini‐PROTEAN Tetra system (Bio‐Rad), and transferred to a PVDF membrane for 50 min at 24 V. Membranes were blocked in 5% bovine serum albumin (BSA) fraction V (Eurobio) in 1× TBS and probed for ABAP1‐AIP10 using antibodies against ABAP1 (1:1000; Covance Corp., Princeton, NJ, USA) and AIP10 (1:5000; GenScript, Piscataway, NJ, USA) in blocking buffer and probed for KIN10‐AIP10 using antibodies against KIN10 (1:1000; Agrisera, Vännäs, Västerbotten County, Sweden) and AIP10 (1:5000; GenScript) in blocking buffer, all with anti‐rabbit secondary antibody.

For ABAP1 coimmunoprecipitation assays, shoots and roots of 9‐day‐old post‐germination (9 DAG) seedlings of the *pAIP10::*AIP10‐YFP and WT (Col‐0) lines, grown in Petri dishes, were used. In addition, flower buds from the inflorescence were collected specifically for ABAP1 assays. Protein extraction was performed by grinding 200 mg of plant tissue in 400 μl of extraction buffer (20 mm sodium phosphate, pH 7.5; 500 mm NaCl; 0.1% SDS; 1% NP‐40; 0.5% sodium deoxycholate; and 0.02% sodium azide). Immunoprecipitation was performed with 300 μg of total protein extract, previously clarified with 30 μl of Sepharose beads containing 50% (v/v) protein A (GE Healthcare). The clarified supernatant was diluted 2.5‐fold (reducing the NaCl concentration to 200 mm) and incubated with anti‐ABAP1 antibody (1:2000; Covance Corp.). The beads were washed with RIPA buffer (20 mm Tris‐Cl, pH 7.4; 5 mm EDTA; 2 mm EGTA; 100 mm NaCl; 2 mm NaF; 0.2% NP‐40; 300 mm PMSF; and 10 μg ml^−1^ aprotinin and pepstatin).

For the KIN10 assay, shoots from pAIP10::AIP10‐YFP and WT (Col‐0) seedlings grown in Petri dishes were collected 9 days after germination. Total protein extraction was obtained by grinding 2 g of tissue using 5 mL of EB+ buffer (150 mm NaCl, 1% Nonidet P‐40, 50 mm Tris–HCl pH 8, 5 mm DTT, 0.2 mm PMSF and PIC 1×). Samples were sonicated (75% power, 3 × 15 sec ON/15 sec OFF) and, after 30 min on ice, diluted to 0.2% NP40, centrifuged, and filtered. For immunoprecipitation, 5 ml of total protein extract (input) was mixed with 20 μl of GFP‐Trap A beads (ChromoTek, Germany) and incubated overnight at 4°C with slow rotation. The beads containing immunoprecipitated proteins were washed three times for 5 min at 4°C with wash buffer (150 mm NaCl, 0.1% Nonidet P‐40, 50 mm Tris–HCl pH 8, 5 mm DTT, 0.2 mm PMSF and PIC 1×) and twice with 50 mm ammonium carbonate buffer. For Western blot, input and eluted fractions were boiled in 2× SDS loading buffer with 1% b‐mercaptoethanol for 5 min at 95°C, loaded onto a Mini‐PROTEAN Tetra system (Bio‐Rad), and transferred to a PVDF membrane for 1 h at 100 V. Membranes were blocked in 5% bovine serum albumin (BSA) fraction V (Eurobio) in 1× TBS and probed for ABAP1‐AIP10 using antibodies against ABAP1 (1:1000; Covance Corp.) and YFP (anti‐GFP, 1:2000; Invitrogen, Carlsbad, CA, USA) in blocking buffer, both with anti‐rabbit secondary antibody. Anti‐ABAP1 polyclonal antibody was developed against the peptide antigen GAPIVTQLID (amino acids 28–37) by Covance Corp. For KIN10‐AIP10 co‐ip, LivingColors^®^ GFP monoclonal antibody (1:2000; Takara Bio, San Jose, CA, USA) and anti‐mouse antibody were used as secondary antibodies, and KIN10 polyclonal antibody (Agrisera; 1:1000) and anti‐rabbit secondary antibodies were used. All antibodies were diluted in 0.1% TBST. Blots were developed with Clarity™ Western ECL substrate according to the manufacturer's instructions (Bio‐Rad).

### Yeast two‐hybrid assay

The yeast two‐hybrid interaction assay was performed by using strain PJ694‐a (MATa trp1‐901 leu2‐3112 ura3‐52 his3‐200 gal4Δ gal80Δ LYS2::GAL1‐HIS3 GAL2‐ADE2 met2::GAL7‐lacZ). The ABAP1 regulatory network proteins tested in this article were obtained from the laboratory's clone bank. KIN10 vectors were purchased from Clones/DNA Resources – Other BAC Libraries – TAIR. For the construction of KIN11(A11), its cDNA was amplified by PCR (Table S1).

### Microscopy and kinematic analysis

For subcellular localization of AIP10‐YFP, seedlings of the *pAIP10*::AIP10‐YFP line at different developmental stages and tissues were observed under a confocal fluorescence microscope (Zeiss LSM510 META) using YFP filters with an excitation laser of 405 nm, and the fluorescence was collected between 431 and 532 nm.

The meristem size was determined by delineating the zone between the stem cells around the quiescent center and the farthest cell from this center, still in the process of division. Photographs were taken with a magnification of 5 μm, and the root meristem size was subsequently measured using IMAGE J software. Mean values were compared using the Student's *t*‐test for independent samples, with differences considered significant for *P* ≤ 0.05. For kinematic analyses, the first pair of leaves between 6 and 18 DAG (*n* = 3–5 plants) was used, following the protocol described by De Veylder et al. (2001). The leaves were incubated overnight in a solution of absolute ethanol and acetic acid (9:1) and then clarified by incubation in 0.025% chloral hydrate diluted in 30% glycerol solution. The sections were observed by differential interference contrast (DIC) optical microscopy (Leica DM2500 CSQ), with images captured by the Leica Application Suite X (LAS X) system. The leaf blade was photographed using a Zeiss Stemi SV11 magnifying glass coupled to the Leica DFC290 HD capture system. The abaxial epidermal cells of the apical and basal regions of the leaf were outlined and analyzed with the ImageJ software. Calculations were performed as described by Nelissen et al. (2012). Histochemical detection of GUS activity in 5‐day‐old homozygous pCDT1a::GUS lines, in WT Col‐0 and *aip10‐1* backgrounds, was done with 5‐bromo‐4‐chloro‐3‐indolyl β‐d‐glucuronide according to Cabral et al. (2021).

### Expression analysis by RT‐qPCR

Total RNA was extracted according to Logemann et al. (1987) and treated with RNAse‐free DNase I (New England Biolabs^®^). cDNA was performed using SuperScript™ III Reverse Transcriptase (Invitrogen) and the SYBR Green PCR Master Mix kit (Applied Biosystems) was used for expression analysis. Normalization was done against the average of the reference genes, *Ubiquitin 14* and *GAPDH* using the linear scaling method. The sequences of primers used in the RT‐qPCR experiments are listed in Table S1.

### Flow cytometry analysis

Samples consisted of the first pair of leaves at 14 DAG and 20 DAG that were placed in Petri dishes containing 400 μl of buffer (hypotonic buffer containing a non‐ionic detergent) and chopped with a razor blade to release nuclei. Each macerate was resuspended in the buffer and filtered through a 50 μm filter. Nuclei were incubated in the dark with 1 mg ml^−1^ 4′,6‐diamidino‐2‐phenylindole (DAPI), and subsequently analyzed with a BRYTE HS Flow Cytometer (Bio‐Rad). Analyses were performed in biological triplicates.

### Photosynthetic analysis

Chlorophyll *a*, chlorophyll *b*, and total carotenoids were extracted from plants at 11 and 35 DAG using dimethyl sulfoxide (DMSO) as solvent. For each sample, two rosette discs of the fifth leaf were weighed and immediately submerged in microtubes containing 1 ml of DMSO. Samples were kept in the dark at room temperature for 72 h. Following incubation, pigment quantification was performed in technical duplicate by spectrophotometry (NanoDrop™ 2000; Thermo Fisher Scientific), with absorbance readings taken at 480, 649.1, and 665.1 nm. Pigment concentrations were calculated using the following equations: Ca = 12.74 × A665.1 − 3.62 × A649.1; Cb = 25.06 × A649.1 − 6.5 × A665.1; and Cx + c = (1000 × A480 − 1.29 × Ca – 53.78 × Cb)/220. Results were expressed as micrograms of pigment per gram of fresh tissue (μg g^−1^). To record CO_2_ assimilation in response to photosynthetic light, measurements were performed in the morning using the LI‐6400XT portable photosynthesis system (LI‐COR Biosciences, USA), between 8:00 a.m. and 10:00 a.m., adjusting the CO_2_ concentration to 400 ppm, and the photon flux density (PPFD) at 100 μmol m^−2^ sec^−1^, with leaf temperature maintained between 21 to 22°C. The flux of CO_2_ and H_2_O was converted into photosynthesis (*A*_net_) μmol CO_2_ m^−2^ sec^−1^ and transpiration (*E*) mmol H_2_O m^−2^ sec^−1^. Water use efficiency (WUE) was calculated as *A*_net_/*E* (μmol CO_2_ mmol H_2_O^−1^). Canopy conductance (*g*_c_) was estimated using the formula *g*_c_ = (*E*/DPV) × 101.325. To measure the efficiency of photosystem II, a closed fluorescence imaging device (Fluorcam 800 MF; Photon Systems Instruments) was used, in which 15 plants of each genotype were analyzed to measure the Fv/Fm ratio and NPQ. The results were analyzed using the FluorCam7 software (Closed FluorCam FC 800‐C).

### Metabolic profiling via ATR‐FTIR spectra acquisition

The metabolic profile spectra were obtained using a Shimadzu IRPrestige‐21 total attenuated reflectance infrared spectrometer (ATR‐FTIR) with a diamond crystal plate (IRIS module; PIKE Technologies). The results were normalized using the crystal surface of the ATR accessory. A pool of 100 macerated seeds was used, enough to cover the entire surface of the crystal of the ATR accessory and the leaves, two distinct regions were divided, and measurements were taken to obtain a spectrum representing the entire sample and three different biological replicates. All data processing was performed in an internal computational routine implemented in Matlab 2021a^®^ (The MathWorks Inc., Natick, USA). The second derivative was also applied and tested with Savitz‐Golay smoothing using a second‐order polynomial and a 7‐point window, which removes not only simple additive shifts but also first‐order effects such as baseline shift and spectral noise.

### Total soluble sugars, starch assays

Col‐0, *aip10‐1*, and *aip10‐2* plants were grown until reaching 20, 35 DAG and the reproductive stage when green siliques were harvested. Biological samples were collected in quadruplicate, immediately immersed in liquid nitrogen and stored in an ultrafreezer (−80°C) for later analysis of soluble sugars and starch. The extraction of soluble sugars was performed according to the methodology described by Garcia et al. (2006), using four replicates of 80 mg of leaves per treatment. The quantification of total soluble sugars and sugars released by starch hydrolysis was performed by spectrophotometry, using the anthrone colorimetric method (Yemm & Willis, 1954) and a glucose standard curve (0–100 μg ml^−1^). Starch was extracted from the pellet obtained during the extraction of soluble sugars and hydrolyzed with 52% perchloric acid to release reducing sugars.

### RNA‐Seq and transcriptome analysis

The RNA‐Seq libraries were constructed with total RNA from two shoot and root repeats of 11 DAG and three shoot and root repeats of 35 DAG by Fasteris Life Sciences SA. Eight libraries (11 DAG) and 12 libraries (35 DAG) were prepared according to the protocol available on the website and sequenced on the Illumina HISeq2500 (11 DAG) (single‐end) and NovaSeq 6000 (35 DAG) (paired‐end). The raw reads were subjected to control quality (Q30 >80%) and adapter trimming using Trimmomatic software. Mapping was performed using the TAIR10 genome as a reference using Bowtie software. Expression estimation, normalization and differential expression was performed using Cufflinks software. Libraries were normalized using the FPKM. Differential expression analysis was performed, with significance determined by the *q*‐value, which was calculated using the Benjamini–Hochberg correction (FDR) for multiple testing. Genes with a *q*‐value ≤0.05 were considered significant. The differentially expressed genes (DEGs) were compared between the libraries, based on the difference in expression, generating FoldChange (FC) values classified as induced or repressed based on the Log calculation in base 2 of the FC values comparing the FPKM values of the library of interest against the control. Pathway enrichment analysis was performed using g:Profiler and maps were visualized with EnrichmentMap in the Cytoskape software.

For transcriptome comparisons, datasets were obtained from Baena‐González et al. (2007) (KIN10 overexpression), Dong et al. (2015) (AZD8055 TOR inhibition treated) and Forzani et al. (2019) (*lst8* TOR mutant). In the Forzani set, deSEQ2 was used for differential analysis. Log_2_ fold change variables were used to compare common genes among datasets. After batch correction, non‐parametric Spearman's rank correlation coefficient was calculated to assess the monotonic relationship between the datasets.

### Sequence alignment and phylogenetic analysis

Multiple protein sequence alignment was performed from available database sequences from species show in Tables S2 and S3 and was conducted using CLUSTALW with default parameters. Subsequently, phylogenetic trees were generated using the IQTREE software through Maximum Likelihood Analysis, employing the JTT + Invar (I) + Gamma (G) model with four categories and subjected to 1000 repetitions.

### Statistical analysis

Statistical analysis was used following the number of variables. An unpaired Student's *t*‐test was performed between two samples (*P < 0.05). Tukey's one‐way analysis of variance was used to test the significance level of multiple groups and using *P* < 0.05 was considered to indicate statistical significance represented in letters.

## AUTHOR CONTRIBUTIONS

The project was designed by ASH. The experiments were carried out by PM, JFRP, CNMC, LD, LPG, VR, AFF, HFB, VI, LMC, FSC, BGdA‐L, JB, and JdAE. Measurements using the ATR‐FTIR equipment were carried out by PM, JCdA, LT, and CAC‐J; and measurements with the equipment Li‐Cor 6400XT were made by PM, WdPB, and EC. The manuscript was written by PM and JFRP, with contributions from the other authors. Reviewing and editing was performed by ASH. ASH is the principal investigator. All authors have read and agreed to the published version of the manuscript.

## CONFLICT OF INTEREST

The authors declare that there is no conflict of interest.

## Supporting information


**Figure S1.** AIP10 interaction with ABAP1 and KIN10 was tested in *semi‐in vivo* pulldown assays, with bacterially expressed recombinant AIP10‐GST and Arabidopsis protein extracts.
**Figure S2.** Protein interaction screening by yeast two‐hybrid to identify potential AIP10 binding partners.
**Figure S3.** Expression pattern of AIP10 isoforms in different *A. thaliana* tissues.
**Figure S4.** Phylogenetic analysis of AIP10 and its putative orthologs.
**Figure S5.** Phylogenetic analysis of ABAP1 and its putative orthologs.
**Figure S6.** Molecular characterization of *AIP10* mutant lines.
**Figure S7.** Experimental repetition of the phenotypic evaluation of *AIP10* knock‐out (*aip10‐1*) and knock‐down (*aip10‐2*) Arabidopsis plants compared to wild‐type Col‐0.
**Figure S8.** Experimental repetition of cell division analysis in *aip10‐1*, *aip10‐2*, and wild‐type Col‐0 plants.
**Figure S9.** Analysis of root and leaf growth parameters in *aip10‐1*, compared with wild‐type Col‐0.
**Figure S10.** Analysis of *CDT1a* expression in meristems of *aip10‐1* plants compared to wild‐type Col‐0.
**Figure S11.** Average nuclear DNA content analyzed by flow cytometry.
**Figure S12.** Comparative analysis of transcriptomic profiles between KIN10oe and *lst8* datasets with *aip10‐1* mutants.
**Figure S13.** Comparative analysis of the expression profile of genes modulated by TOR inhibition differentially expressed in *aip10‐1*.
**Figure S14.** Comparative analysis of transcriptomic profiles between iTOR and *lst8* datasets with *aip10‐1* mutants.
**Figure S15.** Analysis of total soluble sugar and starch content in source and sink tissues.
**Figure S16.** Loading chart for the first two main components applied to the ATR‐FTIR dataset.
**Figure S17.** Metabolic analysis by ATR‐FTIR spectrum of *aip10‐1* and *aip10‐2* plants at 20 DAG, compared with wild‐type Col‐0.
**Table S1.** Primers used in this study.
**Table S2.** AIP10 putative orthologs in other plant species.
**Table S3.** ABAP1 putative orthologs in other plant species.
**Table S4.** Attributions for the main vibrational bands identified in the bio‐fingerprint region.
**Table S5.** Percentage differences in physiological parameters of KO and KD lines relative to Col‐0.

## Data Availability

The data that support the findings of this study are available from the corresponding author upon reasonable request. The RNA sequencing data generated in this study have been deposited in the National Centre for Biotechnology Information (NCBI) (https://www.ncbi.nlm.nih.gov/sra/?term=PRJNA1070685).
