# Supplementary material for: The ABAP1 interacting protein 10 (AIP10) exerts a dual role in the cell cycle and primary metabolism pathways in Arabidopsis thaliana
Source: Plant J. 2025 Aug 8;123(3):e70399. doi: 10.1111/tpj.70399 (PMC12334247; doi:10.1111/tpj.70399)
Supplement: Supplementary file 1 — Figure S1. AIP10 interaction with ABAP1 and KIN10 was tested in semi‐in vivo pulldown assays, with bacterially expressed recombinant AIP10‐GST and Arabidopsis protein extracts. Figure S2. Protein interaction screening by yeast two‐hybrid to identify potential AIP10 binding partners. Figure S3. Expression pattern of AIP10 isoforms in different A. thaliana tissues. Figure S4. Phylogenetic analysis of AIP10 and its putative orthologs. Figure S5. Phylogenetic analysis of ABAP1 and its putative orthologs. Figure S6. Molecular characterization of AIP10 mutant lines. Figure S7. Experimental repetition of the phenotypic evaluation of AIP10 knock‐out (aip10‐1) and knock‐down (aip10‐2) Arabidopsis plants compared to wild‐type Col‐0. Figure S8. Experimental repetition of cell division analysis in aip10‐1, aip10‐2, and wild‐type Col‐0 plants. Figure S9. Analysis of root and leaf growth parameters in aip10‐1, compared with wild‐type Col‐0. Figure S10. Analysis of CDT1a expression in meristems of aip10‐1 plants compared to wild‐type Col‐0. Figure S11. Average nuclear DNA content analyzed by flow cytometry. Figure S12. Comparative analysis of transcriptomic profiles between KIN10oe and lst8 datasets with aip10‐1 mutants. Figure S13. Comparative analysis of the expression profile of genes modulated by TOR inhibition differentially expressed in aip10‐1. Figure S14. Comparative analysis of transcriptomic profiles between iTOR and lst8 datasets with aip10‐1 mutants. Figure S15. Analysis of total soluble sugar and starch content in source and sink tissues. Figure S16. Loading chart for the first two main components applied to the ATR‐FTIR dataset. Figure S17. Metabolic analysis by ATR‐FTIR spectrum of aip10‐1 and aip10‐2 plants at 20 DAG, compared with wild‐type Col‐0. Table S1. Primers used in this study. Table S2. AIP10 putative orthologs in other plant species. Table S3. ABAP1 putative orthologs in other plant species. Table S4. Attributions for the main vibrational bands identified [file TPJ-123-e70399-s001.pdf]

Article title: **The ABAP1 INTERACTING PROTEIN 10 (AIP10) exerts a dual role in the cell cycle and primary metabolism pathways in *Arabidopis thaliana*.**

Authors: Patrícia Montessoro[1†]; Joaquin Felipe Roca Paixão[1†]; Carinne N M Costa[1]; Laura Ducatti[1]; Letícia Perdigão Grangeiro[1]; Vivian Ruivo[1]; Adriana Flores Fusaro[1]; Helkin F Ballesteros [1]; Vanessa Iurif[1], Luiz Mors Cabral [1,2]; Jelmir Craveiro de Andrade[3]; Leticia Tessaro[3]; Wallace de Paula Bernado[4]; Fernanda Silva Coelho[5]; Bruna Gino de Araújo-Lopes[1]; Janice de Almeida Engler[6]; Jérémie Bazin[7]; Eliemar Campostrini[4]; Carlos Adam Conte-Junior[3]; and Adriana Silva Hemerly [1*]

The following Supporting Information is available for this article:

Figure S1. AIP10 interaction with ABAP1 and KIN10 was tested in *semi-in vivo* pulldown assays, with bacterially expressed recombinant AIP10-GST and Arabidopsis protein extracts. a) AIP10-GST fusion tested against ABAP1-GFP and Col-0 protein extracts. ABAP1 and AIP10 interaction is shown in lane 5. ABAP1 interacting protein was assayed with anti-ABAP1 antibody in immunoblot. AIP10-GST was assayed with a specific anti-AIP10 antibody. Numbers 1–8 refer to the lanes on the SDS-PAGE. b) AIP10-GST fusion tested against Col-0 protein extract. KIN10 and AIP10 interaction is shown in lane 2. KIN10 interacting protein was assayed with anti-KIN10 antibody in immunoblot. AIP10-GST was assayed with a specific anti-AIP10 antibody. Numbers 1–3 refer to the lanes on the SDS-PAGE.

(a)

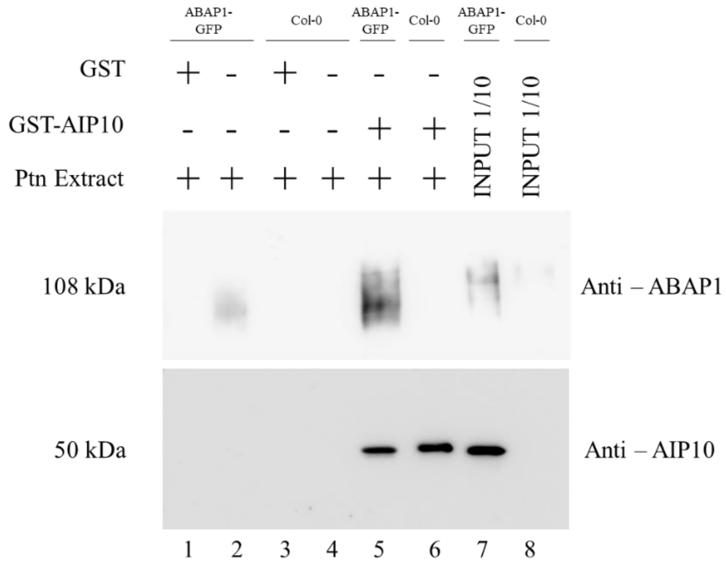

(b)

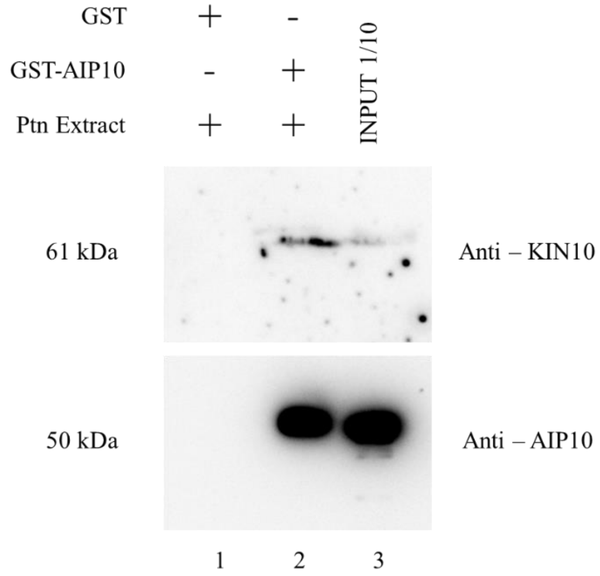

Figure S2. Protein interaction screening by yeast two-hybrid to identify potential AIP10 binding partners. AIP10 protein interaction with ABAP1, ARIA, KIN11 and pre-replication complex components ORC1-6, CDT1 and CDC6 was evaluated. The empty vectors GAL4 AD and GAL4 BD were used as negative controls, while interaction of AIP10 with ABAP1 was used as positive control. Growth of yeast strain on selective media SD-Leucine/-Tryptophan confirmed transformation with both BD/AD constructs. Growth on stringent selective media SD-Leucine/-Tryptophan/-Histidine/-Adenine confirmed strong protein interactions between AIP10 and ABAP1, and indicated that this interaction was mediated by the Armadillo domain of ABAP1. A11 (SnRK1 CATALYTIC SUBUNIT KIN11); ARIA (ARM REPEAT PROTEIN INTERACTING WITH ABF2); ORC (ORIGIN RECOGNITION COMPLEX); ARM (ARMADILLO REPEAT DOMAIN OF ABAP1); BTB (BTB/POZ DOMAIN OF ABAP1).

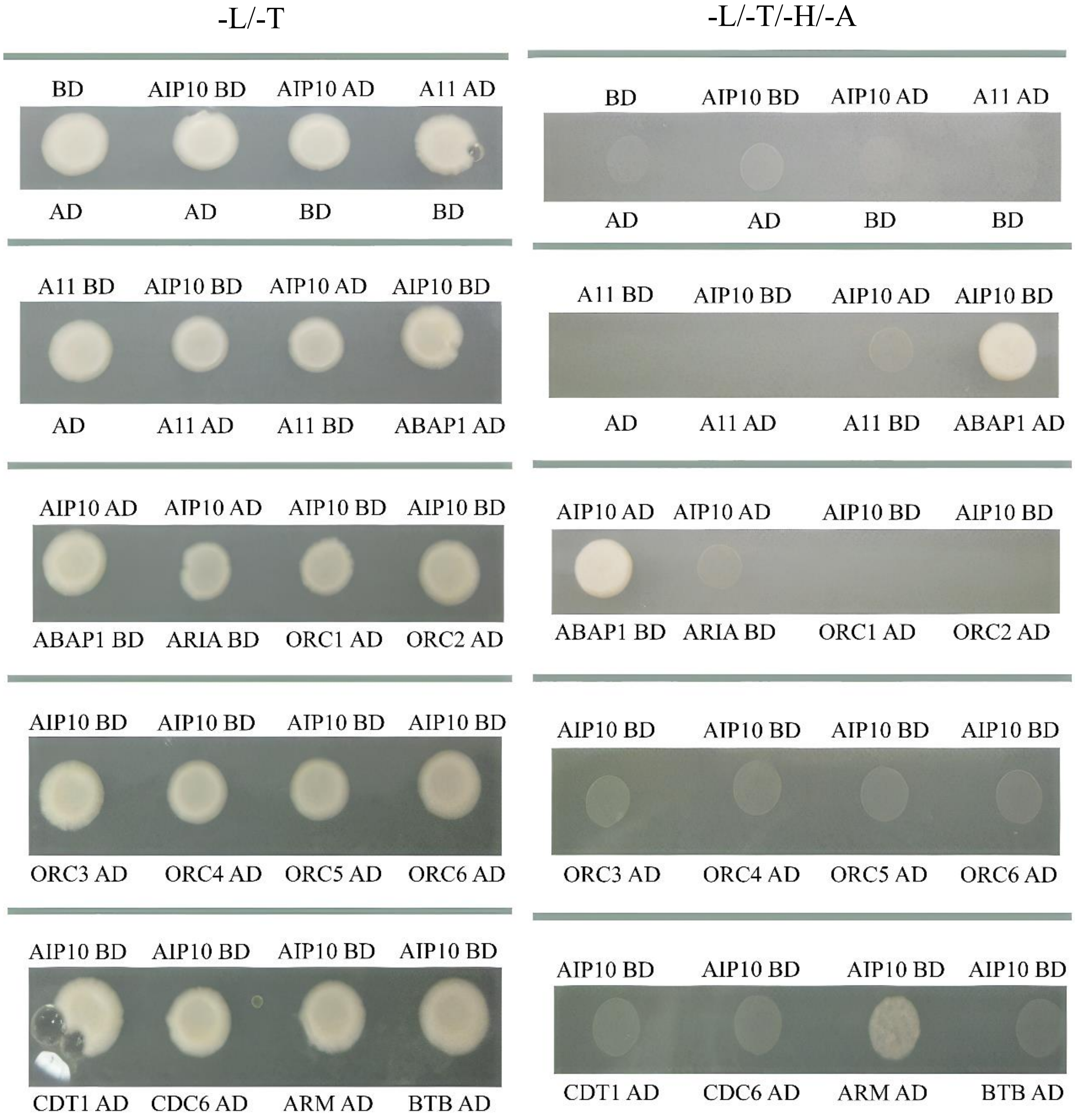

Figure S3. Expression pattern of *AIP10* isoforms in different *A. thaliana* tissues. Characterization of the expression pattern of *AIP10.1*, *AIP10.2*, *AIP10.3* and *AIP10.4* isoforms in 11 DAG roots grown *in vitro*; and young 11 DAG seedlings, mature 32 DAG rosettes, floral bud, open flowers and siliques of Col-0 plants grown directly *in vivo*, under a 16h/8h light photoperiod. Expression levels were normalized by the mRNA levels of the *UBI14* and *GAPDH* genes. Bars represent means ± SD (standard deviation) and * significantly different from Col-0 with p≤0.05 with Student's test. (n=3 biological replicates). The primers were designed in regions that vary between the isoforms and are individually represented in the diagram in Figure S5.

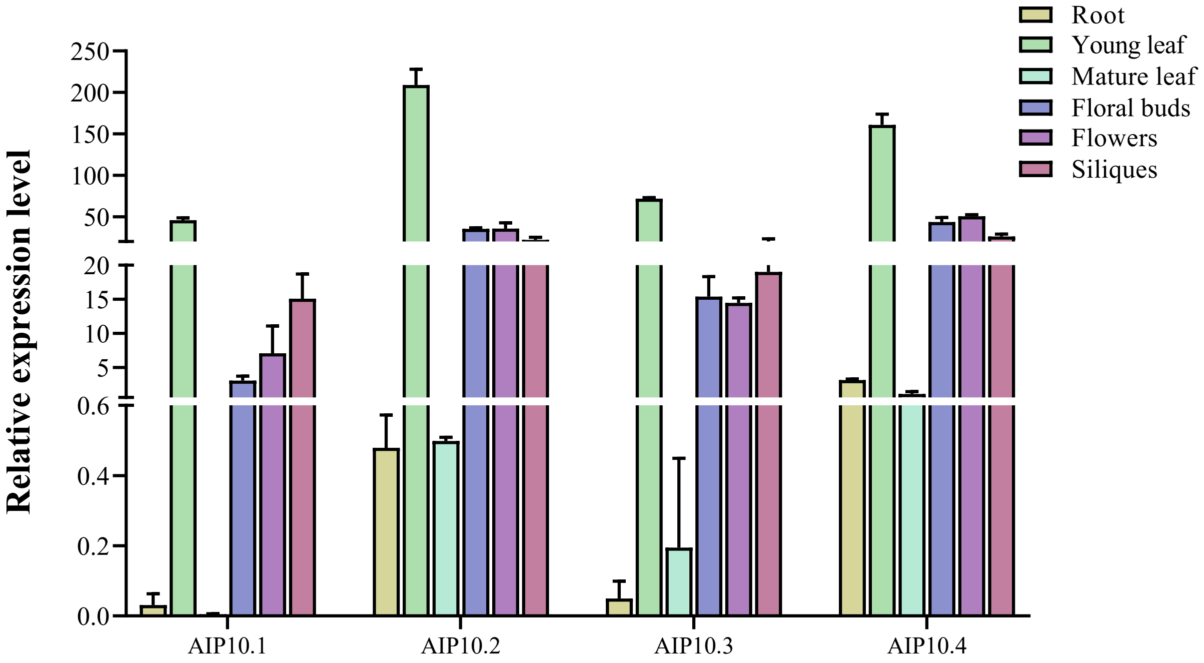

Figure S4. Phylogenetic analysis of AIP10 and its putative orthologs. a) Phylogenetic tree was generated in the IQTREE software using the Maximum Likelihood method using the JTT + Invar (I) + Gamma (G) model with 4 categories subjected to the Bootstrap phylogeny test (1000 repetitions). b) Amino acid alignment performed using the Jalview software using the CLUSTAL W method. The image comprises the AIP10 C-terminal region, conserved in all species and which contains the conserved “Coiled coil” domain, a nuclear localization signal (NLS) and a PPS. The protein sequences used in this tree were obtained from the Phytozome, Plaza and Sol Genomics databases. Black bars: scale range 3.0.

(a)

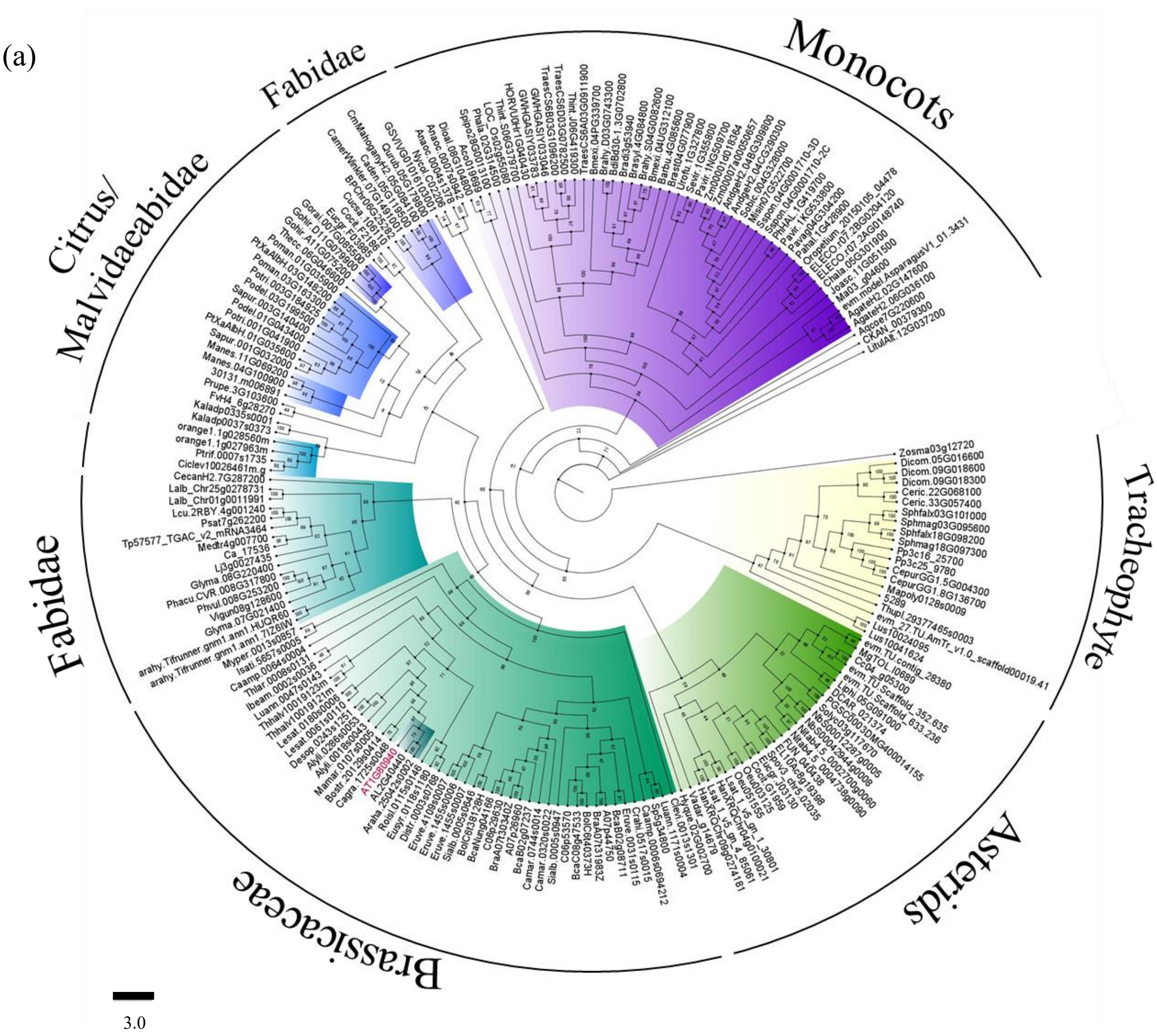

(b)

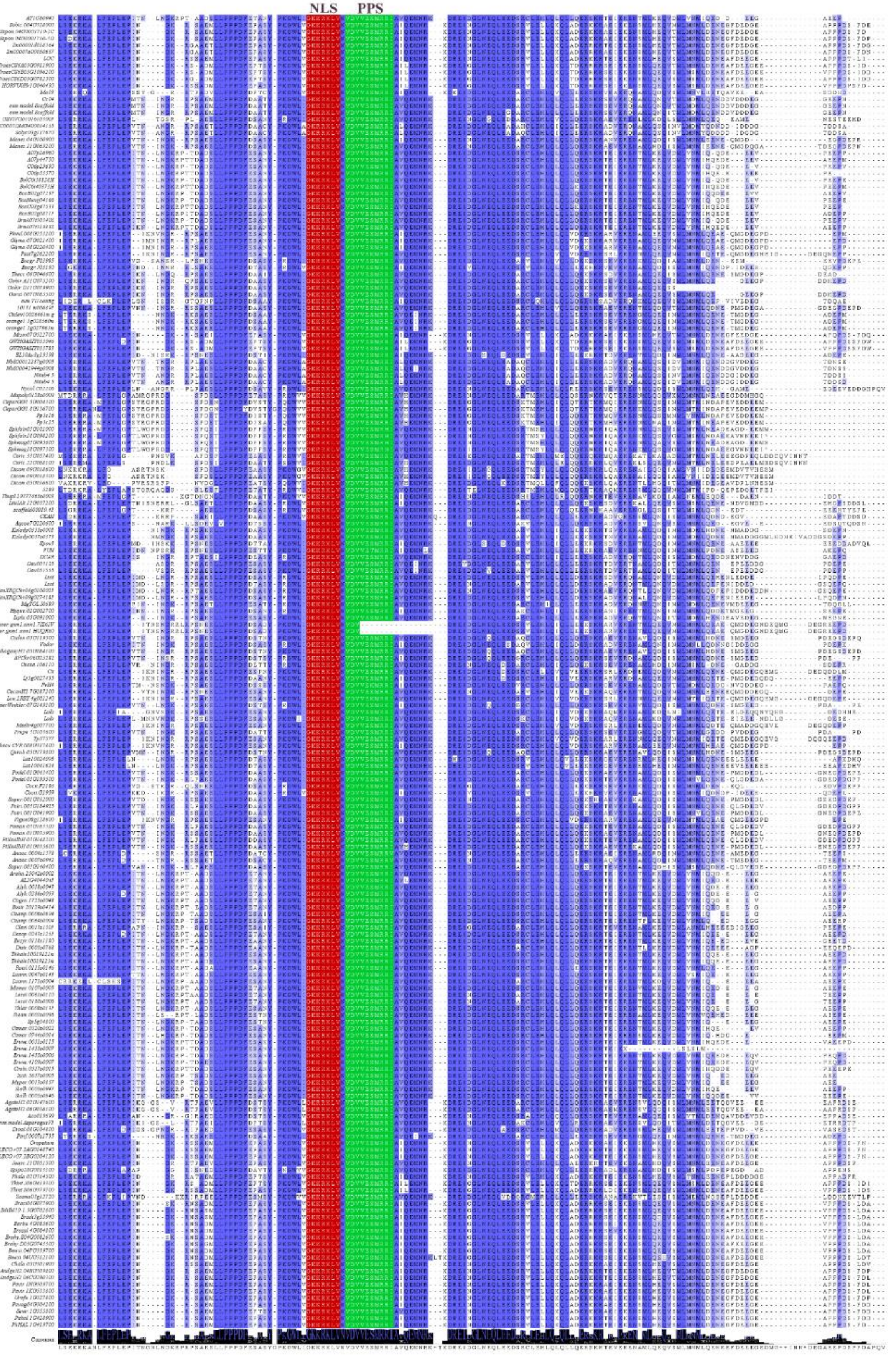

Figure S5. Phylogenetic analysis of ABAP1 and its putative orthologs. The phylogenetic tree was generated in the IQTREE software using the Maximum Likelihood method using the JTT + Invar (I) + Gamma (G) model with 4 categories subjected to the Bootstrap phylogeny test (1000 repetitions). The protein sequences used were obtained from the Phytozome, Plaza and Sol Genomics databases. black bars: scale range 4.0.

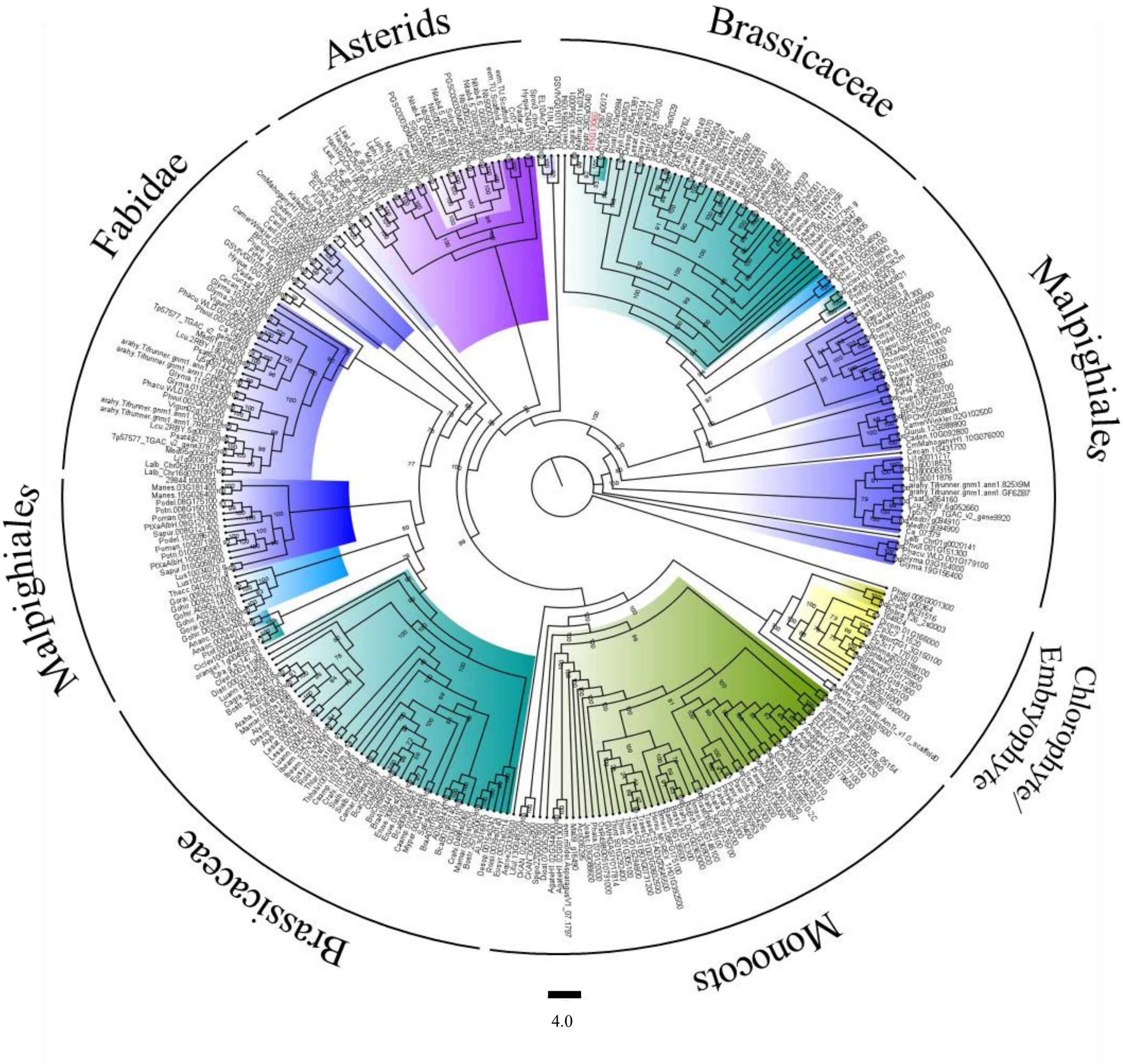

Figure S6. Molecular characterization of *AIP10* mutant lines. a) Map of *AIP10* alternatively spliced isoforms showing the T-DNA insertion site in Salk mutants. Exons are represented by yellow rectangles and gray ones correspond to the 5' and 3' UTR regions. The solid gray line represents the intronic regions and the dotted black line represents the estimated position of the T-DNA in each alternative splicing isoforms of *AIP10*. Specific primers were used for each isoform to evaluate relative expression, as indicated in Figure S2**.** Brown arrows represent primers for isoform *AIP10.1*, green arrows for *AIP10.2*, blue arrows for *AIP10.3*, and pink arrows for *AIP10.4*. b) *AIP10* expression in *aip10-1* (Salk_022332) and *aip10-2* (Salk_094618) seedlings relative to Col-0 was analyzed by RT-qPCR. The positions of the primers are indicated as qRT.Fw and qRT.Rev. Expression levels were normalized by the mRNA levels of the *UBI14* and *GAPDH* genes. Bars represent means ± SD (standard deviation) and * significantly different from Col-0 with p≤0.05 with Student's test. (n=3 biological replicates).

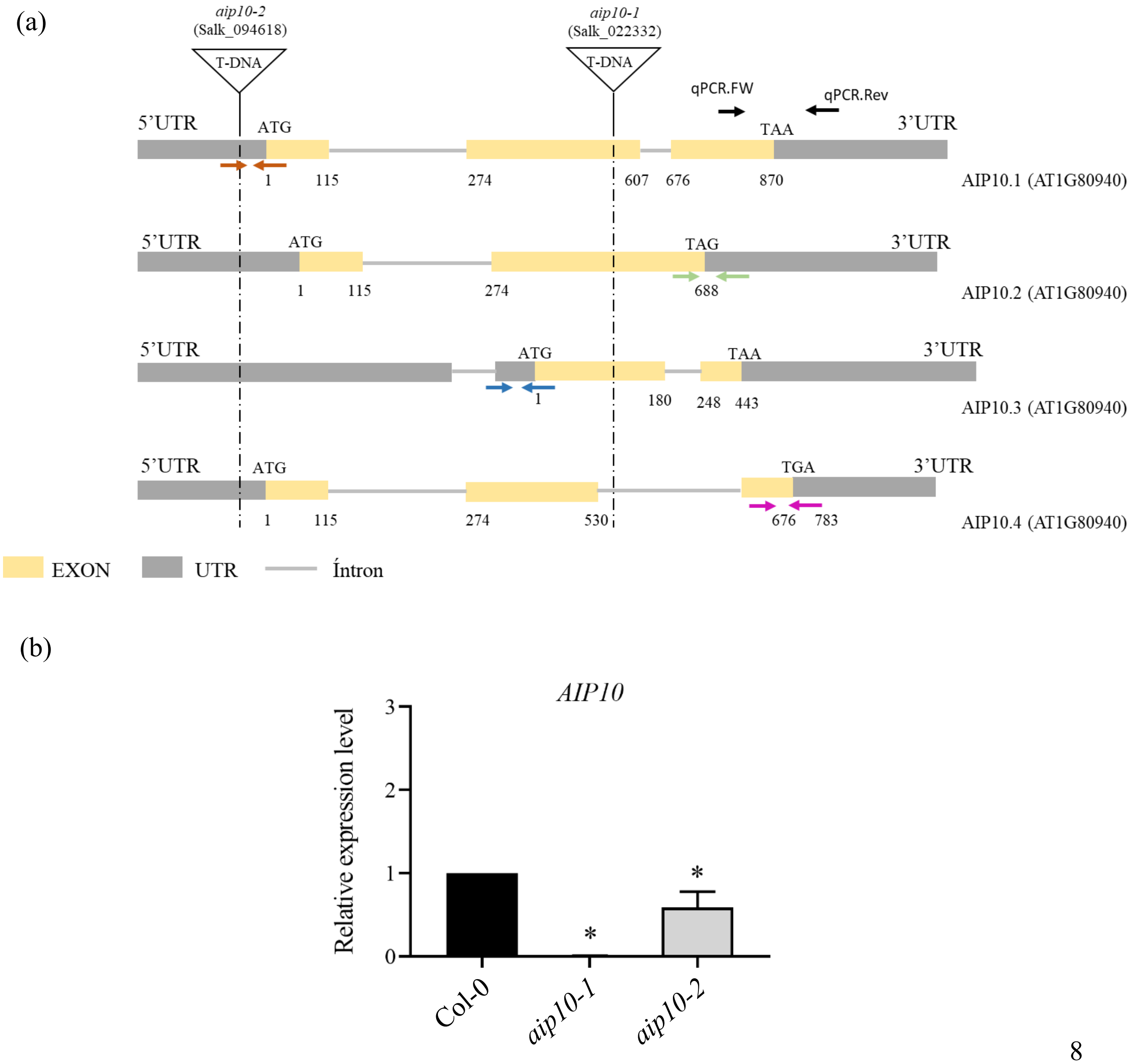

Figure S7. Experimental repetition of the phenotypic evaluation of *AIP10* knock-out (*aip10-1*) and knock-down (*aip10-2*) Arabidopsis plants compared to wild-type Col-0. Experiments were carried out in a photoperiod of 16h/8h at 21ºC, with seeds cultivated directly in the soil. Representative images of a) wild-type Col-0, *aip10-1* and *aip10-2* plants at 30 DAG, b) at 35 DAG and c) at 57 DAG during the reproductive stage.

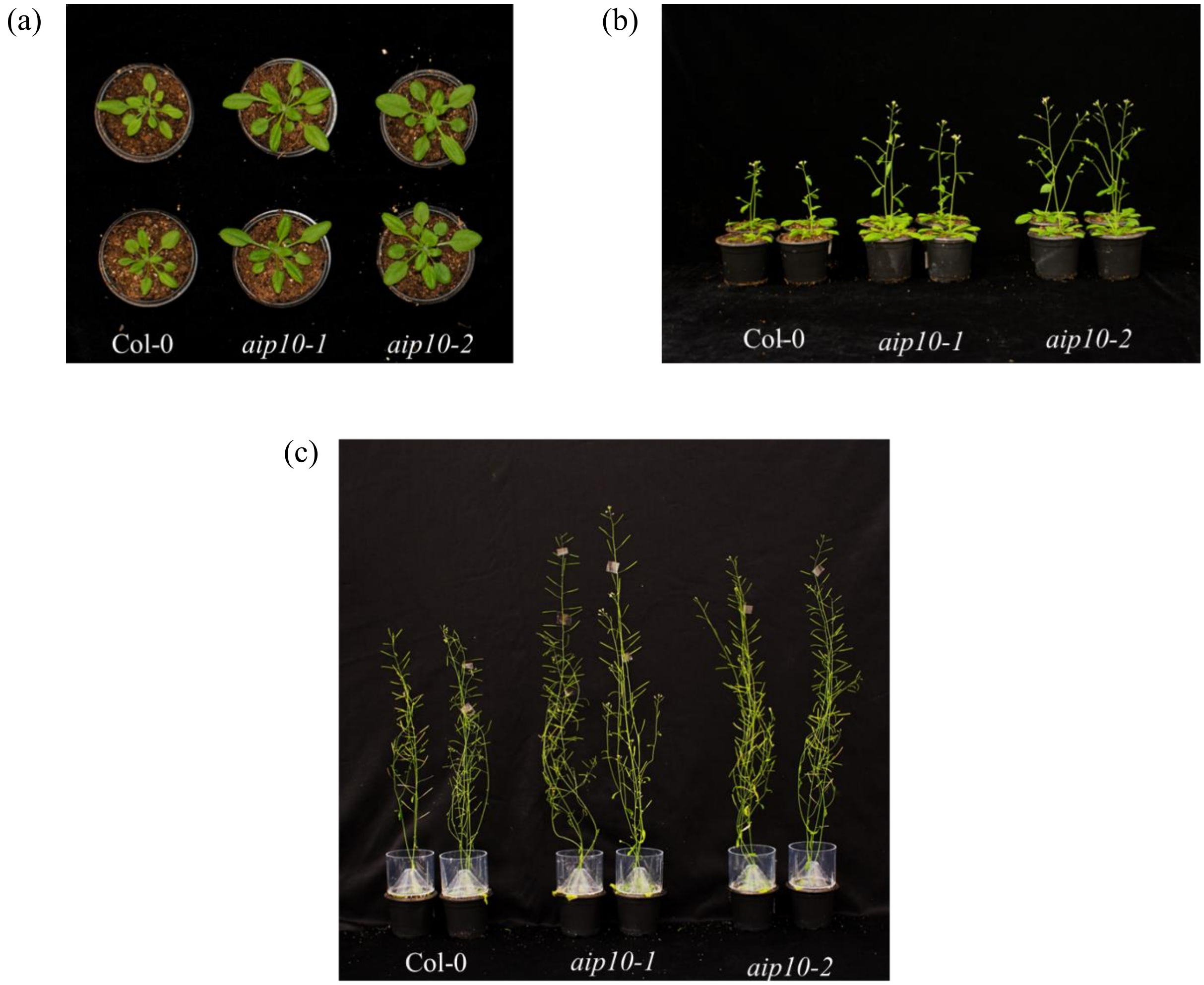

Figure S8. Experimental repetition of mRNA expression analysis of cell division genes in *aip10-1*, *aip10-2* and wild-type Col-0 plants. Expression levels of *ABAP1*, *CyclinB1;1*, *CyclinB1;2* and the ABAP1 target genes *CDT1A* and *CDT1B* in Col-0, *aip10-1* and *aip10-2* plants. Relative mRNA levels were assessed in both genotypes in shoots of 11DAG seedlings grown *in vitro* on MS medium with 1% sucrose in a 12h/12h photoperiod at 21°C. Each biological replicate (n = 3) consisted of a set of 10 plants. Data were normalized with the expression of *UBI14* and *GAPDH* as reference genes. Bars indicate the standard deviation of biological replicates. Statistical analysis was performed using Student's t-test (p≤0.05). Asterisks (*) indicate significant differences between samples in relation to Col-0.

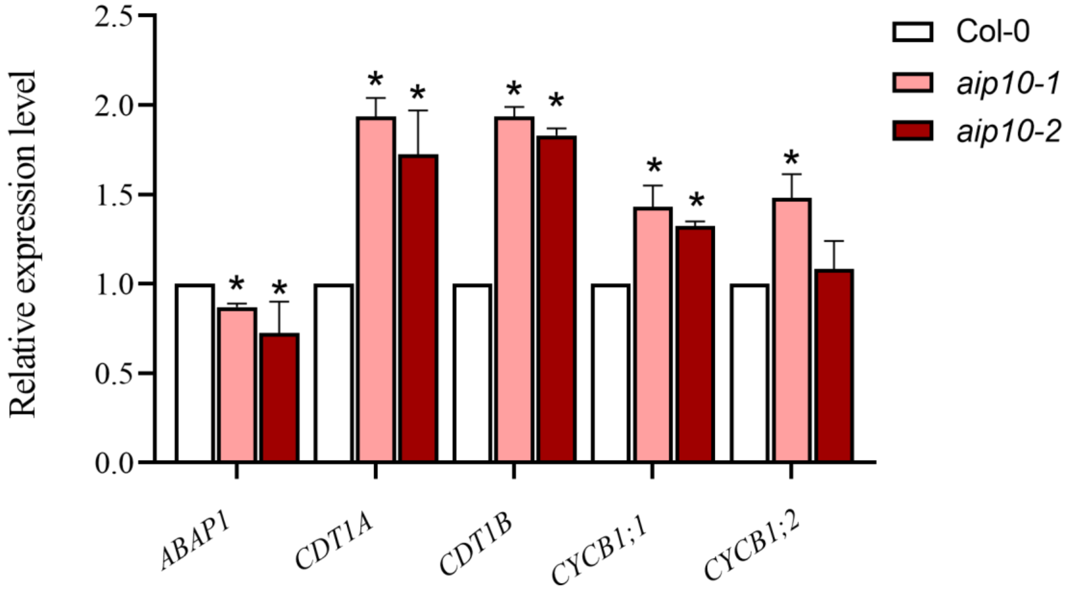

Figure S9. Analysis of root and leaf growth parameters in *aip10-1,* compared with wild-type Col-0. a) Expression analysis of *ABAP1, CyclinB1;1*, *CyclinB1;2* and the ABAP1 target genes, *CDT1a* and *CDT1b,* in Col-0 and *aip10-1*. Relative mRNA levels were evaluated in both genotypes in 11 DAG roots cultivated *in vitro* in MS medium with 1% sucrose in a 12h/12h photoperiod at 21ºC. Expression levels were normalized by the mRNA levels of the *UBI14* and *GAPDH* genes. Bars represent means ± SD (standard deviation) and * significantly different from Col-0 with p≤0.05 with Student's test. (n=3 biological replicates). b) Primary root length (n=10). c) Number of lateral roots (n=10). d) Leaf area and e) Cell area evaluated by kinematic growth analysis of the first leaf pair, from 6 to 18 DAG. Bars represent means ± SD (standard deviation) of the 5 plants.

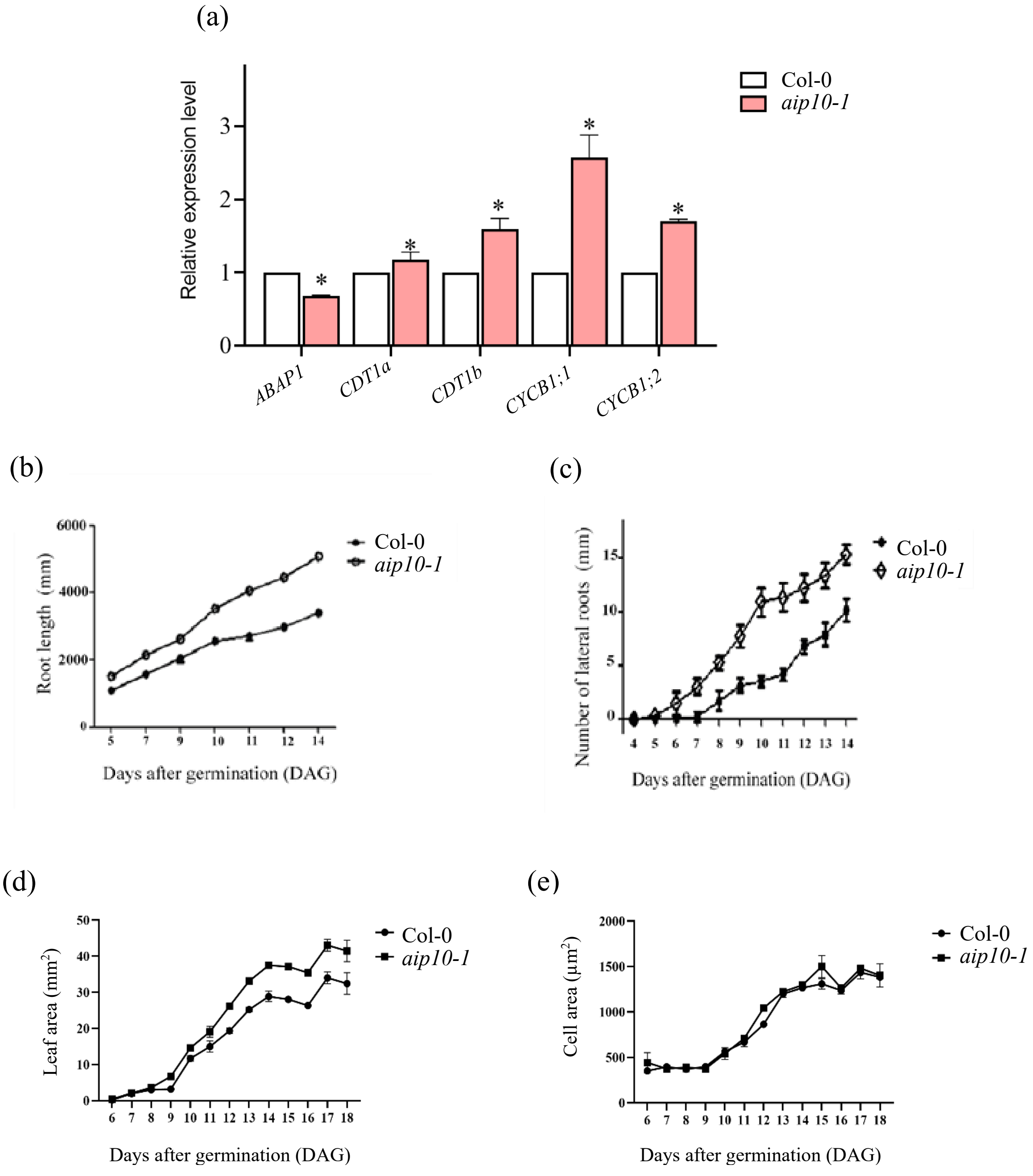

Figure S10. Analysis of *CDT1a* expression in meristems of *aip10-1* plants compared to wild-type Col-0. a) GUS staining of SAM (left panels) and RAM (right panels) from (a1,a2) Col-0 x *pCDT1a::GUS* and (a3,a4) *aip10-1* x *pCDT1a::GUS* seedlings at 5 DAG; black bars: 0.1mm. b) Graph with quantification of expression of *pCDT1a::GUS* in shoots of Col-0 and *aip10-1* seedlings at 5 DAG. The parameter for quantification of GUS expression is represented here as the measurement of GUS staining area of RAM and SAM with the ImageJ software with values given in arbitrary units. Bars represent means ± SD (standard deviation) and * significantly different from Col-0 with p ≤ 0.05 with Student's test. Data were obtained from ten biological replicates.

(a)

Col-0 x p*CDT1a::GUS*

aip10-1 x p*CDT1a::GUS*

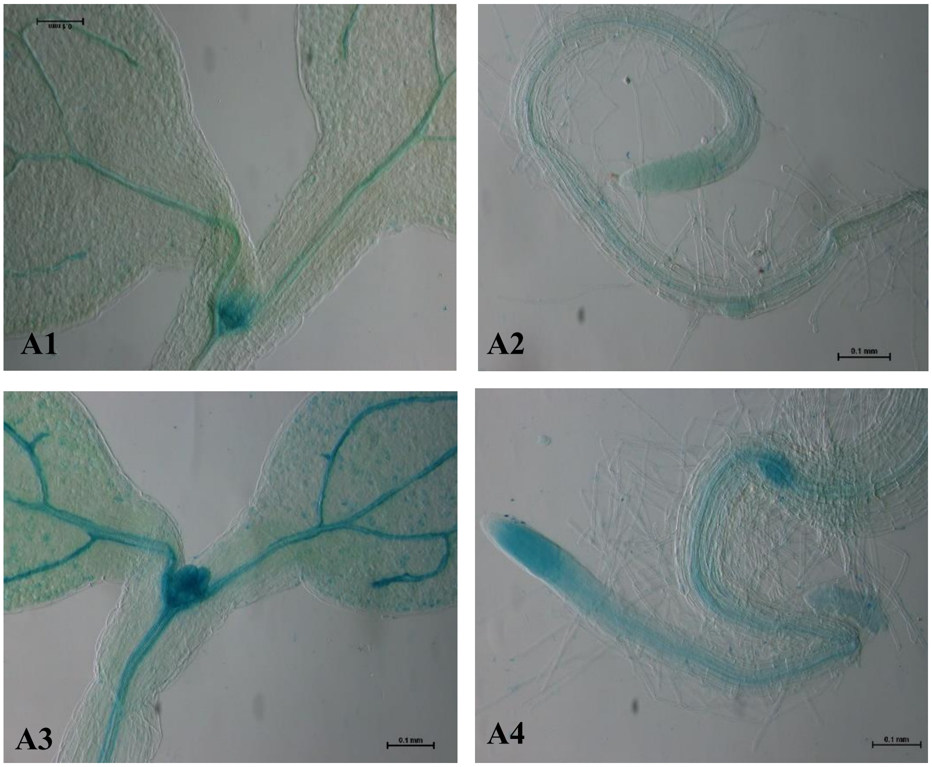

(b)

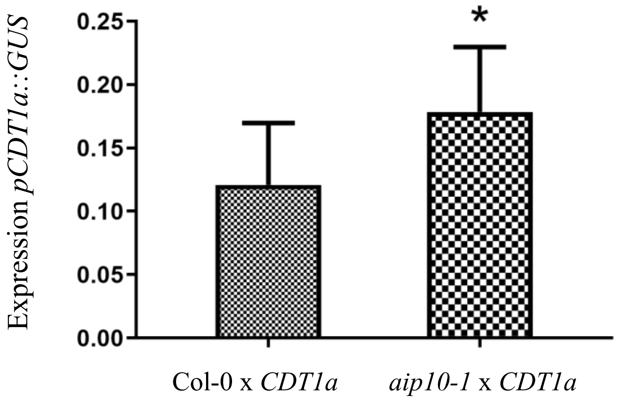

Figure S11. Average nuclear DNA content analyzed by flow cytometry. The graph shows the ploidy level measured by flow cytometry of the first pair of leaves and roots of Arabidopsis Col-0 and *aip10-1* plants at a) 14 DAG and b) 20 DAG. c) Bar of endoreduplication Index (EI). Bars represent means ± SD (standard deviation) and * significantly different from Col-0 with p≤ 0.05 with Student's test used in all statistical analyses. Data were obtained from three biological replicates.

(a)

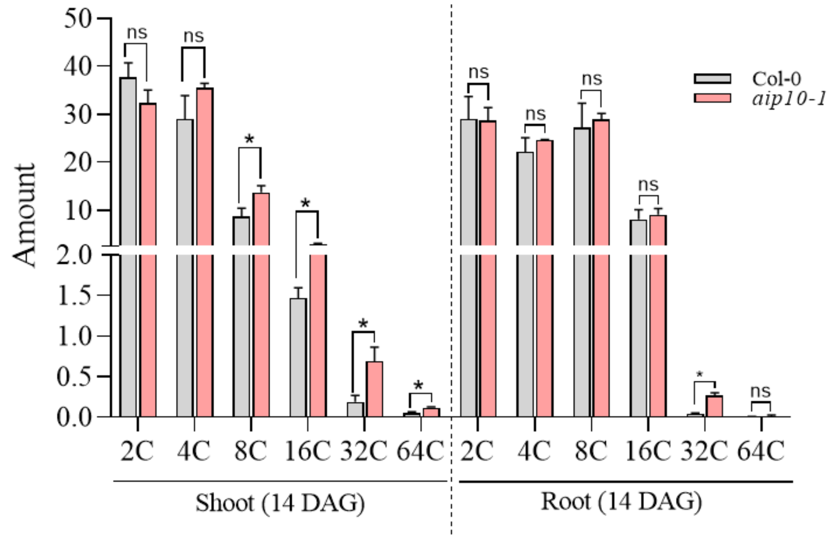

(b)

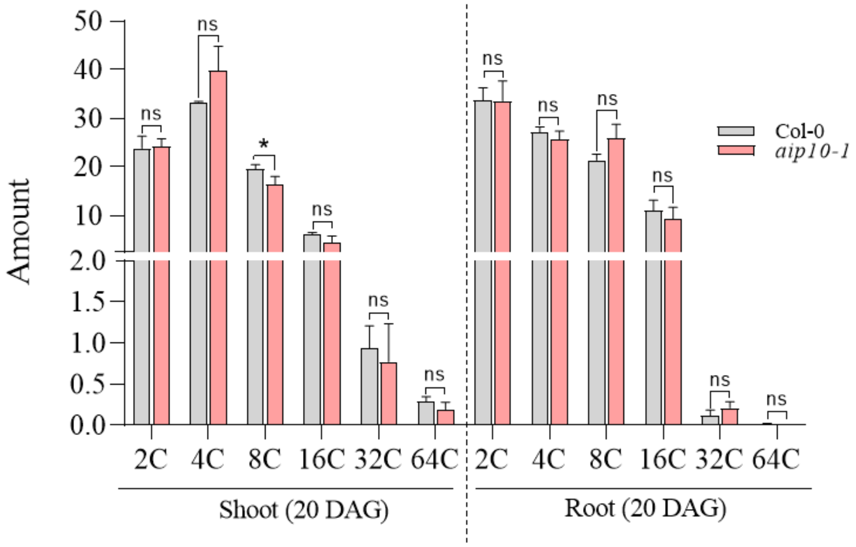

(c)

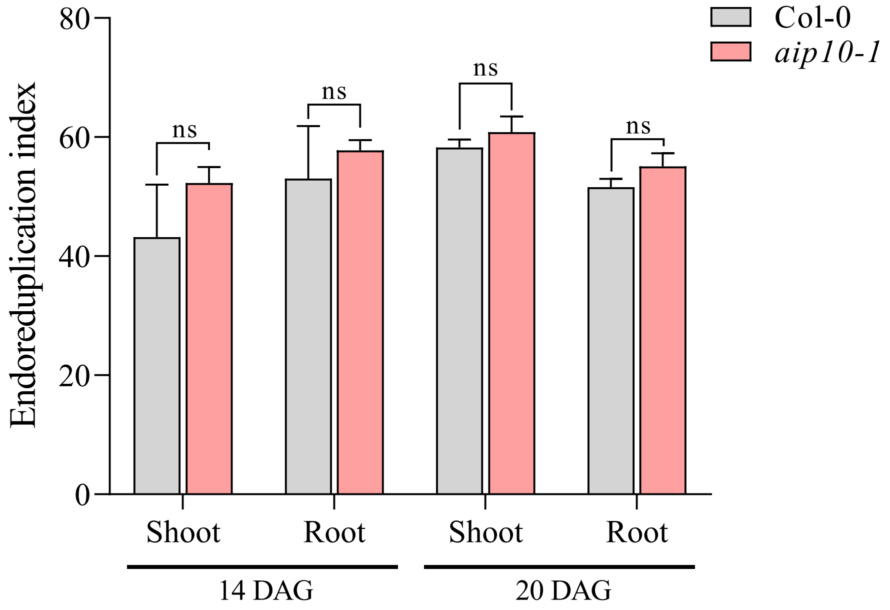
Col-0
aip10-1
Endoreduplication index
80
60
40
20
0
ns
ns
ns
ns
Shoot
Root
Shoot
Root
14 DAG
20 DAG

Figure S12. Comparative analysis of transcriptomic profiles between KIN10oe and lst8 datasets with *aip10-1* mutants. Comparison of *aip10-1* mutant transcriptomes in roots or shoots at 11 or 35 DAG with previously published datasets of lst8 mutants (TOR complex mutant, Forzani et al., 2019) and KIN10 overexpression conditions (KIN10oe/sugar starvation (Baena-González et al., 2007). Left: Venn diagrams represent the number of genes in each set and their overlap. Right: Scatterplots show the expression of DEGs ($\log_2$ fold change ≥ 0.5 or ≤ -0.5, $P < 0.05$), where genes upregulated in both datasets are marked in blue, those downregulated in red, and genes with discordant regulation are in gray. The red line drawn marks the diagonal where regulation would be identical between datasets. Comparative transcriptomic analysis of *aip10-1* mutants and KIN10oe/lst8 datasets in different tissues and time points: a) Roots at 11 DAG; b) Shoots at 11 DAG; c) Roots at 35 DAG; (d) Shoots at 35 DAG.

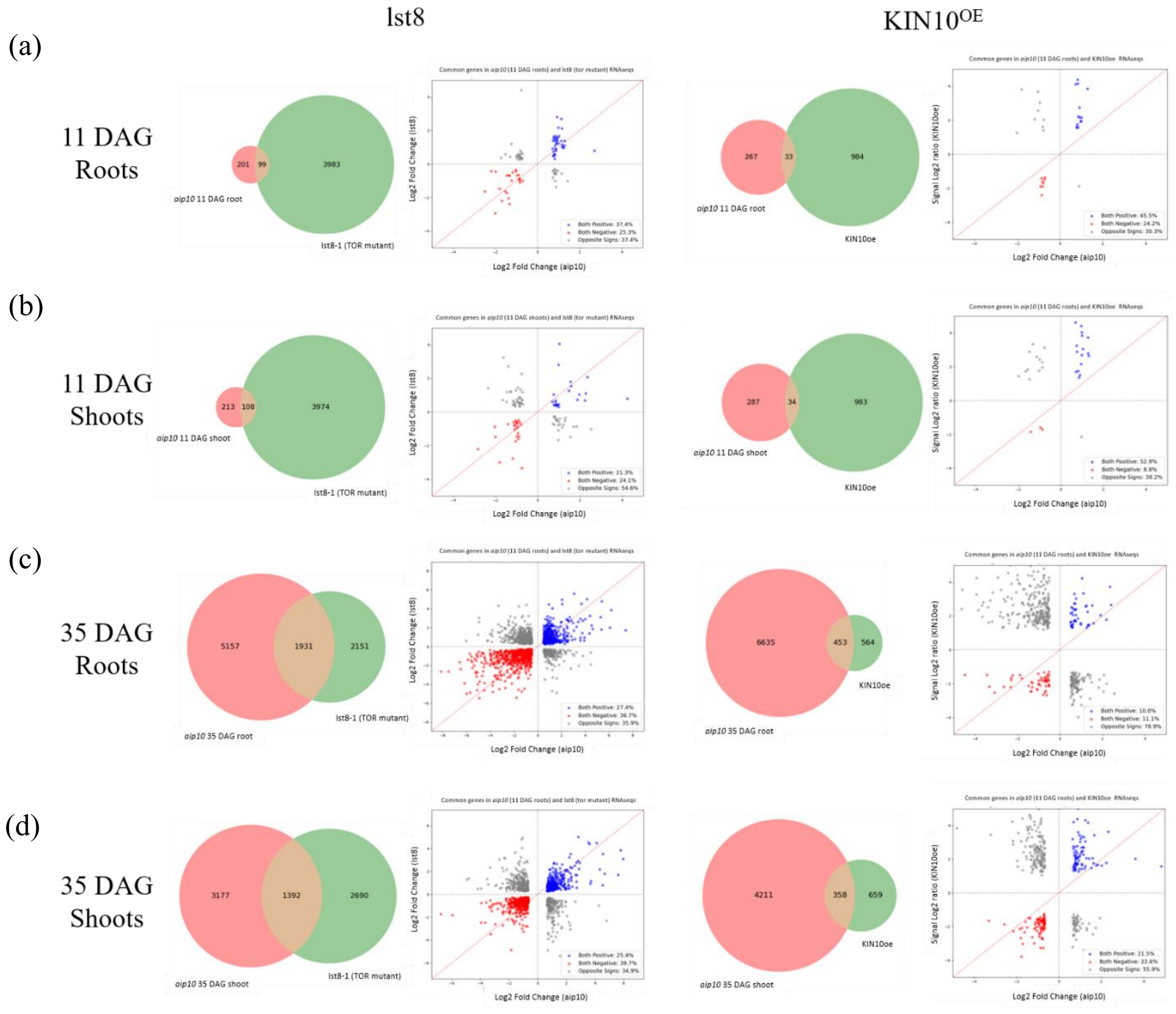

Figure S13. Comparative analysis of the expression profile of genes modulated by TOR inhibition differentially expressed in *aip10-1*. Comparison of *aip10-1* mutant transcriptomes in roots or shoots at 11 or 35 DAG with previously published dataset of TOR inhibition (iTOR, Dong et al., 2015). Left: Venn diagrams represent the number of genes in each set and their overlap. Right: Scatterplots show the expression of DEGs ($\log_2$ fold change ≥ 0.5 or ≤ -0.5, $P < 0.05$), where genes upregulated in both datasets are marked in blue, those downregulated in red, and genes with discordant regulation are in gray. The red line drawn marks the diagonal where regulation would be identical between datasets. Comparative transcriptomic analysis of *aip10-1* mutants and iTOR datasets in different tissues and time points: a) Roots at 11 DAG; b) Shoots at 11 DAG; c) Roots at 35 DAG; d) Shoots at 35 DAG.

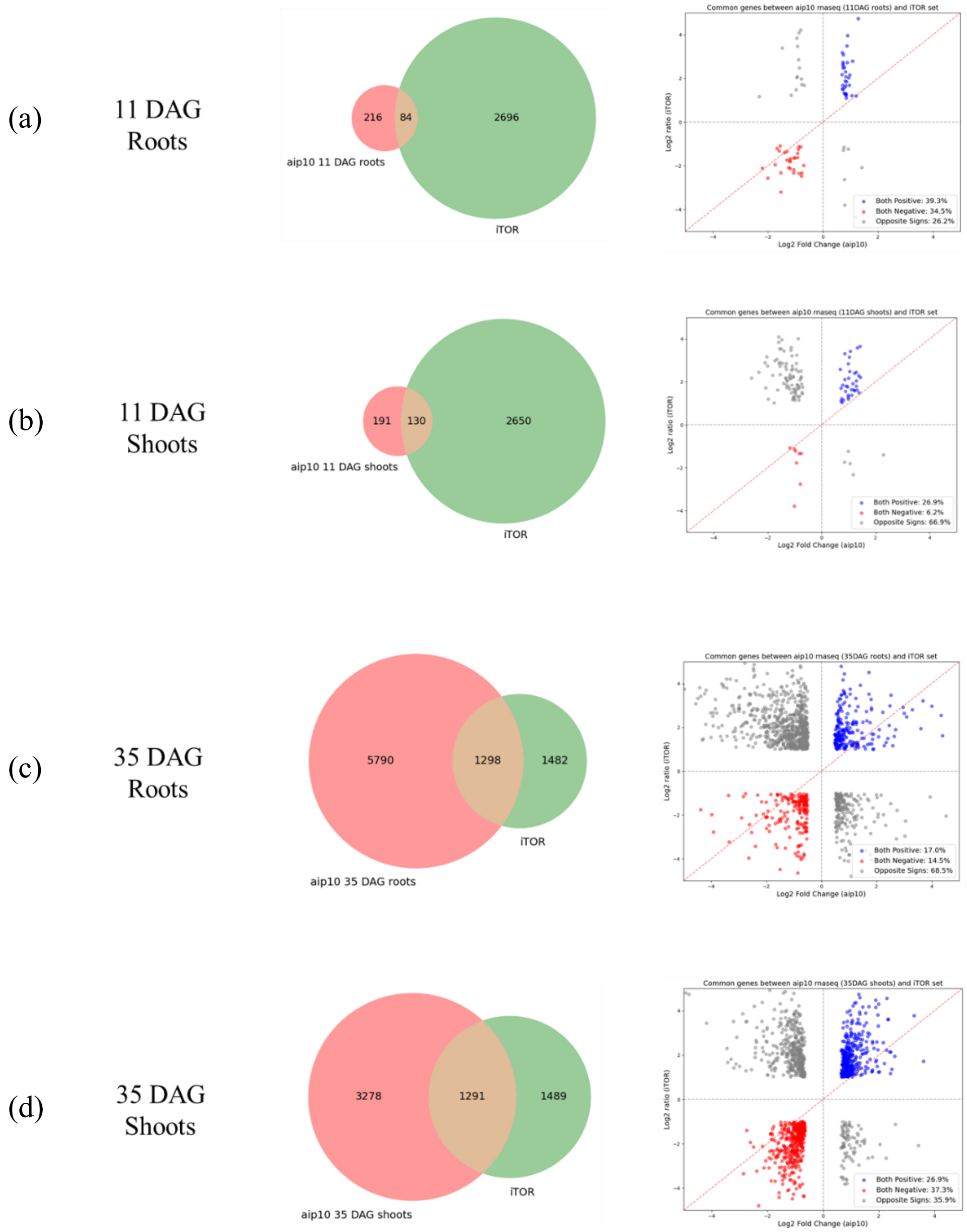

Figure S14. Comparative analysis of transcriptomic profiles between iTOR and lst8 datasets with *aip10-1* mutants. Comparison of *aip10-1* mutant transcriptomes in roots or shoots at 11 or 35 DAG with previously published datasets of lst8 mutants (TOR complex mutant, Forzani et al., 2019) and TOR inhibition (iTOR, Dong et al., 2015). Left: Venn diagrams represent the number of genes in each set and their overlap. Right: Scatterplots show the expression of DEGs ($\log_2$ fold change ≥ 0.5 or ≤ –0.5, $P < 0.05$), where genes upregulated in both datasets are marked in blue, those downregulated in red, and genes with discordant regulation are in gray. The red line drawn marks the diagonal where regulation would be identical between datasets. a) Direct comparison between lst8 and iTOR datasets; Comparative transcriptomic analysis of *aip10-1* mutants and lst8/iTOR datasets in different tissues and time points: b) Roots at 11 DAG; c) Shoots at 11 DAG; d) Roots at 35 DAG; e) Shoots at 35 DAG.

(a)

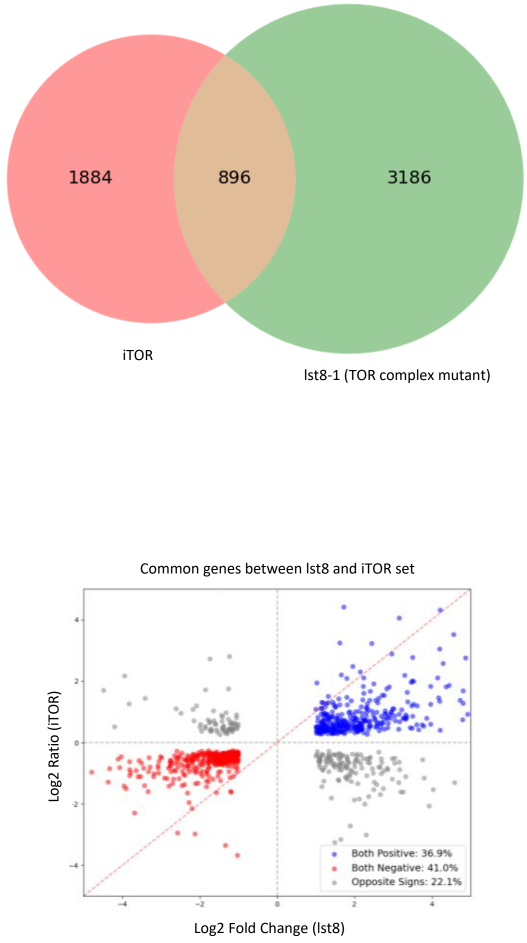

(b)

11 DAG Roots

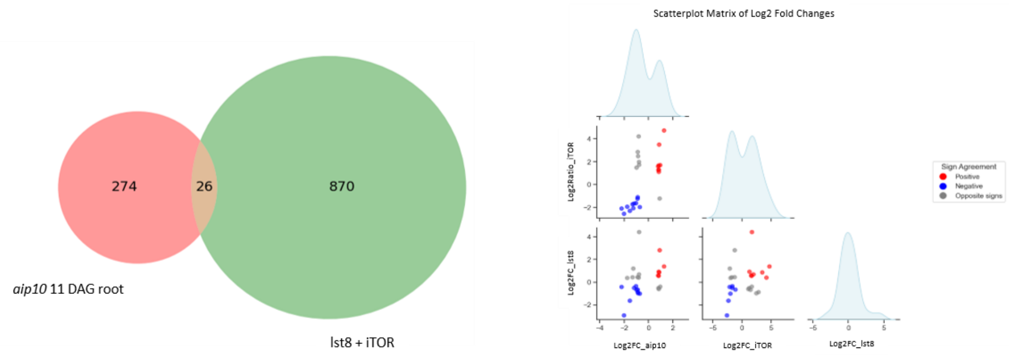

(c)

11 DAG Shoots

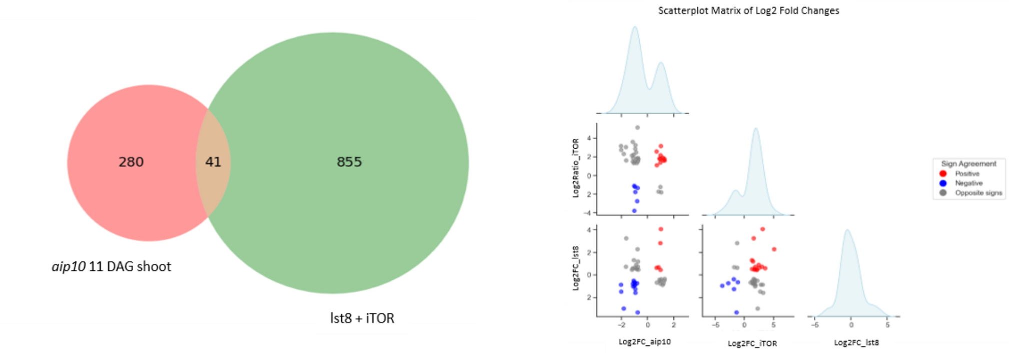

(d)

35 DAG Roots

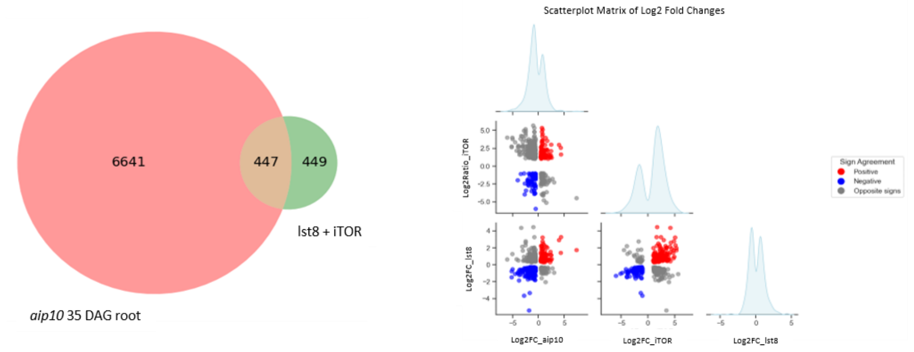

(e)

35 DAG Shoots

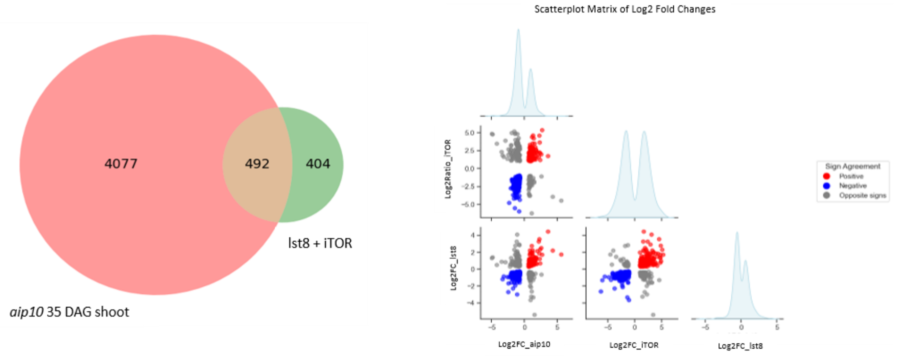

Figure S15. Analysis of total soluble sugar and starch content in source and sink tissues. Total soluble sugar and starch content were measured in Col-0, *aip10-1*, and *aip10-2* plants grown directly in soil under a 16 h/8 h photoperiod at 21 °C. Analyses were performed in source tissues (a,b) leaves at 20 DAG and (c,d) mature leaves at 35 DAG, as well as in sink tissues (e,f) green siliques (n = 4). Differences between groups were confirmed by one-way ANOVA (p≤ 0.05), and different letters indicate statistically different means according to Tukey's test at a 5% probability level.

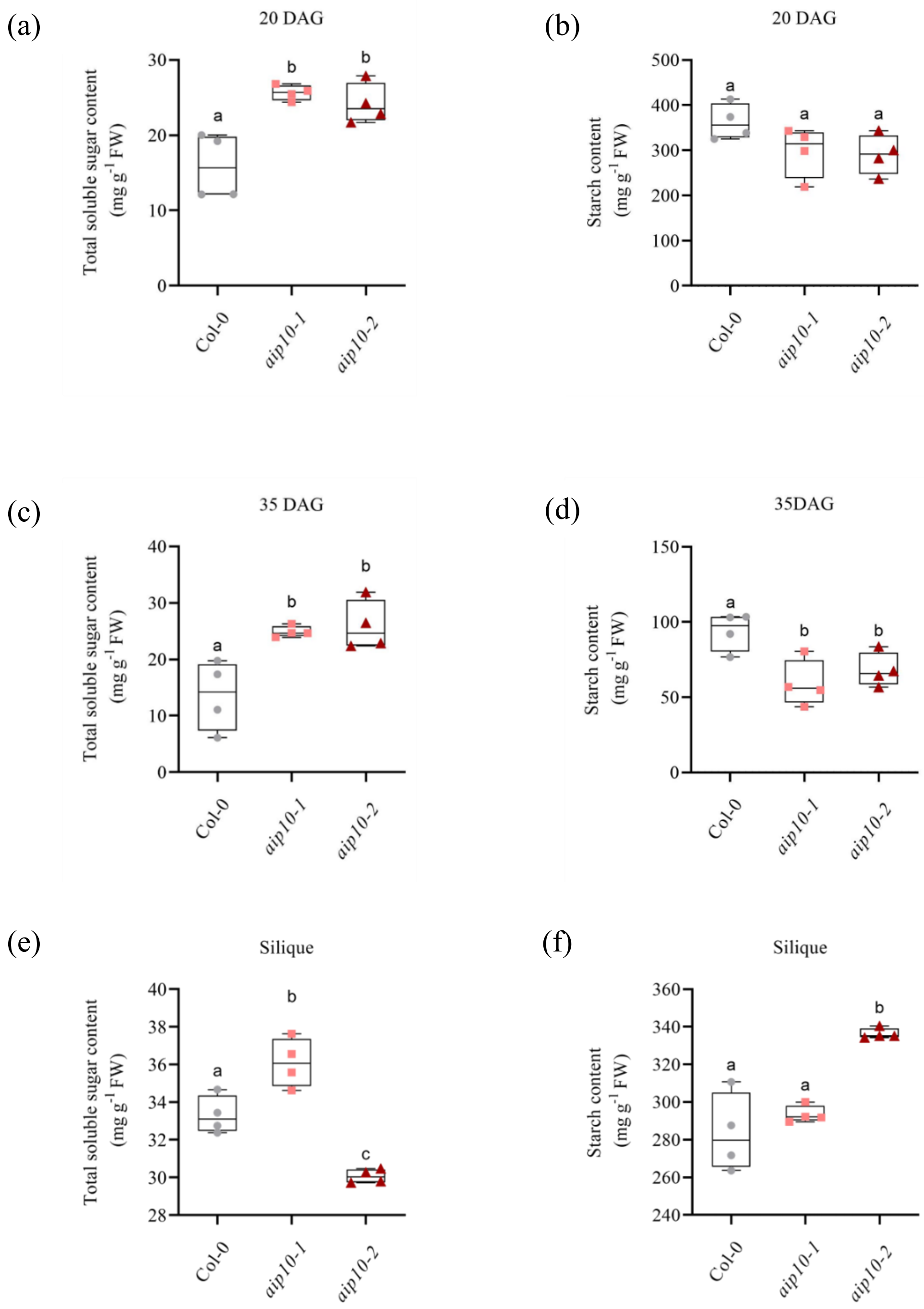

Figure S16. Loading chart for the first two main components applied to the ATR-FTIR dataset. (a) Col-0, *aip10-1* and *aip10-2* sheets from 35 DAG plants. (b) Col-0, *aip10-1* and *aip10-2* sheets from seeds. The spectra were obtained through the media of 4 individuals of each genotype.

(a)

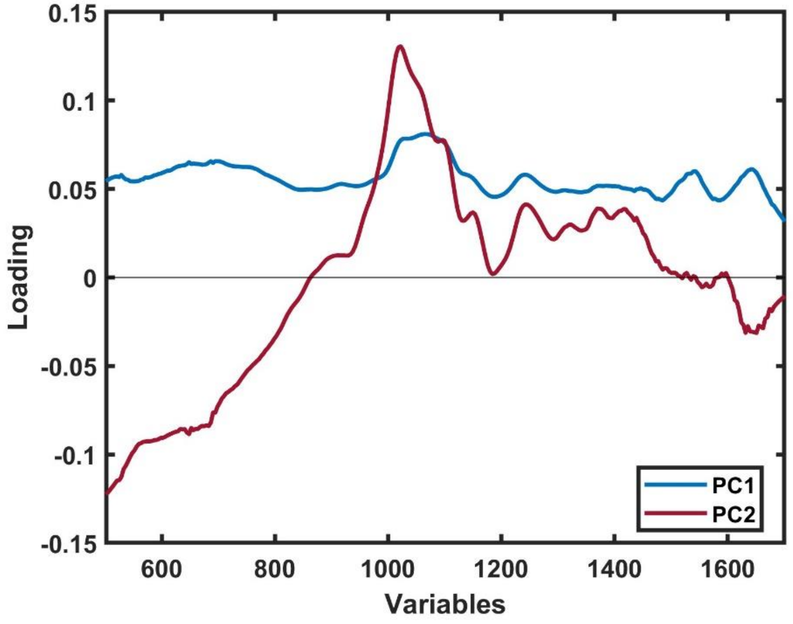

(b)

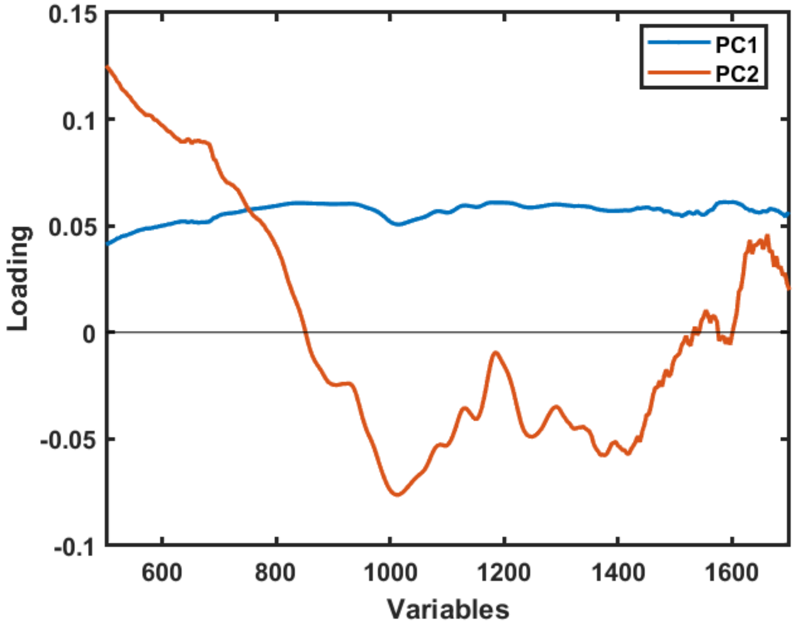

Figure S17. Metabolic analysis by ATR-FTIR spectrum of *aip10-1* and *aip10-2* plants at 20 DAG, compared with wild-type Col-0. a) Average spectra obtained by the FTIR technique in the region of 900–1700 cm^{-1} of Col-0 and *aip10-1* plants at 20 DAG. b) Average spectra obtained by the FTIR technique in the region of 900–1700 cm^{-1} of Col-0 and *aip10-2* plants at 20 DAG. c) Score plot for three principal components applied to the 20 DAG FTIR dataset. d) Loading Chart for the first two main components applied to the ATR-FTIR dataset. The experiment was carried out on 2 distinct leaves of 4 individuals of each genotype.

(a)

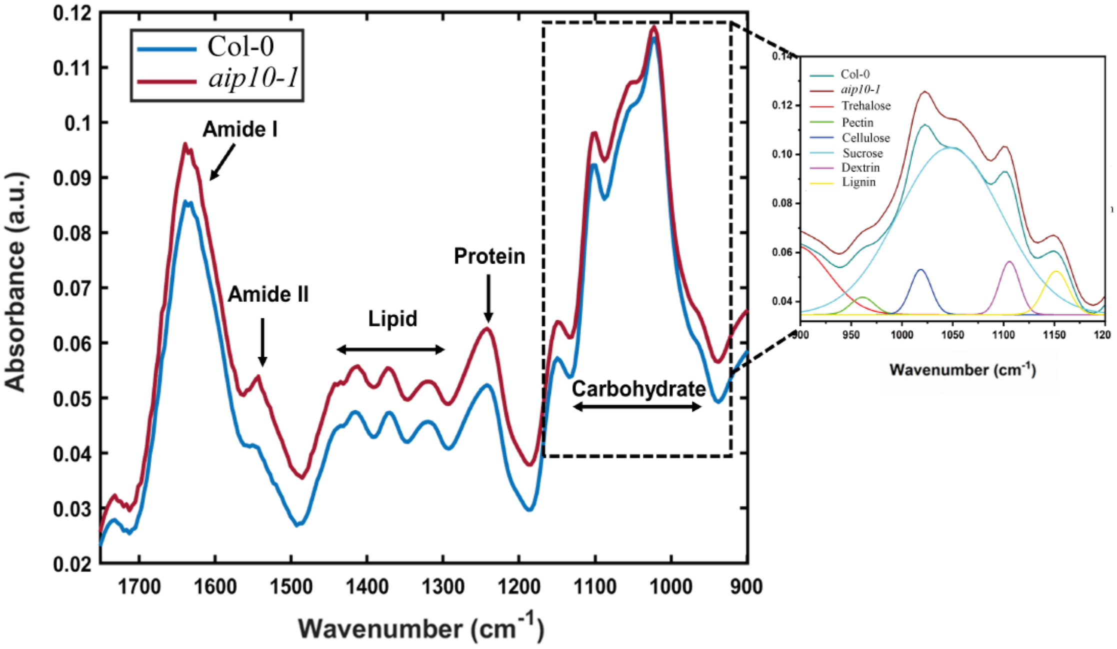

(b)

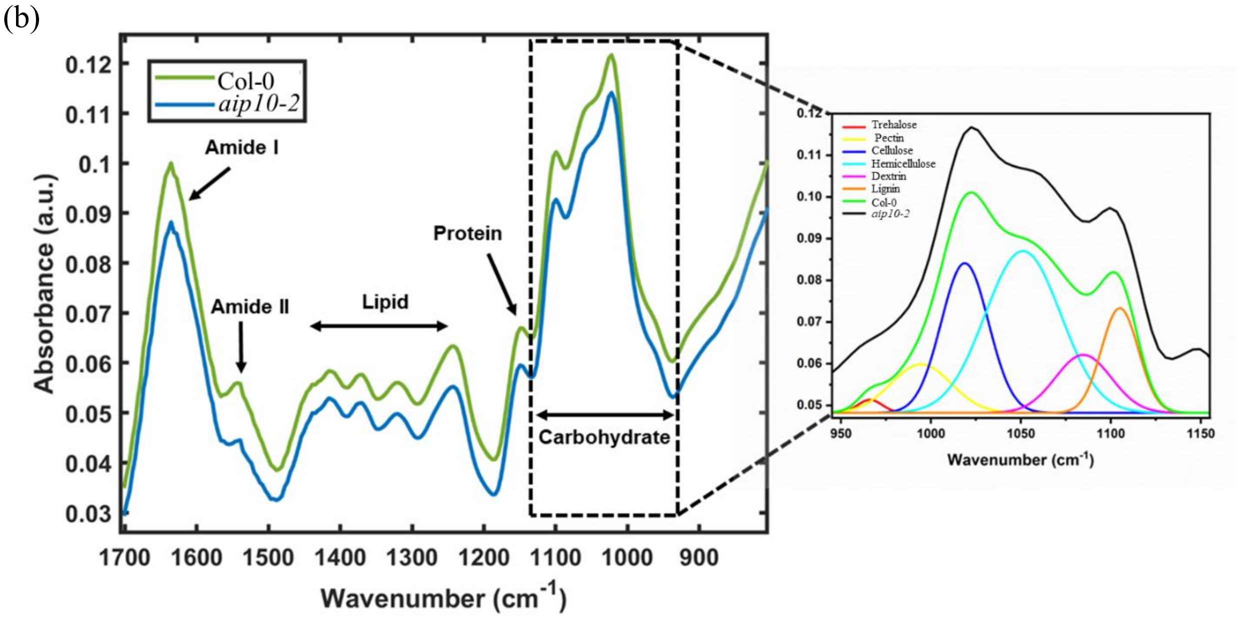

(c)

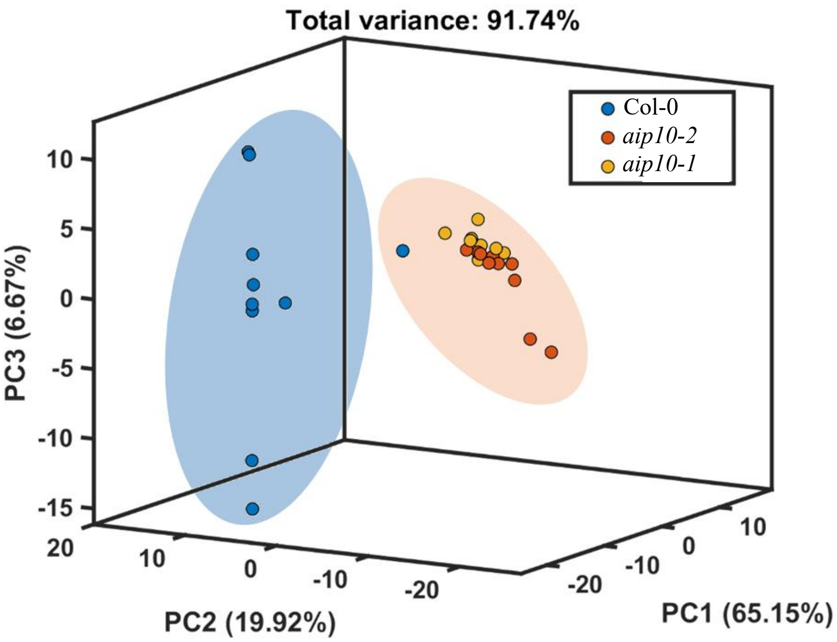
Total variance: 91.74%
Col-0
aip10-2
aip10-1
PC3 (6.67%)
10
5
0
-5
-10
-15
20
10
0
-10
-20
PC2 (19.92%)
-20
-10
0
10
PC1 (65.15%)

(d)

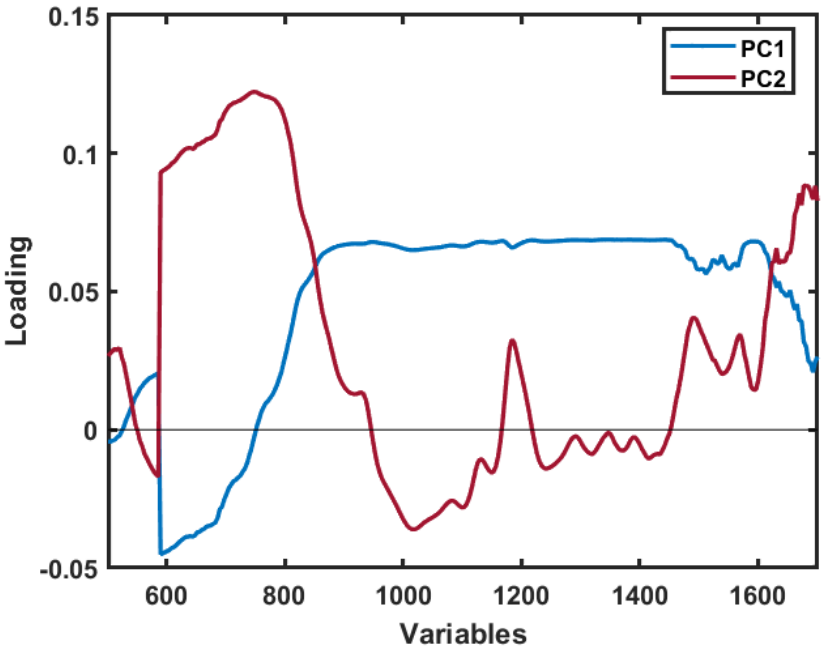
PC1
PC2
Loading
0.15
0.1
0.05
0
-0.05
600
800
1000
1200
1400
1600
Variables

Supporting tables

Table S1. Primers used in this study

Primer name	Primer Forward Sequence	Primer Reverse Sequence
**AIP10 Salk Lines:**		
T-DNA border primer (LB1.3)	ATTTTGCCGATTTCGGAAC	
SALK_022332	ACACTCGAAATTCGTGGTCTG	CTTTCGTTTTGATCTGATCCG
SALK_094618	AGCTTTCCTTTTCTCGGACAG	ATATTTTTCTTGGCTGCATGG
**Cloning:**		
KIN11	GGGGACAAGTTTGTACAAAAAAGCAGGCTTCACAA TGGATCATTCATCAAATAGATTTGGCA	GGGGACCACTTTGTACAAGAAAGCTGGGTCTAAAA CATTCAGATCACACGAAGC
**qRT-PCR:**		
AIP10	GGAGCTGAAGAACCCTAAGCTAGTT	GGACGAGACAAATCTACAAAAGAATAAA
AIP10.1	AAGAAGGTTAAAGGTTGCATG	TTTGGCGTTTTGGTTACGACTA
AIP10.2	GCTGGAGTTTGATTTACGGATTG	TCTGTTCTCTTGCTTCTCTCTTG
AIP10.3	GCTACCCTTTCTATTTCTGGTCT	GGTCACACCAGAGAAGAACGA
AIP10.4	CTGGTTGATCGGATCGAGAGATAGA	TCTGTTCTCTTGCTTCTCTCTTG
ABAP1	TCAGCCTTAAGAAGAGCTTGCA	ACCATAATTGAGAGCTGAGCTTAGTG
Cdt1a	AATCCGATCACGTCTTGAAGAAG	GAACCACGATCTCAAGAAAGCA
Cdt1b	AAATGTCGACTGCGAAACAG	AAGTGAAATGTCATGTGAAGTTGCTT
Cyclin B1;1	CCTCCATTCACTCTCAACAG	CCTGGCAGCTGTGGAATATG
Cyclin B1;2	TCAGTGCCTTGCTTATTGCTTCC	GTCGGGACTGTCAAATACCATTCG
QQS	GGTTCATTTTGCCTCACACTTCT	CCCATGATATGACCCTCATTTTG
LHCB1	TCAAGCCATCGTCACTGGTAAG	ACTGGATCGGCCAAATGGT
LHCB6	TTGCCATGTTGATCTTTTACTTTGA	CATCATGCCGTTTTCTCACAA
LZF1	CGAATCAAGGGTTGGGAAA	AGCGGCTAAAAGATAATTACAAATGC
PSAD2	GGTAAAAATGTTAGTCCCATTGAGGTT	CATCCGCAAGATTTTACATGTAGTG
DJC22	GGAGCAGGAACCCGTGATTA	ACACATTTGGATGATTTCTTTCGA
DJC23	AACCGATCAGTGTGGGTAGTGAGTT	GGATTTTATGGCTTCGTATTTACACTAA
DJC24	GCAGTTACGGTGGACGGAAT	GGACCAACTCGACACATCGA
NAD1	GACATTAGCCGAATCTGATCCAT	CAAGATAGAGACATAGAGAAGAAGAAGCA
GBSS1	GCGACGCCGTGAGAATATG	CTTCCCAACGCTTTCTTCAAA
STP1	GCAGCTCCAAAAAAGCAAGAA	TGTTGCCTTACGGGTTTAATCC
ASN1/DIN6	AACTTGTCGCCAGATCAAGG	GGAACACGTGCCTCTAGTCC
SEN1	CAGAGTCGGATCAGGAATGG	ATTTGACCGCTCTCACAACC
TPS11	TTCAACAGCCTATGGGACAATG	AAAATCTAACGCAAACCCTTTCAA
**Housekeeping genes:**		
GAPDH	TTGGTGACAACAGGTCAAGCA	AAACTTGTCGCTCAATGCAATC
UBI14	TCACTGGAAAGACCATTACTCTTGAA	AGCTGTTTTCCAGCGAAGATG

Table S2. AIP10 putative orthologs in other plant species

Gene ID	Isoforms	Scientific Name	Query Cover	Evalue	Per. Ident	Acc. Len
AT1G80940	AT1G80940.1	*Arabidopsis thaliana*	100%	9E-159	100.00%	213
	AT1G80940.2		71%	8E-109	98.03%	175
	AT1G80940.3		70%	7E-110	100.00%	151
	AT1G80940.4		61%	2E-91	96.15%	158
Araha.25042s0002	Araha.25042s0002.1	*Arabidopsis helleri*	100%	1E-139	92.52%	214
AL2G40440	AL2G40440.t1	*Arabidopsis lyrata*	95%	1E-131	93.14%	214
Aqcoe7G220600	Aqcoe7G220600.1	*A. coerulea*	91%	8E-69	59.61%	231
	Aqcoe7G220600.2		84%	8E-69	60.10%	231
	Aqcoe7G220600.3		89%	1E-64	72.14%	166
	Aqcoe7G220600.4		65%	1E-64	72.14%	166
AgateH2.02G147600	AgateH2.02G147600.1	*Agave tequilana var. Weber's Blue*	84%	3E-67	60.10%	209
	AgateH2.02G147600.2		60%	2E-38	52.86%	158
	AgateH2.02G147600.3		58%	5E-63	77.60%	172
AgateH2.06G036100	AgateH2.06G036100.1		89%	4E-67	56.48%	220
	AgateH2.06G036100.2		60%	4E-38	49.67%	158
	AgateH2.06G036100.3		89%	4E-67	56.48%	220
Alyli.0286s0053	Alyli.0286s0053.1	*Alyssum linifolium*	100%	7E-133	89.86%	216
Alyli.0018s0043	Alyli.0018s0043.1		95%	7E-130	90.87%	217
	Alyli.0018s0043.2		95%	7E-130	90.87%	217
evm_27.TU.AmTr_v1.0_scaffold00019.41	evm_27.model.AmTr_v1.0_scaffold00019.41	*Amborella trichopoda*	86%	7E-70	59.89%	199
Anaoc.0007s0942	Anaoc.0007s0942.1	*Anacardium occidentale*	92%	1E-74	64.85%	207
	Anaoc.0007s0942.2		62%	2E-46	61.48%	171
	Anaoc.0007s0942.3		67%	2E-64	72.11%	184
Anaoc.0004s1378	Anaoc.0004s1378.1		64%	5E-63	74.47%	178
Aco019699	Aco019699.1	*Ananas comosus*	90%	5E-64	57.89%	211
AndgeH2.04BG309800	AndgeH2.04BG309800.1	*Andropogon Gerardi*	89%	1E-70	61.31%	204
AndgeH2.04CG290300	AndgeH2.04CG290300.1		89%	1E-67	60.40%	207
arahy.Tifrunner.gnm1.ann1.HUQR60	arahy.Tifrunner.gnm1.ann1.HUQR60.1	*Arachis hypogaea*	92%	2E-71	56.65%	203
arahy.Tifrunner.gnm1.ann1.7IZ6IW	arahy.Tifrunner.gnm1.ann1.7IZ6IW.1		92%	2E-71	56.65%	203
evm.model.AsparagusV1_01.3431	evm.model.AsparagusV1_01.3431	*Asparagus officinalis*	89%	8E-69	53.21%	224
EL10Ac8g19398	EL10Ac8g19398	*Beta vulgaris*	90%	6E-80	63.13%	209
BPChr06G25282	BPChr06G25282	*Betula platyphylla*	80%	8E-72	65.70%	219
Bostr.20129s0414	Bostr.20129s0414.1	*Boechera stricta*	100%	9E-139	93.09%	216
	Bostr.20129s0414.2		100%	9E-139	93.09%	216
Barbu.4G085600	Barbu.4G085600.1	*Brachypodium arbuscula*	89%	9E-72	59.52%	214
BdiBd30-1.3G0702800	BdiBd30-1.3G0702800.1	*Brachypodium distachyon*	89%	3E-70	59.05%	214
Bradi3g53940	Bradi3g53940.1		89%	4E-69	58.57%	214
Bmexi.04PG339700	Bmexi.04PG339700.1	*Brachypodium mexicanum*	86%	2E-68	57.48%	214
	Bmexi.04PG339700.2		86%	2E-68	57.48%	214
	Bmexi.04PG339700.3		86%	2E-68	57.48%	214
	Bmexi.04PG339700.4		33%	7E-38	84.72%	128
	Bmexi.04PG339700.5		63%	3E-68	76.98%	196
Bmexi.04UG312100	Bmexi.04UG312100.1		89%	7E-68	58.10%	217
	Bmexi.04UG312100.2		89%	5E-70	58.94%	214
	Bmexi.04UG312100.3		89%	5E-70	58.94%	214
Brahy.D03G0743300	Brahy.D03G0743300.1	*Brachypodium hybridum*	84%	2E-67	59.69%	214
Brahy.S04G0082600	Brahy.S04G0082600.1		89%	2E-70	59.42%	214
Brasyl.4G084800	Brasyl.4G084800.1	*Brachypodium sylvaticum*	89%	1E-71	59.05%	214
Brast04G077900	Brast04G077900.1	*Brachypodium stacei*	89%	2E-70	59.42%	214
BcaB02g07237	BcaB02g07237	*Brassica Carinata*	100%	6E-128	87.73%	220
BcaNung04166	BcaNung04166		100%	7E-128	86.30%	217
BcaC08g47533	BcaC08g47533		95%	1E-122	82.79%	224
BcaB02g08711	BcaB02g08711		100%	4E-120	83.48%	224
A07p26960	A07p26960	*Brassica napus*	100%	1E-128	86.30%	219
C06p29630	C06p29630		100%	2E-128	86.76%	218
A07p44750	A07p44750		95%	3E-121	85.45%	223
C06p53570	C06p53570		97%	2E-120	83.49%	220
BolC6t38128H	BolC6t38128H	*Brassica oleracea*	100%	1E-128	87.56%	216
BolC6t40373H	BolC6t40373H		95%	1E-122	82.79%	225
BraA07t30340Z	BraA07t30340Z	*Brassica rapa*	100%	1E-128	86.30%	219
BraA07t31983Z	BraA07t31983Z		95%	3E-120	84.98%	223
Camar.0320s0022	Camar.0320s0022.1	*Cakile maritima*	100%	2E-123	84.68%	220
Camar.0744s0014	Camar.0744s0014.1		97%	3E-121	84.11%	209
Cagra.1725s0048	Cagra.1725s0048.1	*Capsella grandiflora*	100%	6E-139	93.09%	216
	Cagra.1725s0048.2		100%	6E-139	93.09%	216
evm.TU.contig_28380	evm.TU.contig_28380.1	*Carica papaya*	57%	3E-56	75.41%	151
Cocit.G1959	Cocit.G1959.1	*Carya illinoinensis*	93%	1E-71	61.19%	208
Cocit.F2186	Cocit.F2186.1		90%	3E-69	60.91%	216
Caden.05G119500	Caden.05G119500.1	*Castanea dentata*	100%	3E-87	62.04%	220
	Caden.05G119500.2		81%	1E-75	66.47%	177
	Caden.05G119500.3		81%	1E-75	66.47%	177

CmMahoganyH2.05G084100	CmMahoganyH2.05G084100.1	*Castanea mollissima Mahogany*	100%	3E-87	62.04%	220
	CmMahoganyH2.05G084100.2		81%	1E-75	66.47%	177
Caamp.0006s0694	Caamp.0006s0694.1	*Caulanthus amplexicaulis*	100%	7E-128	87.10%	212
Caamp.0064s0004	Caamp.0064s0004.1		99%	8E-128	87.04%	216
CepurGG1.5G004300	CepurGG1.5G004300	*Ceratodon purpureus GG1*	61%	8E-59	66.91%	338
CepurGG1.8G136700	CepurGG1.8G136700		61%	9E-56	65.22%	216
Ceric.33G057400	Ceric.33G057400.1	*Ceratopteris richardii*	87%	2E-60	53.23%	235
	Ceric.33G057400.2		62%	9E-61	67.67%	165
Ceric.22G068100	Ceric.22G068100.1		61%	4E-58	68.18%	233
CecanH2.7G287200	CecanH2.7G287200.1	*Cercis canadenses*	96%	2E-89	63.81%	212
	CecanH2.7G287200.2		66%	2E-54	63.83%	148
Chala.05G301900	Chala.05G301900.1	*Chasmanthium laxum*	89%	5E-67	60.00%	209
Ca_17536	Ca_17536	*Cicer arietinum*	92%	6E-87	64.97%	199
CKAN_00379300	CKAN_00379300	*Cinnamomum kanehirae*	89%	2E-68	60.62%	206
Ciclev10026461m.g	Ciclev10026461m.g	*Citrus clementina*	97%	2E-75	61.61%	216
orange1.1g028560mg	orange1.1g028560mg	*Citrus sinensis*	97%	1E-73	60.48%	207
orange1.1g027963mg	orange1.1g027963m		97%	2E-70	61.61%	216
Clevi.0013s1301	Clevi.0013s1301.1	*Cleome violacea*	95%	3E-103	78.33%	213
evm.TU.Scaffold_633.236	evm.model.Scaffold_633.236	*Coffea arabica*	92%	8E-74	62.63%	208
evm.TU.Scaffold_352.635	evm.model.Scaffold_352.635		92%	5E-75	63.64%	208
Cc04_g05300	Cc04_g05300	*Coffea canephora*	92%	5E-75	63.64%	208
CamerWinkler.07G149100	CamerWinkler.07G149100.1	*Corylus americana*	96%	6E-88	66.67%	213
	CamerWinkler.07G149100.2		75%	2E-74	70.00%	179
Crahi.0517s0015	Crahi.0517s0015.1	*Crambe hispanica*	95%	7E-119	86.47%	211
Cucsa.106110	Cucsa.106110.1	*Cucumis sativus*	64%	3E-74	73.38%	141
DCAR_021374	DCAR_021374	*Daucus carota*	91%	6E-87	65.46%	206
Desop.0243s1251	Desop.0243s1251.1	*Descurainia sophioides*	95%	2E-128	90.34%	217
Dioal.08G104800	Dioal.08G104800.1	*Dioscorea alata*	86%	6E-77	63.27%	210
Dicom.09G018300	Dicom.09G018300.1	*Diphasiastrum complanatum*	61%	2E-55	66.92%	209
	Dicom.09G018300.2		61%	2E-55	66.92%	209
Dicom.09G018600	Dicom.09G018600.1		61%	2E-55	66.92%	209
Dicom.05G016600	Dicom.05G016600.1		63%	3E-49	60.74%	211
	Dicom.05G016600.2		63%	6E-50	60.74%	149
Distr.0003s0768	Distr.0003s0768.1	*Diptychocarpus strictus*	94%	8E-124	87.25%	217
ELECO.r07.2BG0204120	ELECO.r07.2BG0204120.1	*Eleusine coracana*	89%	2E-70	60.19%	209
ELECO.r07.2AG0148740	ELECO.r07.2AG0148740.1		89%	2E-70	59.51%	209
Eruve.1455s0006	Eruve.1455s0006.1	*Eruca vesicaria*	97%	6E-127	87.04%	222
Eruve.4109s0007	Eruve.4109s0007.1		97%	4E-126	86.57%	222
	Eruve.4109s0007.2		75%	4E-100	92.59%	165
Eruve.0031s0115	Eruve.0031s0115.1		100%	8E-121	84.68%	222
	Eruve.0031s0115.2		100%	8E-121	84.68%	222
Eruve.1455s0007	Eruve.1455s0007.1		86%	2E-111	86.46%	198
	Eruve.1455s0007.2		62%	2E-84	94.81%	142
Eucgr.F03985	Eucgr.F03985	*Eucalyptus grandis*	92%	2E-77	59.70%	212
Eucgr.J03130	Eucgr.J03130		93%	6E-72	62.56%	208
Eusyr.0118s1180	Eusyr.0118s1180.1	*Euclidium syriacum*	95%	8E-126	88.89%	217
Thhalv10019123m	Thhalv10019123m	*Eutrema salsugineum*	95%	5E-131	89.42%	217
Thhalv10019121m	Thhalv10019121m		95%	5E-131	89.42%	217
FvH4_6g28270	FvH4_6g28270.t1	*Fragaria vesca*	80%	4E-72	67.43%	209
	FvH4_6g28270.t2		92%	2E-82	66.16%	211
Glyma.08G220400	Glyma.08G220400.1	*Glycine max*	95%	3E-91	66.67%	210
Glyma.07G021400	Glyma.07G021400.1		100%	5E-90	64.19%	214
Gohir.A11G075200	Gohir.A11G075200.1	*Gossypium hirsutum*	91%	5E-88	69.39%	214
Gohir.D11G079900	Gohir.D11G079900.1		81%	1E-82	73.14%	195
Gorai.007G085500	Gorai.007G085500.1	*Gossypium raimondii*	87%	1E-88	72.34%	214
	Gorai.007G085500.2		81%	1E-82	73.14%	195
	Gorai.007G085500.3		87%	1E-68	61.70%	194
HanXRQChr04g0100021	HanXRQChr04g0100021	*Helianthus annuus*	91%	1E-86	69.23%	211
HanXRQChr09g0274181	HanXRQChr09g0274181		92%	2E-83	65.67%	205
Hyque.02G002700	Hyque.02G002700.1	*Hydrangea quercifolia*	97%	6E-95	64.32%	210
	Hyque.02G002700.2		97%	5E-95	64.62%	209
HORVU0Hr1G040430	HORVU0Hr1G040430.1	*Hordem vulgare*	88%	1E-65	57.94%	351
	HORVU0Hr1G040430.4		88%	7E-66	57.94%	345
Ibeam.0002s0036	Ibeam.0002s0036.1	*Iberis amara*	95%	4E-103	80.95%	214
	Ibeam.0002s0036.2		95%	2E-99	80.00%	209
Isati.5657s0005	Isati.5657s0005.1	*Isatis tinctoria*	99%	7E-130	87.91%	214
Joasc.11G051500	Joasc.11G051500.1	*Joinvillea ascendens*	90%	9E-76	61.39%	204
	Joasc.11G051500.2		90%	9E-76	61.39%	204
	Joasc.11G051500.3		63%	8E-72	76.98%	158
	Joasc.11G051500.4		33%	1E-37	84.72%	96
Kaladp0335s0001	Kaladp0335s0001.1	*K. fedtschenkoi*	87%	4E-76	63.16%	233
Kaladp0037s0373	Kaladp0037s0373.1		81%	5E-74	66.29%	247
	Kaladp0037s0373.2		61%	7E-73	77.04%	180
	Kaladp0037s0373.3		62%	2E-71	76.47%	243
	Kaladp0037s0373.4		61%	7E-73	77.04%	180
	Kaladp0037s0373.5		61%	7E-73	77.04%	180

Lsat_1_v5_gn_1_30801	Lsat_1_v5_gn_1_30801.1	*Lactuca sativa*	92%	1E-89	64.79%	213
	Lsat_1_v5_gn_1_30801.2		95%	4E-91	66.51%	212
Lsat_1_v5_gn_4_85061	Lsat_1_v5_gn_4_85061.1		97%	1E-87	61.19%	216
Lcu.2RBY.4g001240	Lcu.2RBY.4g001240.1	*Lens culinaris*	93%	3E-82	64.50%	210
Lesat.0180s0006	Lesat.0180s0006.1	*Lepidium sativum*	100%	4E-130	87.33%	219
	Lesat.0180s0006.2		100%	1E-132	88.94%	215
	Lesat.0180s0006.3		100%	1E-132	88.94%	215
Lesat.0061s0110	Lesat.0061s0110.1		95%	1E-125	87.74%	220
Liphi.05G091000	Liphi.05G091000.1	*Lindenbergia philippensis*	95%	4E-81	63.85%	215
	Liphi.05G091000.2		71%	3E-68	73.03%	164
Lus10041624	Lus10041624	*Linum usitatissimum*	86%	1E-78	64.80%	236
Lus10024095	Lus10024095		86%	3E-77	63.27%	321
LitulAlt.12G037200	LitulAlt.12G037200	*Liriodendron tulipifera*	91%	4E-75	61.46%	215
Lj3g0027435	Lj3g0027435.1	*Lotus japonicus*	96%	7E-87	61.29%	215
Luann.0047s0143	Luann.0047s0143.1	*Lunaria annua*	95%	7E-130	90.34%	216
Luann.1171s0004	Luann.1171s0004.1		62%	1E-76	89.47%	152
Lalb_Chr01g0011991	Lalb_Chr01g0011991	*Lupinus albus*	93%	1E-81	61.81%	206
Lalb_Chr25g0278731	Lalb_Chr25g0278731		89%	6E-81	65.10%	206
Mamar.0107s0005	Mamar.0107s0005.1	*Malcolmia maritima*	100%	6E-135	92.49%	210
Manes.11G069200	Manes.11G069200	*Manihot esculenta*	98%	7E-90	68.25%	220
Manes.04G100900	Manes.04G100900		90%	1E-82	67.01%	202
Mapoly0128s0009	Mapoly0128s0009	*Marchantia polymorpha*	61%	1E-62	69.23%	243
Medtr4g007700	Medtr4g007700.1	*Medicago truncatula*	93%	7E-88	64.93%	209
	Medtr4g007700.2		81%	5E-78	62.76%	195
MgTOL.I0689	MgTOL.I0689.1	*Mimulus guttatus*	91%	1E-82	60.09%	223
Misin07G522700	Misin07G522700	*Miscanthus sinensis*	89%	3E-66	60.30%	321
Ma03_g04600	Ma03_g04600	*Musa acuminata*	86%	6E-70	57.87%	204
Myper.0013s0857	Myper.0013s0857.1	*Myagrum perfoliatum*	99%	1E-130	88.37%	214
NbS00012287g0005	NbS00012287g0005	*Nicotiana benthamiana*	90%	4,00E-87	63.92%	209
NbS00042944g0008	NbS00042944g0008		77%	1,00E-78	66.87%	184
Nycol.C02206	Nycol.C02206.1	*Nymphaea colorata*	91%	3,00E-82	60.58%	222
	Nycol.C02206.2		65%	6,00E-47	54.90%	178
Nitab4.5_0004738g0090	Nitab4.5_0004738g0090	*Nicotiana tabacum*	94%	9,00E-89	63.37%	215
Nitab4.5_0002700g0060	Nitab4.5_0002700g0060		90%	3,00E-88	65.80%	208
Oeu051555	Oeu051555.1	*Olea europaea*	74%	5E-63	67.70%	156
Oeu003125	Oeu003125.1		65%	8E-62	73.43%	265
Oropetium_20150105_04478	Oropetium_20150105_04478A	*Oropetium thomaeum*	81%	4E-63	61.17%	576
LOC_Os02g55080	LOC_Os02g55080	*Oryza sativa*	86%	5E-70	60.70%	210
Pahal.1G428900	Pahal.1G428900.1	*Panicum hallii*	89%	2E-70	60.29%	207
PhHAL.1G419700	PhHAL.1G419700.1	*Panicum hallii HAL*	89%	2E-70	60.29%	207
Pavir.1NG509700	Pavir.1NG509700.2	*Panicum virgatum*	89%	1E-69	59.90%	207
Pavir.1KG533800	Pavir.1KG533800.1		89%	2E-68	58.82%	207
	Pavir.1KG533800.2		59%	2E-37	54.74%	183
Pavag04G304200	Pavag04G304200.1	*Paspalum vaginatum*	89%	8E-67	59.90%	207
Phala.02G314500	Phala.02G314500.1	*Pharus latifolius*	89%	5E-69	58.25%	210
	Phala.02G314500.2		61%	5,00E-40	52.78%	153
Phacu.CVR.008G317800	Phacu.CVR.008G317800.1	*Phaseolus acutifolius*	95%	1,00E-90	64.56%	209
Phvul.008G253200	Phvul.008G253200.1	*Phaseolus vulgaris*	95%	3,00E-91	65.05%	209
Pp3c16_25700	Pp3c16_25700V3.1	*Physcomitrium patens*	61%	9,00E-62	66.91%	339
	Pp3c16_25700V3.2		61%	9,00E-62	66.91%	339
Pp3c25_9780	Pp3c25_9780V3.1		61%	3,00E-60	66.18%	338
	Pp3c25_9780V3.2		61%	3,00E-60	66.18%	339
Psat7g262200	Psat7g262200	*Pisum sativum*	93%	6E-88	64.88%	210
Ptrif.0007s1735	Ptrif.0007s1735.1	*Poncirus trifoliata*	97%	1E-75	61.61%	216
	Ptrif.0007s1735.2		97%	7E-74	60.48%	207
	Ptrif.0007s1735.3		66%	6E-66	75.69%	145
Podel.03G199500	Podel.03G199500.1	*Populus deltoides*	95%	5E-88	68.29%	220
Podel.01G043400	Podel.01G043400.1		95%	1E-86	66.83%	221
	Podel.01G043400.2		90%	1E-75	63.21%	207
	Podel.01G043400.3		63%	3E-72	80.00%	152
Poman.03G163300	Poman.03G163300.1	*Populus nigra x maximowiczii*	95%	1E-88	68.78%	221
	Poman.03G163300.2		90%	7E-78	65.28%	207
	Poman.03G163300.3		63%	2E-72	80.74%	152
Poman.01G035900	Poman.01G035900.1		91%	5E-87	70.26%	214
	Poman.01G035900.2		90%	7E-78	65.28%	207

Table S3. ABAP1 putative orthologs in other plant species

Gene ID	Isoforms	Scientific Name	Query Cover	E value	Per. Ident	Acc. Len
AT5G13060	AT5G13060.1	Arabidopsis thaliana	100%	0	100%	737
Sobic.009G129600	Sobic.009G129600.1	Sorghum bicolor	88%	0.0	60.79%	745
Sspon.07G0008510-2C	Sspon.07G0008510-2C	Saccharum spontaneum	79%	0.0	58.38%	622
Zm00001eb287530	Zm00001eb287530	Zea mays B73	88%	0.0	59.69%	724
Zm00001eb349910	Zm00001eb349910		87%	0.0	60.74%	748
OsR498G0510791000	OsR498G0510791000.01	Oryza sativa	89%	0.0	60.76%	745
TraesCS1A03G0645500	TraesCS1A03G0645500	Triticum aestivum	88%	0.0	60.37%	730
TraesCS1B03G0731200	TraesCS1B03G0731200		89%	0.0	60.15%	742
TraesCS1D03G0602500	TraesCS1D03G0602500		89%	0.0	60.15%	776
Horvu_MOREX_1H01G392500	Horvu_MOREX_1H01G392500	Hordeum vulgare	89%	0.0	59.67%	742
Ma09_g16490	Ma09_g16490.1	Musa acuminata	89%	0.0	61.93%	726
Cc01_g19440	Cc01_g19440.1	Coffea canephora	95%	0.0	60.85%	712
evm.TU.Scaffold_557.287	evm.model.Scaffold_557.287	Coffea arabica	95%	0.0	58.96%	692
evm.TU.Scaffold_2016.221	evm.model.Scaffold_2016.221		95%	0.0	57.94%	717
GSVIVG01011129001	GSVIVG01011129001	Vitis vinifera	93%	0.0	59.43%	713
GSVIVG01017925001	GSVIVG01017925001		89%	0.0	63.47%	705
PGSC0003DMG400012880	PGSC0003DMT400033530	Solanum tuberosum	95%	0.0	59.83%	708
PGSC0003DMG401020126	PGSC0003DMT400051860		95%	0.0	59.86%	709
Solyc09G001948	Solyc09G001948.1	Solanum lycopersicum	95%	0.0	59.92%	708
Solyc06G002747	Solyc06T002747.1		95%	0.0	50.86%	734
	Solyc06T002747.2		95%	0.0	51.88%	720
Manes.03G181400	Manes.03G181400.1	Manihot esculenta	87%	0.0	63.79%	708
Manes.05G076800	Manes.05G076800.4		91%	0.0	62.61%	692
Manes.15G026400	Manes.15G026400.3		94%	0.0	61.17%	707
A03p09430	A03p09430	Brassica napus	92%	0.0	59.33%	716
C09p56950	C09p56950		88%	0.0	60.09%	1266
C03p11010	C03p11010		82%	0.0	50.49%	554
A10p25140	A10p25140		96%	0.0	85.09%	707
C09p64040	C09p64040		96%	0.0	79.18%	755
C02p05870	C02p05870		99%	0.0	81.66%	729
A02p01660	A02p01660		96%	0.0	80.54%	756
BolC2t06532H	BolC2t06532H	Brassica oleracea	99%	0.0	81.79%	729
BolC9t58549H	BolC9t58549H		88%	0.0	60.09%	1267
BraA02t05145Z	BraA02t05145Z	Brassica rapa	96%	0.0	83.15%	733
BraA03t10261Z	BraA03t10261Z		94%	0.0	58.36%	716
BraA10t44106Z	BraA10t44106Z		88%	0.0	61.14%	1296
BraA10t44576Z	BraA10t44576Z		96%	0.0	84.67%	707
BcaB01g05140	BcaB01g05140	Brassica carinata	94%	0.0	56.01%	700
BcaB08g36582	BcaB08g36582		94%	0.0	58.83%	711
BcaC04g18577	BcaC04g18577		88%	0.0	62.02%	715
BcaNung01177	BcaNung01177		99%	0.0	83.09%	737
BcaNung01857	BcaNung01857		99%	0.0	81.52%	729
Phvul.001G151300	Phvul.001G151300.1	Phaseolus vulgaris	88%	0.0	53.50%	704
	Phvul.002G047400.3		89%	0.0	62.22%	707
	Phvul.002G047400.4		0,89	0.0	62.22%	707
Phvul.006G001300	Phvul.006G001300.1		87%	4E-176	43.82%	699
Phvul.007G054500	Phvul.007G054500.1		94%	0.0	60.40%	706
Glyma.01G239200	Glyma.01G239200.1	Glycine max	92%	0.0	60.70%	707
	Glyma.01G239200.3		92%	0.0	60.70%	706
Glyma.03G154000	Glyma.03G154000.1		89%	0.0	53.76%	705
Glyma.10G250100	Glyma.10G250100.2		89%	0.0	61.88%	707
	Glyma.10G250100.4		89%	0.0	61.88%	708
Glyma.11G004300	Glyma.11G004300.1		92%	0.0	61.08%	708
	Glyma.11G004300.3		92%	0.0	61.08%	709
Glyma.19G156400	Glyma.19G156400.1		88%	0.0	54.67%	714
	Glyma.19G156400.2		88%	0.0	55.41%	704
Glyma.20G143500	Glyma.20G143500.1		89%	0.0	61.72%	707
	Glyma.20G143500.2		89%	0.0	60.06%	686
Psat6g208840	Psat6g208840.1	Pisum sativum	94%	0.0	59.80%	704
Psat4g211360	Psat4g211360.1		88%	0.0	61.98%	703
Psat3g064160	Psat3g064160.1		93%	0.0	56.31%	735
Eucgr.H03553	Eucgr.H03553	Eucalyptus grandis	89%	0.0	62.18%	712
Thecc.01G278800	Thecc.01G278800.1	Theobroma cacao	94%	0.0	58.98%	705
Thecc.04G227100	Thecc.04G227100.1		89%	0.0	63.17%	704

Gorai.005G041500	Gorai.005G041500.1	Gossypium raimondii	92%	0.0	61.08%	705
Gorai.006G237100	Gorai.006G237100.1		89%	0.0	61.95%	704
	Gorai.006G237100.2		89%	0.0	59.82%	681
	Gorai.006G237100.3		85%	0.0	63.13%	687
	Gorai.006G237100.4		86%	0.0	67.00%	513
	Gorai.006G237100.5		86%	0.0	62.60%	697
Lsat_1_v5_gn_5_188180	Lsat_1_v5_gn_5_188180.1	Lactuca sativa	95%	0.0	61.16%	757
	Lsat_1_v5_gn_5_188180.2		95%	0.0	61.16%	757
Lsat_1_v5_gn_9_3640	Lsat_1_v5_gn_9_3640.3		94%	0.0	59.86%	726
Lsat_1_v5_gn_3_84821	Lsat_1_v5_gn_3_84821.1		37%	1E-54	53.97%	239
Cpa.g.sc147.32	Cpa.t.sc147.32	Carica papaya	92%	0.0	60.47%	715
Cpa.g.sc370.9	Cpa.t.sc370.9		93%	0.0	61.99%	704
29844.t000205	29844.m003359	Ricinus communis	89%	0.0	63.13%	719
29647.t000089	29647.m002081		84%	0.0	60.09%	598
Ciclev10004440m.g	Ciclev10004440m	Citrus clementina	89%	0.0	62.86%	717
Ciclev10019087m.g	Ciclev10019087m		94%	0.0	61.69%	704
orange1.1g005282m.g	orange1.1g005282m	Citrus sinensis	94%	0.0	61.69%	704
orange1.1g004992m.g	orange1.1g005044m		89%	0.0	62.58%	720
	orange1.1g004992m		89%	0.0	62.58%	720
	orange1.1g005088m		89%	0.0	62.25%	715
	orange1.1g005144m		89%	0.0	62.25%	712
	orange1.1g007101m		75%	0.0	64.80%	618
	orange1.1g007104m		75%	0.0	64.80%	618
	orange1.1g008781m		66%	0.0	63.21%	554
	orange1.1g008940m		86%	0.0	67.67%	548
	orange1.1g010291m		86%	0.0	67.59%	513
	orange1.1g015851m		49%	5E-123	62.78%	399
Misin17G131600	Misin17G131600.1	Miscanthus sinensis	88%	0.0	61.01%	732
	Misin17G131600.2		88%	0.0	60.31%	717
Misin16G132500	Misin16G132500.1		88%	0.0	60.55%	744
EL10Ac7g17234	EL10Ac7g17234.1	Beta vulgaris	94%	0.0	56.13%	744
EL10As3g23353	EL10As3g23353.1		88%	0.0	58.74%	714
Bobra.126_2s0003	Bobra.126_2s0003.1	Botryococcus braunii	94%	0.0	47.89%	707
Cre04.g231516	Cre04.g231516.t1.1	Chlamydomonas reinhardtii	94%	0.0	47.89%	707
	Cre04.g231516.t2.1		84%	4E-179	45.17%	763
UNPLg00364	UNPLg00364.t1	Chromochloris zofingiensis	83%	0.0	47.10%	677
Araha.3287s0012	Araha.3287s0012.1	Arabidopsis halleri	85%	0.0	93.51%	634
Araha.13116s0005	Araha.13116s0005.1		94%	0.0	60.51%	711
	Araha.13116s0005.2		94%	0.0	60.46%	711
Bostr.2902s0040	Bostr.2902s0040.1.p	Boecherastricta	100%	0.0	91.47%	739
Bostr.26527s0273	Bostr.26527s0273.1		94%	0.0	60.63%	710
Bostr.2570s0062	Bostr.2570s0062.1		85%	0.0	60.14%	439
Mamar.0011s0326	Mamar.0011s0326.1	Malcolmia maritima	100%	0.0	89.99%	739
Mamar.0050s1498	Mamar.0050s1498.1		94%	0.0	60.46%	710
Mamar.0016s0220	Mamar.0016s0220.1		94%	0.0	55.71%	698
Alyli.0206s0053	Alyli.0206s0053.1	Alyssum linifolium	96%	0.0	88.89%	707
Alyli.0096s0006	Alyli.0096s0006.1		94%	0.0	59.77%	710
Alyli.0053s0226	Alyli.0053s0226.1		94%	0.0	59.77%	710
	Alyli.0053s0226.2		94%	0.0	59.83%	711
Thlar.0013s1177	Thlar.0013s1177.1	Thlaspi arvense	96%	0.0	85.65%	710
Thlar.0007s0309	Thlar.0007s0309.1		94%	0.0	60.83%	710
AL6G23660	AL6G23660.t1	Arabidopsis lyrata	96%	0.0	92.72%	713
AL6G30820	AL6G30820.t1		94%	0.0	60.80%	710
	AL6G30820.t2		94%	0.0	60.85%	712
AL3G16810	AL3G16810.t1		88%	0.0	57.25%	694
	AL3G16810.t2		88%	0.0	57.25%	694
Aqcoe2G339500	Aqcoe2G339500.1	Aquilegia coerulea	89%	0.0	57.87%	757
AgateH1.02G001300	AgateH1.02G001300.1	Agave tequilana var. Weber's Blue	89%	0.0	58.90%	744
	AgateH1.02G001300.2		89%	0.0	58.90%	744
	AgateH1.02G001300.2		89%	0.0	61.67%	714
	AgateH1.02G001300.3		89%	0.0	58.32%	739
	AgateH1.02G001300.4		89%	0.0	58.03%	737
AgateH1.02G344000	AgateH1.02G344000.1		89%	0.0	60.82%	712
Alyli.0206s0053	Alyli.0206s0053.1	Alyssum linifolium	96%	0.0	88.89%	707
Alyli.0096s0006	Alyli.0096s0006.1		94%	0.0	59.77%	710
Alyli.0053s0226	Alyli.0053s0226.1		94%	0.0	59.77%	710
	Alyli.0053s0226.2		94%	0.0	59.83%	711
evm_27.model.AmTr_v1.0_scaffold00025.384	evm_27.model.AmTr_v1.0_scaffold00025.38	Amborella trichopoda	87%	0.0	59.63%	717
AmTrH2.01G163500	AmTrH2.01G163500.1		87%	0.0	59.63%	717
Anaoc.0004s0117	Anaoc.0004s0117.7	Anacardium occidentale	77%	0.0	70.98%	451
	Anaoc.0004s0117.1		89%	0.0	62.71%	714
	Anaoc.0004s0117.2		86%	0.0	67.79%	513
	Anaoc.0004s0117.3		86%	0.0	67.79%	513
	Anaoc.0004s0117.4		86%	0.0	67.79%	513
	Anaoc.0004s0117.5		77%	0.0	70.98%	452
	Anaoc.0004s0117.6		77%	0.0	70.98%	452
	Anaoc.0004s0117.7		77%	0.0	70.98%	452
	Anaoc.0004s0117.8		89%	0.0	62.71%	714
	Anaoc.0004s0117.9		80%	0.0	64.53%	653
	Anaoc.0004s0117.10		75%	0.0	64.09%	617
	Anaoc.0004s0117.11		89%	0.0	62.71%	714
	Anaoc.0004s0117.12		80%	0.0	64.53%	653
	Anaoc.0004s0117.13		65%	0.0	63.43%	579
Anaoc.0004s0821	Anaoc.0004s0821.1		85%	0.0	65.34%	587
Anaoc.0008s0482	Anaoc.0008s0482.1		89%	0.0	62.97%	715
Aco006295	Aco006295.1	Ananas comosus	90%	0.0	59.31%	706

AndgeH2.09AG127100	AndgeH2.09AG127100.2	Andropogon Gerardi	0%	0	65.00%	0
	AndgeH2.09AG127100.1		71%	0.0	62.62%	615
	AndgeH2.09AG127100.3		0%	0	63.00%	0
AndgeH2.09BG107700	AndgeH2.09BG107700.7		0%	0	64.00%	0
	AndgeH2.09BG107700.6		0%	0	64.00%	0
	AndgeH2.09BG107700.4		0%	0	63.00%	0
	AndgeH2.09BG107700.5		0%	0	64.00%	0
	AndgeH2.09BG107700.2		0%	0	63.00%	0
	AndgeH2.09BG107700.1		87%	0.0	63.94%	593
	AndgeH2.09BG107700.3		0%	0	63.00%	0
AndgeH2.09CG179600	AndgeH2.09CG179600.1		89%	0.0	60.73%	736
arahy.Tifrunner.gnm1.ann1.7RR6EE	arahy.Tifrunner.gnm1.ann1.7RR6EE.1	Arachis hypogaea	62%	0.0	64.48%	487
	arahy.Tifrunner.gnm1.ann1.7RR6EE.2		0%	0	0,64	0
arahy.Tifrunner.gnm1.ann1.C0GFHN	arahy.Tifrunner.gnm1.ann1.C0GFHN.1		88%	0.0	59.02%	685
arahy.Tifrunner.gnm1.ann1.CB6P6Z	arahy.Tifrunner.gnm1.ann1.CB6P6Z.1		94%	0.0	57.38%	724
arahy.Tifrunner.gnm1.ann1.71BXTT	arahy.Tifrunner.gnm1.ann1.71BXTT.1		94%	0.0	56.83%	730
arahy.Tifrunner.gnm1.ann1.825X9M	arahy.Tifrunner.gnm1.ann1.825X9M.1		93%	0.0	56.80%	699
arahy.Tifrunner.gnm1.ann1.GF6ZB7	arahy.Tifrunner.gnm1.ann1.GF6ZB7.1		93%	0.0	56.94%	699
	arahy.Tifrunner.gnm1.ann1.GF6ZB7.2		93%	0.0	56.94%	699
evm.TU.AsparagusV1_07.1797	evm.model.AsparagusV1_07.1797	Asparagus officinalis	94%	0.0	57.79%	714
BPChr05G08852	BPChr05G08852	Betula platyphylla	84%	0.0	64.84%	715
BPChr05G08804	BPChr05G08804		91%	0.0	61.98%	712
BPChr08G05060	BPChr08G05060		84%	0.0	51.90%	656
Bostr.2902s0040	Bostr.2902s0040.1	Boechera stricta	100%	0.0	91.47%	739
Bostr.26527s0273	Bostr.26527s0273.1		94%	0.0	60.63%	710
Bostr.2570s0062	Bostr.2570s0062.1		85%	0.0	60.14%	439
Barbu.8G135600	Barbu.8G135600.1	Brachypodium arbuscula	88%	0.0	60.06%	740
Brahy.S08G0135700	Brahy.S08G0135700.1	Brachypodium hybridum	89%	0.0	59.85%	734
Brahy.D02G0363000	Brahy.D02G0363000.1		88%	0.0	60.21%	787
Brasyl.8G132300	Brasyl.8G132300.1	Brachypodium sylvaticum	88%	0.0	60.06%	739
	Brasyl.8G132300.2		84%	0.0	61.46%	718
	Brasyl.8G132300.3		84%	0.0	61.08%	722
Brast08G128100	Brast08G128100.1	Brachypodium stacei	88%	0.0	60.21%	739
Bmexi.08PG175100	Bmexi.08PG175100.1	Brachypodium mexicanum	84%	0.0	60.95%	773
	Bmexi.08PG175100.2		88%	0.0	60.06%	734
	Bmexi.08PG175100.3		88%	0.0	59.91%	732
	Bmexi.08PG175100.4		88%	0.0	58.99%	725
	Bmexi.08PG175100.5		88%	0.0	58.20%	755
Bmexi.08UG146100	Bmexi.08UG146100.1		88%	0.0	56.87%	759
	Bmexi.08UG146100.2		88%	0.0	59.60%	731
Camar.0268s0010	Camar.0268s0010.1	Cakile maritima	95%	0.0	84.41%	741
Camar.0675s0029	Camar.0675s0029.1		96%	0.0	85.21%	710
Camar.0558s0016	Camar.0558s0016.1		94%	0.0	60.00%	712
Cagra.1535s0001	Cagra.1535s0001.1	Capsella grandiflora	95%	0.0	83.45%	716
Cagra.4243s0018	Cagra.4243s0018.1		94%	0.0	60.83%	712
Cagra.4243s0018	Cagra.4243s0018.2		94%	0.0	60.77%	711
Caril.07G091200	Caril.07G091200.1	Carya illinoinensis	89%	0.0	62.48%	717
Caril.01G159600	Caril.01G159600.1		83%	0.0	64.72%	760
	Caril.01G159600.2		83%	0.0	64.72%	760
	Caril.01G159600.3		94%	0.0	60.60%	705
	Caril.01G159600.4		94%	0.0	60.60%	705
	Caril.01G159600.5		91%	0.0	61.37%	683
Caril.02G095600	Caril.02G095600.1		87%	0.0	60.80%	730
	Caril.02G095600.2		87%	0.0	63.33%	704
	Caril.02G095600.3		87%	0.0	63.33%	704
	Caril.02G095600.4		87%	0.0	63.33%	704
	Caril.02G095600.5		87%	0.0	63.33%	704
	Caril.02G095600.6		87%	0.0	61.02%	681
	Caril.02G095600.7		87%	0.0	62.94%	708
Caden.10G092800	Caden.10G092800.1	Castanea dentata	93%	0.0	62.03%	708
Caden.02G024900	Caden.02G024900.1		88%	0.0	63.68%	735
CmMahoganyH1.10G076000	CmMahoganyH1.10G076000.1	Castanea mollissima Mahogany	93%	0.0	62.03%	708
	CmMahoganyH1.10G076000.2		93%	0.0	61.60%	703
CmMahoganyH1.02G020700	CmMahoganyH1.02G020700.1		88%	0.0	63.68%	735
Caamp.1041s1256	Caamp.1041s1256.1	Caulanthus amplexicaulis	99%	0.0	86.18%	736
Caamp.1037s0435	Caamp.1037s0435.1		94%	0.0	59.83%	711
	Caamp.1037s0435.2		94%	0.0	59.77%	711
Caamp.0105s0848	Caamp.0105s0848.1		94%	0.0	59.69%	711
	Caamp.0105s0848.2		94%	0.0	59.63%	711
CepurGG1.3G150100	CepurGG1.3G150100.1	Ceratodon purpureus GG1	95%	0.0	54.41%	722
	CepurGG1.3G150100.2		95%	0.0	56.03%	702
	CepurGG1.3G150100.3		91%	0.0	56.87%	680
	CepurGG1.3G150100.4		95%	0.0	55.79%	705
	CepurGG1.3G150100.5		95%	0.0	54.63%	720
	CepurGG1.3G150100.6		81%	0.0	57.62%	596
	CepurGG1.3G150100.7		81%	0.0	54.21%	634
	CepurGG1.3G150100.8		81%	0.0	57.90%	593
	CepurGG1.3G150100.9		78%	0.0	55.95%	590
	CepurGG1.3G150100.10		75%	0.0	53.01%	584
Ceric.36G016000	Ceric.36G016000.1	Ceratopteris richardii	92%	0.0	58.33%	711
	Ceric.36G016000.2		92%	0.0	58.42%	711
Cecan.1G431700	Cecan.1G431700.1	Cercis canadenses	95%	0.0	61.69%	742
	Cecan.1G431700.2		93%	0.0	62.50%	711
Cecan.2G164300	Cecan.2G164300.1		94%	0.0	61.01%	706
	Cecan.2G164300.2		94%	0.0	61.01%	707
	Cecan.2G164300.3		94%	0.0	61.01%	707
	Cecan.2G164300.4		94%	0.0	61.01%	707
	Cecan.2G164300.5		94%	0.0	60.43%	706

Chala.07G138200	Chala.07G138200.1	Chasmanthium laxum	88%	0.0	60.88%	750
Ca_04616	Ca_04616	Cicer arietinum	89%	0.0	61.52%	705
Ca_07379	Ca_07379		92%	0.0	57.72%	732
CKAN_00522800	CKAN_00522800	Cinnamomum kanehirae	94%	0.0	59.68%	703
CKAN_02407500	CKAN_02407500		87%	0.0	63.51%	704
Clevi.0008s1094	Clevi.0008s1094.1	Cleome violacea	96%	0.0	69.15%	696
Clevi.0003s1966	Clevi.0003s1966.1		94%	0.0	60.11%	711
	Clevi.0003s1966.2		94%	0.0	60.11%	711
CamerWinkler.02G102500	CamerWinkler.02G102500.1	Corylus americana	95%	0.0	60.73%	710
CamerWinkler.01G086200	CamerWinkler.01G086200.1		88%	0.0	63.72%	704
	CamerWinkler.01G086200.2		88%	0.0	61.43%	682
	CamerWinkler.01G086200.3		88%	0.0	61.43%	682
	CamerWinkler.01G086200.4		88%	0.0	63.72%	705
Crahi.0006s0149	Crahi.0006s0149.1	Crambe hispanica	96%	0.0	85.69%	729
Crahi.0002s0039	Crahi.0002s0039.1		99%	0.0	84.22%	728
Crahi.1073s0019	Crahi.1073s0019.1		94%	0.0	59.94%	710
Crahi.0488s0065	Crahi.0488s0065.1		92%	0.0	58.10%	708
Cucsa.254160	Cucsa.254160.1	Cucumis sativus	88%	0.0	62.80%	703
	Cucsa.254160.2		86%	0.0	67.13%	530
DCAR_002731	DCAR_002731	Daucus carota	95%	0.0	58.11%	703
DCAR_018420	DCAR_018420		95%	0.0	58.17%	696
Desop.0240s1381	Desop.0240s1381.1	Descurainia sophioides	96%	0.0	89.17%	707
Desop.0240s0737	Desop.0240s0737.1		94%	0.0	59.77%	710
Desop.0015s0242	Desop.0015s0242.1		91%	0.0	54.22%	634
Dioal.07G052900	Dioal.07G052900.1	Dioscorea alata	88%	0.0	62.02%	718
	Dioal.07G052900.2		88%	0.0	62.02%	718
Dicom.01G166000	Dicom.01G166000.1	Diphasiastrum complanatum	98%	0.0	54.68%	708
	Dicom.01G166000.2		93%	0.0	56.52%	705
	Dicom.01G166000.3		93%	0.0	56.52%	705
	Dicom.01G166000.5		93%	0.0	56.52%	705
	Dicom.01G166000.4		93%	0.0	56.52%	705
	Dicom.01G166000.6		93%	0.0	56.52%	705
	Dicom.01G166000.7		93%	0.0	56.44%	706
	Dicom.01G166000.8		93%	0.0	56.44%	706
Eruve.0575s0004	Eruve.0575s0004.1	Eruca vesicaria	96%	0.0	85.79%	707
Eruve.0337s0070	Eruve.0337s0070.1		96%	0.0	85.65%	707
Eruve.2518s0012	Eruve.2518s0012.1		99%	0.0	81.72%	749
Eruve.2532s0001	Eruve.2532s0001.1		96%	0.0	83.47%	716
Eruve.3085s0001	Eruve.3085s0001.1		88%	0.0	62.23%	717
Eruve.0105s0014	Eruve.0105s0014.1		88%	0.0	62.23%	717
Distr.0002s126700	Distr.0002s126700.1	Diptychocarpus strictus	95%	0.0	85.51%	703
Distr.0002s194500	Distr.0002s194500.1		94%	0.0	60.68%	711
	Distr.0002s194500.2		94%	0.0	60.63%	711
ELECO.r07.5BG0422180	ELECO.r07.5BG0422180.1	Eleusine coracana	85%	0.0	61.89%	727
ELECO.r07.5AG0374120	ELECO.r07.5AG0374120.1		85%	0.0	61.57%	727
Eusyr.0026s0371	Eusyr.0026s0371.1	Euclidium syriacum	96%	0.0	83.83%	711
Eusyr.0132s0171	Eusyr.0132s0171.1		94%	0.0	60.06%	710
Eusyr.0023s0214	Eusyr.0023s0214.1		88%	0.0	57.32%	697
Thhalv10012773m.g	Thhalv10012773m	Eutrema salsugineum	99%	0.0	84.01%	742
Thhalv10012813m.g	Thhalv10012813m		94%	0.0	59.69%	710
FvH4_4g15040	FvH4_4g15040.t1	Fragaria vesca	85%	0.0	61.99%	639
	FvH4_4g15040.t2		85%	0.0	61.99%	770
	FvH4_4g15040.t3		86%	0.0	66.19%	575
	FvH4_4g15040.t4		94%	0.0	60.60%	704
	FvH4_4g15040.t5		80%	0.0	61.62%	705
	FvH4_4g15040.t6		94%	0.0	59.43%	711
	FvH4_4g15040.t7		91%	0.0	61.52%	719
FvH4_2g23530	FvH4_2g23530.t1		90%	0.0	61.50%	708
	FvH4_2g23530.t2		60%	1,0E-178	62.47%	513
	FvH4_2g23530.t3		86%	0.0	62.07%	674
	FvH4_2g23530.t4		80%	0.0	62.92%	707
	FvH4_2g23530.t5		75%	0.0	62.66%	652
	FvH4_2g23530.t6		42%	2,0E-113	61.02%	373
HanXRQChr11g0336141	HanXRQChr11g0336141	Helianthus annuus	95%	0.0	59.46%	704
HanXRQChr01g0003111	HanXRQChr01g0003111		95%	0.0	61.31%	707
Hyque.04G178000	Hyque.04G178000.1	Hydrangea quercifolia	95%	0.0	60.85%	707
Hyque.10G175000	Hyque.10G175000.1		94%	0.0	61.00%	705
Ibeam.3272s0005	Ibeam.3272s0005.1	Iberis amara	94%	0.0	59.60%	711
	Ibeam.3272s0005.2		94%	0.0	59.55%	710
Ibeam.2342s0017	Ibeam.2342s0017.1		94%	0.0	59.55%	710
Ibeam.1976s0005	Ibeam.1976s0005.1		95%	0.0	85.15%	711
Isati.6497s0005	Isati.6497s0005.1	Isatis tinctoria	96%	0.0	86.06%	707
Isati.7402s0006	Isati.7402s0006.1		87%	0.0	90.24%	543
Isati.0539s0031	Isati.0539s0031.1		96%	0.0	85.92%	707
Isati.7745s0002	Isati.7745s0002.1		96%	0.0	85.77%	707
Joasc.03G088600	Joasc.03G088600.1	Joinvillea ascendens	88%	0.0	61.89%	733
Kaladp0013s0013	Kaladp0013s0013.1	Kalanchoe fedtschenkoi	94%	0.0	57.59%	707
Lcu.2RBY.6g052660	Lcu.2RBY.6g052660.1	Lens culinaris	89%	0.0	55.62%	686
Lcu.2RBY.1g037100	Lcu.2RBY.1g037100.1		94%	0.0	60.09%	704
Lcu.2RBY.5g000760	Lcu.2RBY.5g000760.1		88%	0.0	57.86%	760
Lesat.0028s0199	Lesat.0028s0199.1	Lepidium sativum	94%	0.0	60.34%	712
	Lesat.0028s0199.2		94%	0.0	60.28%	711
Lesat.0059s1052	Lesat.0059s1052.1		94%	0.0	60.00%	711
Lesat.0095s0314	Lesat.0095s0314.1		96%	0.0	87.18%	710
Liphi.01G183500	Liphi.01G183500.1	Lindenbergia philippensis	95%	0.0	59.44%	718
	Liphi.01G183500.2		95%	0.0	58.87%	713
Liphi.11G032600	Liphi.11G032600.1		93%	0.0	59.68%	710
	Liphi.11G032600.2		93%	0.0	59.77%	709
Lus10010527.g	Lus10010527	Linum usitatissimum	87%	0.0	63.93%	717
Lus10022039.g	Lus10022039		94%	0.0	52.57%	723
Lus10034070.g	Lus10034070		87%	0.0	62.95%	884
Lus10042583.g	Lus10042583		94%	0.0	53.49%	709
Lj1g0006129	Lj1g0006129.3	Lotus japonicus	87%	0.0	59.61%	726
Lj1g0011717	Lj1g0011717.1		78%	0.0	50.77%	680
Lj1g0011876	Lj1g0011876.1		93%	0.0	54.94%	699
Lj1g0018523	Lj1g0018523.1		86%	0.0	52.17%	732
Lj3g0008315	Lj3g0008315.1		87%	0.0	48.93%	613
Lj5g0014423	Lj5g0014423.1		89%	0.0	62.03%	706

Lalb_Chr01g0020141	Lalb_Chr01g0020141	Lupinus albus	93%	0.0	54.83%	690
Lalb_Chr05g0210891	Lalb_Chr05g0210891		88%	0.0	61.13%	694
Lalb_Chr16g0376391	Lalb_Chr16g0376391		89%	0.0	60.55%	687
Mamar.0011s0326	Mamar.0011s0326.1	Malcolmia maritima	100%	0.0	89.99%	739
Mamar.0050s1498	Mamar.0050s1498.1		94%	0.0	60.46%	710
Mamar.0016s0220	Mamar.0016s0220.1		94%	0.0	55.71%	698
Mapoly0013s0109	Mapoly0013s0109.1	Marchantia polymorpha	92%	0.0	54.47%	751
Medtr1g104870	Medtr1g104870.1	Medicago truncatula	94%	0.0	59.94%	704
Medtr5g005940	Medtr5g005940.1		78%	0.0	61.45%	601
	Medtr5g005940.2		78%	0.0	61.45%	696
Medtr7g094900	Medtr7g094900.1		83%	0.0	61.26%	716
Medtr7g094910	Medtr7g094910.1		84%	0.0	60.61%	697
	Medtr7g094910.2		75%	0.0	62.46%	683
MgTOL.I00297	MgTOL.I00297.1	Mimulus guttatus	91%	0.0	59.12%	698
MgTOL.N2070	MgTOL.N2070.1		94%	0.0	60.40%	715
Myper.0004s1359	Myper.0004s1359.1	Myagrum perfoliatum	99%	0.0	84.08%	736
Myper.0004s1981	Myper.0004s1981.1		94%	0.0	60.20%	710
Nycol.I00850	Nycol.I00850.1	Nymphaea colorata	93%	0.0	56.73%	698
Oeu041702	Oeu041702.3	Olea europaea	89%	0.0	63.69%	716
Oeu028070	Oeu028070.1		89%	0.0	60.58%	751
Oropetium_20150105_05154	Oropetium_20150105_05154A	Oropetium thomaeum	88%	3E-116	70.45%	587
PhHAL3G279400	PhHAL3G279400.1	Panicum hallii	88%	0.0	60.15%	742
Podel.08G175100	Podel.08G175100.1	Populus deltoides	87%	0.0	63.46%	718
Podel.10G086700	Podel.10G086700.1		87%	0.0	63.00%	717
Podel.05G221700	Podel.05G221700.1		89%	0.0	62.33%	698
Podel.02G058100	Podel.02G058100.1		88%	0.0	61.37%	704
Ptrif.0009s0499	Ptrif.0009s0499.1	Poncirus trifoliata	89%	0.0	62.71%	717
Ptrif.0003s3479	Ptrif.0003s3479.1		94%	0.0	61.69%	704
Pp3c7_11520	Pp3c7_11520V3.1	Physcomitrium patens	69%	0.0	61.13%	551
	Pp3c7_11520V3.2		86%	0.0	60.78%	674
	Pp3c7_11520V3.3		93%	0.0	56.44%	709
	Pp3c7_11520V3.4		93%	0.0	56.69%	706
	Pp3c7_11520V3.5		93%	0.0	56.69%	706
Pp3c11_17010	Pp3c11_17010V3.1		90%	0.0	57.31%	709
	Pp3c11_17010V3.2		90%	0.0	55.73%	728
	Pp3c11_17010V3.3		90%	0.0	55.49%	731
	Pp3c11_17010V3.4		90%	0.0	57.31%	709
	Pp3c11_17010V3.5		90%	0.0	57.57%	706
Phacu.WLD.002G006700	Phacu.WLD.002G006700.1	Phaseolus acutifolius	89%	0.0	62.22%	707
	Phacu.WLD.002G006700.1		89%	0.0	62.22%	707
	Phacu.WLD.002G006700.2		86%	0.0	63.49%	694
Phacu.WLD.007G055300	Phacu.WLD.007G055300.1		94%	0.0	60.11%	707
	Phacu.WLD.007G055300.2		90%	0.0	61.07%	714
Phacu.WLD.001G179100	Phacu.WLD.001G179100.1		88%	0.0	52.75%	706
	Phacu.WLD.001G179100.2		79%	0.0	54.30%	577
Phlat.07G120000	Phlat.07G120000.1	Pharus latifolius	88%	0.0	60.82%	731
Pavag09G136000	Pavag09G136000.1	Paspalum vaginatum	88%	0.0	61.31%	741
Pavir.3KG262426	Pavir.3KG262426.1	Panicum virgatum	84%	0.0	60.93%	741
	Pavir.3KG262426.2		84%	0.0	60.93%	739
	Pavir.3KG262426.3		75%	0.0	63.65%	685
	Pavir.3KG262426.3		75%	0.0	63.65%	716
	Pavir.3KG262426.4		71%	0.0	62.62%	662
Pavir.3NG076953	Pavir.3NG076953.1		85%	0.0	61.60%	741
	Pavir.3NG076953.2		85%	0.0	61.60%	739
	Pavir.3NG076953.3		85%	0.0	60.34%	726
	Pavir.3NG076953.4		83%	0.0	62.08%	722
Poman.08G130300	Poman.08G130300.1	Populus nigra x maximowiczii	87%	0.0	63.76%	717
Poman.05G171800	Poman.05G171800.1		89%	0.0	62.63%	708
	Poman.05G171800.2		86%	0.0	63.58%	680
Poman.10G071900	Poman.10G071900.1		87%	0.0	63.00%	717
Poman.02G047100	Poman.02G047100.1		88%	0.0	61.22%	704
	Poman.02G047100.2		71%	0.0	62.48%	648
	Poman.02G047100.3		71%	0.0	62.48%	648
	Poman.02G047100.4		79%	0.0	63.61%	736
PtXaAlbH.05G161100	PtXaAlbH.05G161100.1		89%	0.0	62.31%	696
PtXaAlbH.10G070200	PtXaAlbH.10G070200.1		87%	0.0	61.78%	725
	PtXaAlbH.10G070200.2	Populus tremula x Populus alba	87%	0.0	62.54%	717
	PtXaAlbH.10G070200.3		87%	0.0	62.54%	716
	PtXaAlbH.10G070200.4		87%	0.0	61.62%	709
	PtXaAlbH.10G070200.5		87%	0.0	62.54%	715
	PtXaAlbH.10G070200.6		87%	0.0	62.23%	713
PtXaAlbH.02G045300	PtXaAlbH.02G045300.1		88%	0.0	61.19%	706
	PtXaAlbH.02G045300.2		88%	0.0	61.37%	796
PtXaAlbH.08G127900	PtXaAlbH.08G127900.1		94%	0.0	60.08%	717
	PtXaAlbH.08G127900.1		94%	0.0	60.08%	717
	PtXaAlbH.08G127900.2		94%	0.0	60.08%	717
	PtXaAlbH.08G127900.3		94%	0.0	60.08%	717
	PtXaAlbH.08G127900.4		94%	0.0	60.08%	717
	PtXaAlbH.08G127900.5		94%	0.0	60.08%	717
Potri.008G150100	Potri.008G150100.1	Populus trichocarpa	87%	0.0	63.76%	717
	Potri.008G150100.2		84%	0.0	64.71%	704
	Potri.008G150100.1		87%	0.0	63.76%	717
	Potri.008G150100.2		84%	0.0	64.71%	689
	Potri.008G150100.3		79%	0.0	65.58%	665
	Potri.008G150100.4		86%	0.0	68.38%	529
	Potri.008G150100.5		79%	0.0	64.98%	661
	Potri.008G150100.6		84%	0.0	64.56%	713
	Potri.008G150100.6		84%	0.0	64.56%	704
Potri.005G210000	Potri.005G210000.1		89%	0.0	62.33%	698
Potri.002G052700	Potri.002G052700.2		88%	0.0	61.37%	704
Potri.010G090900	Potri.010G090900.1		87%	0.0	62.10%	713
	Potri.010G090900.2		87%	0.0	62.84%	713
	Potri.010G090900.3		87%	0.0	61.31%	781
	Potri.010G090900.4		72%	0.0	62.94%	648
	Potri.010G090900.7		77%	0.0	62.71%	709
	Potri.010G090900.8		72%	0.0	62.02%	642
	Potri.010G090900.9		77%	0.0	63.59%	716
	Potri.010G090900.10		72%	0.0	62.94%	648
	Potri.010G090900.11		72%	0.0	62.94%	648
	Potri.010G090900.12		74%	0.0	64.13%	694
	Potri.010G090900.13		67%	0.0	63.55%	609
	Potri.010G090900.14		70%	0.0	67.76%	590
FUN_042764	FUN_042764-T1	Portulaca amilis	87%	0.0	61.55%	723
FUN_014427	FUN_014427-T1		94%	0.0	55.92%	690
	FUN_014427-T2		77%	0.0	55.65%	601
Prupe.1G155300	Prupe.1G155300.1	Prunus persica	94%	0.0	59.71%	704
	Prupe.1G155300.2		94%	0.0	59.71%	704
Prupe.8G240700	Prupe.8G240700.1		95%	0.0	60.25%	710
Qurub.06G239500	Qurub.06G239500.1	Quercus rubra	89%	0.0	63.73%	732
Qurub.12G088800	Qurub.12G088800.1		93%	0.0	61.75%	708
Roisl.0210s0994	Roisl.0210s0994.1	Rorippa islandica	99%	0.0	84.98%	739
Roisl.0210s1650	Roisl.0210s1650.1		94%	0.0	60.62%	711
	Roisl.0210s1650.2		94%	0.0	60.57%	711
Roisl.0039s0140	Roisl.0039s0140.1		87%	0.0	57.52%	701
Sapur.005G165700	Sapur.005G165700.1	Salix purpurea	88%	0.0	62.33%	701
	Sapur.005G165700.2		71%	0.0	63.81%	658
Sapur.010G058700	Sapur.010G058700.1		87%	0.0	62.08%	713
	Sapur.010G058700.2		86%	0.0	65.14%	712
	Sapur.010G058700.3		61%	0.0	63.39%	605
Sapur.002G041300	Sapur.002G041300.1		88%	0.0	61.68%	704
	Sapur.002G041300.2		79%	0.0	64.29%	701
Sapur.008G121400	Sapur.008G121400.1		89%	0.0	62.29%	717
	Sapur.008G121400.2		86%	0.0	67.12%	711
	Sapur.008G121400.3		85%	0.0	63.51%	701

Sp6g30920	Sp6g30920.1	Schrenkiella parvula	95%	0.0	84.95%	711
Sp6g25080	Sp6g25080.1		94%	0.0	58.92%	710
164824	164824	Selaginella moellendorffii	87%	0.0	58.09%	701
Sialb.0278s0087	Sialb.0278s0087.1	Sinapis alba	95%	0.0	84.79%	710
Sialb.0009s11174	Sialb.0009s11174.1		95%	0.0	84.93%	710
Sialb.0193s0098	Sialb.0193s0098.1		94%	0.0	59.69%	711
Sialb.0056s0376	Sialb.0056s0376.1		94%	0.0	59.74%	712
Sialb.0056s0376	Sialb.0056s0376.2		94%	0.0	59.74%	712
Sphfalx01G181900	Sphfalx01G181900.1	Sphagnum fallax	89%	0.0	57.51%	714
	Sphfalx01G181900.2		89%	0.0	57.77%	711
Sphfalx02G196900	Sphfalx02G196900.1		94%	0.0	55.87%	713
	Sphfalx02G196900.2		94%	0.0	56.12%	710
Sphmag02G198100	Sphmag02G198100.1	Sphagnum magellanicum	89%	0.0	57.88%	713
	Sphmag02G198100.2		89%	0.0	58.14%	710
Sphmag01G172800	Sphmag01G172800.1		89%	0.0	57.51%	714
	Sphmag01G172800.2		89%	0.0	57.77%	711
Spov3_chr4.02900	Spov3_chr4.02900	Spinacia oleracea	94%	0.0	58.40%	709
Spov3_chr3.03740	Spov3_chr3.03740		88%	0.0	56.75%	659
Spipo22G0007800	Spipo22G0007800	Spirodela polyrhiza	83%	0.0	63.41%	680
Thint.S01G292400	Thint.S01G292400.1	Thinopyrum intermedium	89%	0.0	60.30%	740
Thint.J01G305100	Thint.J01G305100.1		89%	0.0	60.30%	742
Thint.V01G294800	Thint.V01G294800.1		88%	0.0	60.52%	743
Thupl.29378015s0033	Thupl.29378015s0033.1	Thuja plicata	90%	0.0	57.74%	819
Tp57577_TGAC_v2_gene9920	Tp57577_TGAC_v2_mRNA10257	Trifolium pratense	80%	0.0	62.12%	578
Tp57577_TGAC_v2_gene37872	Tp57577_TGAC_v2_mRNA39151		88%	0.0	62.16%	737
Tp57577_TGAC_v2_gene5509	Tp57577_TGAC_v2_mRNA5699		92%	0.0	60.35%	704
Urofu.3G293300	Urofu.3G293300.1	Urochloa fusca	88%	0.0	60.46%	746
	Urofu.3G293300.2		88%	0.0	60.46%	744
	Urofu.3G293300.3		79%	0.0	62.84%	683
	Urofu.3G293300.4		87%	0.0	64.68%	517
	Urofu.3G293300.5		87%	0.0	64.68%	517
	Urofu.3G293300.6		61%	0.0	63.50%	465
Vadar_g31390	Vadar_g31390.t1	Vaccinium darrowii	94%	0.0	61.80%	706
Vadar_g42337	Vadar_g42337.t1		94%	0.0	60.66%	703
Vigun02g197000	Vigun02g197000.1	Vigna unguiculata	89%	0.0	62.31%	778
	Vigun02g197000.3		89%	0.0	62.31%	778
	Vigun02g197000.4		89%	0.0	62.31%	778
	Vigun02g197000.5		89%	0.0	62.31%	778
Vigun07g242500	Vigun07g242500.1		94%	0.0	60.00%	706
	Vigun07g242500.2		94%	0.0	60.00%	706
	Vigun07g242500.3		94%	0.0	60.00%	706
Zosma02g23080	Zosma02g23080.1	Zostera marina	84%	0.0	57.91%	698
Zosma01g37860	Zosma01g37860.1		85%	0.0	60.34%	701
Nitab4.5_0000458g0070	Nitab4.5_0000458g0070	Nicotiana tabacum	95%	0.0	57.28%	729
Nitab4.5_0000759g0190	Nitab4.5_0000759g0190		89%	0.0	59.64%	680
Nitab4.5_0006749g0030	Nitab4.5_0006749g0030		80%	0.0	58.71%	617
Nitab4.5_0008797g0060	Nitab4.5_0008797g0060		89%	0.0	57.38%	660
NbS00014381g0003	NbS00014381g0003.1	Nicotiana benthamiana	91%	0.0	60.09%	691
NbS00024271g0016	NbS00024271g0016.1		91%	0.0	60.56%	677
NbS00032920g0003	NbS00032920g0003.1		85%	0.0	60.88%	651
NbS00022240g0022	NbS00022240g0022.1		86%	0.0	57.50%	697

PtXaAlbH.03G148200	PtXaAlbH.03G148200.1	*Populus tremula x Populus alba*	95%	6E-89	68.78%	220
	PtXaAlbH.03G148200.2		90%	1E-77	65.28%	207
	PtXaAlbH.03G148200.3		63%	5E-72	80.74%	179
PtXaAlbH.01G035600	PtXaAlbH.01G035600.1		95%	6E-85	65.85%	221
Potri.003G184925	Potri.003G184925.1	*Populus trichocarpa*	95%	8E-89	68.78%	220
	Potri.003G184925.2		63%	2E-72	80.74%	152
Potri.001G041900	Potri.001G041900.1		95%	5E-87	67.80%	221
	Potri.001G041900.5		64%	7E-73	78.99%	152
FUN_040438	FUN_040438-T1	*Portulaca amilis*	93%	2E-64	57.28%	211
Prupe.3G103600	Prupe.3G103600.1	*Prunus persica*	93%	2E-86	67.98%	210
	Prupe.3G103600.2		66%	9E-74	77.93%	147
	Prupe.3G103600.3		66%	9E-74	77.93%	147
Qurub.05G179800	Qurub.05G179800.1	*Quercus rubra*	96%	4E-86	62.98%	220
	Qurub.05G179800.2		81%	1E-74	65.90%	177
	Qurub.05G179800.3		81%	1E-74	65.90%	177
30131.t000042	30131.m006891	*Ricinus communis*	100%	1E-92	62.33%	230
Roisl.0115s0146	Roisl.0115s0146.1	*Rorippa islandica*	95%	1E-126	89.37%	216
Sspon.04G0001710-2C	Sspon.04G0001710-2C	*Saccharum spontaneum*	89%	3E-66	59.61%	319
Sspon.04G0001710-3D	Sspon.04G0001710-3D		89%	6E-66	59.11%	313
Sapur.003G140400	Sapur.003G140400.1	*Salix purpurea*	95%	2E-85	66.34%	220
Sapur.001G032000	Sapur.001G032000.1		95%	4E-82	62.93%	219
Sp5g34800	Sp5g34800.1	*Schrenkiella parvula*	100%	3E-130	90.14%	209
GWHGASIY033783	GWHGASIY033783	*Secale cereale*	86%	1E-65	57.62%	220
GWHGASIY033046	GWHGASIY033046		86%	2E-65	57.14%	220
5289	5289	*Selaginella moellendorffii*	62%	4E-61	65.67%	136
Sevir.1G355800	Sevir.1G355800.1	*Setaria viridis*	89%	8E-68	58.82%	207
	Sevir.1G355800.2		89%	8E-68	58.82%	207
Sialb.0005s0947	Sialb.0005s0947.1	*Sinapis alba*	100%	2E-120	84.23%	221
	Sialb.0005s0947.2		100%	2E-121	84.16%	220
Sialb.0005s0646	Sialb.0005s0646.1		96%	2E-119	85.71%	213
	Sialb.0005s0646.2		96%	1E-120	86.12%	212
Solyc03g117670	Solyc03g117670.4	*Solanum lycopersicum*	88%	2E-83	64.06%	241
PGSC0003DMG400014155	PGSC0003DMT400036704	*Solanum tuberosum*	73%	1,00E-73	66.67%	270
	PGSC0003DMT400036701		47%	8,00E-43	66.67%	225
	PGSC0003DMT400036702		73%	7,00E-74	66.67%	264
	PGSC0003DMT400036703		47%	1,00E-42	66.67%	223
Sobic.004G328000	Sobic.004G328000.3	*Sorghum bicolor*	89%	1E-67	59.90%	207
Sphfalx03G101000	Sphfalx03G101000.1	*Sphagnum fallax*	63%	5E-60	67.41%	322
Sphfalx18G098200	Sphfalx18G098200.1		61%	1E-55	66.15%	344
	Sphfalx18G098200.2		61%	2,00E-57	66.15%	316
	Sphfalx18G098200.3		33%	1,00E-30	68.06%	253
	Sphfalx18G098200.4		33%	1,00E-30	68.06%	282
Sphmag03G095600	Sphmag03G095600.1	*Sphagnum magellanicum*	63%	6E-60	67.41%	322
Sphmag18G097300	Sphmag18G097300.1		62%	2E-55	66.92%	344
Spov3_chr5.02035	Spov3_chr5.02035	*Spinacia oleracea*	86%	1E-74	61.38%	208
Spipo28G0013100	Spipo28G0013100	*Spirodela polyrhiza*	90%	8,00E-68	53.59%	244
Thecc.06G046600	Thecc.06G046600.1	*Theobroma cacao*	97%	9E-91	68.75%	209
	Thecc.06G046600.2		75%	8,00E-78	73.91%	163
	Thecc.06G046600.3		70%	1,00E-75	76.67%	181
Thint.S06G379700	Thint.S06G379700.1	*Thinopyrum intermedium*	88%	7E-67	56.28%	220
	Thint.S06G379700.2		88%	7E-67	56.28%	220
	Thint.S06G379700.3		71%	2E-67	69.87%	166
Thint.J06G419300	Thint.J06G419300.1		71%	8E-66	69.23%	219
	Thint.J06G419300.2		71%	2E-66	69.23%	165
Thlar.0008s0131	Thlar.0008s0131.1	*Thlaspi arvense*	95%	7E-132	90.87%	217
Thupl.29377465s0003	Thupl.29377465s0003.1	*Thuja plicata*	61%	5E-60	67.69%	191
	Thupl.29377465s0003.2		61%	5E-60	67.69%	191
	Thupl.29377465s0003.4		61%	5E-60	67.69%	192
	Thupl.29377465s0003.5		61%	5E-60	67.69%	191
	Thupl.29377465s0003.6		61%	5E-60	67.69%	191
	Thupl.29377465s0003.8		61%	5E-60	67.69%	192
	Thupl.29377465s0003.11		61%	5E-60	67.69%	192
Tp57577_TGAC_v2_gene3367	Tp57577_TGAC_v2_mRNA3464	*Trifolium pratense*	94%	3E-90	63.59%	210
TraesCS6D03G0782500	TraesCS6D03G0782500.1	*Triticum aestivum*	71%	1E-65	69.23%	219
TraesCS6A03G0911900	TraesCS6A03G0911900.1		71%	1E-65	69.87%	292
TraesCS6B03G1096200	TraesCS6B03G1096200.1		71%	2E-65	69.23%	219
Urofu.1G327800	Urofu.1G327800.1	*Urochloa fusca*	71%	1E-66	70.06%	211
	Urofu.1G327800.2		89%	2E-70	60.40%	207
	Urofu.1G327800.3		59%	2E-39	57.04%	183
	Urofu.1G327800.4		89%	2E-70	60.40%	207
	Urofu.1G327800.5		41%	1E-36	72.22%	187
Vadar_g14679	Vadar_g14679.t1	*Vaccinium darrowii*	94%	3E-86	58.41%	228
Vigun08g128600	Vigun08g128600.1	*Vigna unguiculata*	95%	2E-84	61.26%	225
	Vigun08g128600.2		95%	7E-86	63.51%	214
GSVIVG01016103001	GSVIVG01016103001	*Vitis vinifera*	91%	4E-81	63.45%	201
Zm00001d018364	Zm00001d018364_T001	*Zea mays B73*	89%	4E-67	58.25%	211
Zm00007a00050657	Zm00007a00050657	*Zea mays B104*	89%	1E-65	57.21%	291
Zosma03g12720	Zosma03g12720.1	*Zostera marina*	59%	5E-55	67.72%	585

Table S4. Attributions for the main vibrational bands identified in the bio-fingerprint region.

Band (cm^{-1})	Stretch vibrations	Assignment		Reference
**1744**	C=O	Lipids		Durak & Depciuch, 2020
**1636**	C=O	Protein (Amide I)		Tessaro et al., 2022
**1535**	N-H	Lipids and protein (Amide II)		Durak & Depciuch, 2020
**1439**	C-H	Lipids and protein		Durak & Depciuch, 2020
**1242**	C-O	Protein		Durak & Depciuch, 2020
**1230-1235**	O-H	Carbohydrate	Lignin	Dinant et al., 2019
**1090 - 1100**	C-O and C-C		Pectin	Liu et al., 2021
**1160**	C-H		Lignin	Dinant et al., 2019
**1065**	C-O		Sucrose	Hashimoto et al., 2005
**1055**	C-OH		Sucrose	Hashimoto et al 2005
**1052**	C-H		Crystalline Cellulose	Liu et al., 2021
**1024**	C-O		Cellulose	López-Malvar et al., 2021
**1064**	C=C		Hemicellulose	Liu et al., 2021
**991**	α,α-1,1 linkage		trehalose	San-Blas et al., 2011
**972**	O-CH_3		Pectin	Schulz & Baranska, 2007
**954**	C-O-C		Amylose	Liu et al., 2004

Table S5. Percentage differences in physiological parameters of KO and KD lines relative to Col-0.

**Measured variable**	**Figure**	**Mean WT (Col-0)**	**Mean *aip10-1* (KO)**	**Mean *aip10-2* (KD)**	**Dif. KO vs Col (%)**	**Dif. KD vs Col (%)**	**Interpretation**
Rosette area	Fig. 3d	5,33	8,1	6,88	52%	29%	KO > KD
Number of leaves	Fig. 3e	12,5	14,1	13,9	12,80%	11,20%	KO > KD
Fresh weight	Fig. 3g	0,08	0,13	0,11	62,50%	37,50%	KO > KD
Dry weight	Fig. 3h	0,012	0,04	0,02	233%	66,67%	KO > KD
Main axis of inflorescence	Fig. 3j	39,4	46,6	44,4	18,30%	12,70%	KO > KD
Number of branches from the main axis	Fig. 3l	3,06	4,53	3,28	48%	7,20%	KO > KD
Number of siliques from the main axis	Fig. 3m	31,6	40,4	37,8	27,80%	19,60%	KO > KD
Number of siliques/5cm range from the main axis	Fig. 3n	5,2	6,53	6,26	25,60%	20,40%	KO > KD
Total weight of seeds	Fig. 3o	0,1001	0,1495	0,1491	49,30%	49%	KO = KD
Total chlorophyll 11 DAG	Fig. 8b	0,005	0,01	0,007	100%	40%	KO > KD
Carotenoids 11 DAG	Fig. 8c	0,0008	0,0014	0,001	75%	25%	KO > KD
Total chlorophyll 35 DAG	Fig. 8d	0,09	0,2	0,24	122%	166%	KO < KD
Carotenoids 35 DAG	Fig. 8e	0,013	0,024	0,019	85%	46%	KO > KD
Net CO2	Fig. 8f	0,66	1,22	1,11	84%	68%	KO > KD
Transpiration	Fig. 8g	1,58	2,1	2,02	33%	27,80%	KO > KD
Whole -canopy conductance	Fig. 8h	119	153	157	28,60%	31,90%	KO < KD
Water use efficiency	Fig. 8i	0,41	0,61	0,58	48%	41,50%	KO > KD
FV/FM	Fig. 8j	0,59	0,71	0,68	20,30%	15,30%	KO > KD
NPQ	Fig. 8k	0,57	0,7	0,6	22,80%	5,30%	KO > KD
Soluble sugar 11 DAG	Fig. S15a	15,8	25,6	24,1	62,30%	52,50%	KO > KD
Starch 11 DAG	Fig. S15b	362	297	290	-17,90%	-19,90%	KO > KD
Soluble sugar 35 DAG	Fig. S15c	13,5	24,87	25,88	84,70%	91,60%	KO < KD
Starch 35 DAG	Fig. S15d	93,7	58,9	67,9	-37,10%	-27,60%	KO > KD
Soluble sugar silique	Fig. S15e	33,3	36,09	30,05	8,37%	-9,76%	KO > KD
Starch silique	Fig. S15f	283,4	293,4	336,2	3,50%	18,63%	KO< KD
